# Receptor-tuned IL-2 mutein decouples Treg expansion from ILC2-driven cutaneous inflammation

**DOI:** 10.1172/JCI207126

**Published:** 2026-09-29

**Authors:** Anupama Sahoo, Cody Moorman, Mina Tsenkova, Yi Jing, Alexis Valdovinos, Xin Luo, Shiping Lu, Songyu Wang, Renee R. Hukkanen, Madeline Fort, Helen S. Tang, Ronya Primack, Shweta Mandavalli, Weiwen Deng

**Affiliations:** 1 Inflammation Research, Amgen Inc., South San Francisco, California, USA.; 2 Amgen R&D Postdoctoral Fellows Program, South San Francisco, California, USA.; 3 Discovery Sciences, Advanced Technologies & AI, Amgen Inc., South San Francisco, California, USA.; 4 Discovery Protein Science, Amgen Inc., South San Francisco, California, USA.; 5 Translational Safety & Risk Sciences, Amgen Inc., Cambridge, Massachusetts, USA.; 6 Pharmacokinetics and Drug Metabolism, Amgen Inc., South San Francisco, California, USA.

**Keywords:** Immunology, Inflammation, Innate immunity, Therapeutics, Tregs

## Abstract

Low-dose IL-2 and IL-2 muteins are being developed to expand Tregs for autoimmune disease therapy, but injection site reactions (ISRs) remain frequent and poorly understood. Here, we showed that IL2Mut24, a murine surrogate of IL-2 receptor α–biased (IL-2Rα–biased, IL-2Rα is also known as CD25) IL-2 mutein, enhanced Treg expansion and yet paradoxically exacerbated cutaneous inflammation compared with WT IL-2. Using immunodeficient mice, antibody blockade, and adoptive transfer, we identified group 2 innate lymphoid cells (ILC2s) as key drivers of IL-2–induced skin inflammation, defining a CD25-dependent innate activation axis that constrains immune tolerance. In cynomolgus monkeys, the CD25-biased IL-2 mutein efavaleukin alfa promoted greater peripheral Treg expansion than aldesleukin (recombinant human IL-2) but was accompanied by transient increases in IL-5 and dose-dependent ISRs, indicating conservation of this innate inflammatory program across species. To improve the therapeutic window, we engineered receptor clamps by linking IL2Mut24 to antibodies against CD25 to restrict IL-2 access to CD25. This receptor-tuned IL-2 preserved Treg selectivity, suppressed ISRs, and outperformed IL2Mut24 in experimental autoimmune encephalomyelitis by restraining Th17 responses. These findings revealed a conserved innate mechanism underlying IL-2–associated toxicity and establish receptor tuning as a strategy to improve the safety and efficacy of IL-2–based immunotherapy.

## Abstract 2

Redesigning an autoimmune therapy boosts protective immune cells while stopping skin irritation, making the treatment both safer and more effective

## Graphical abstract

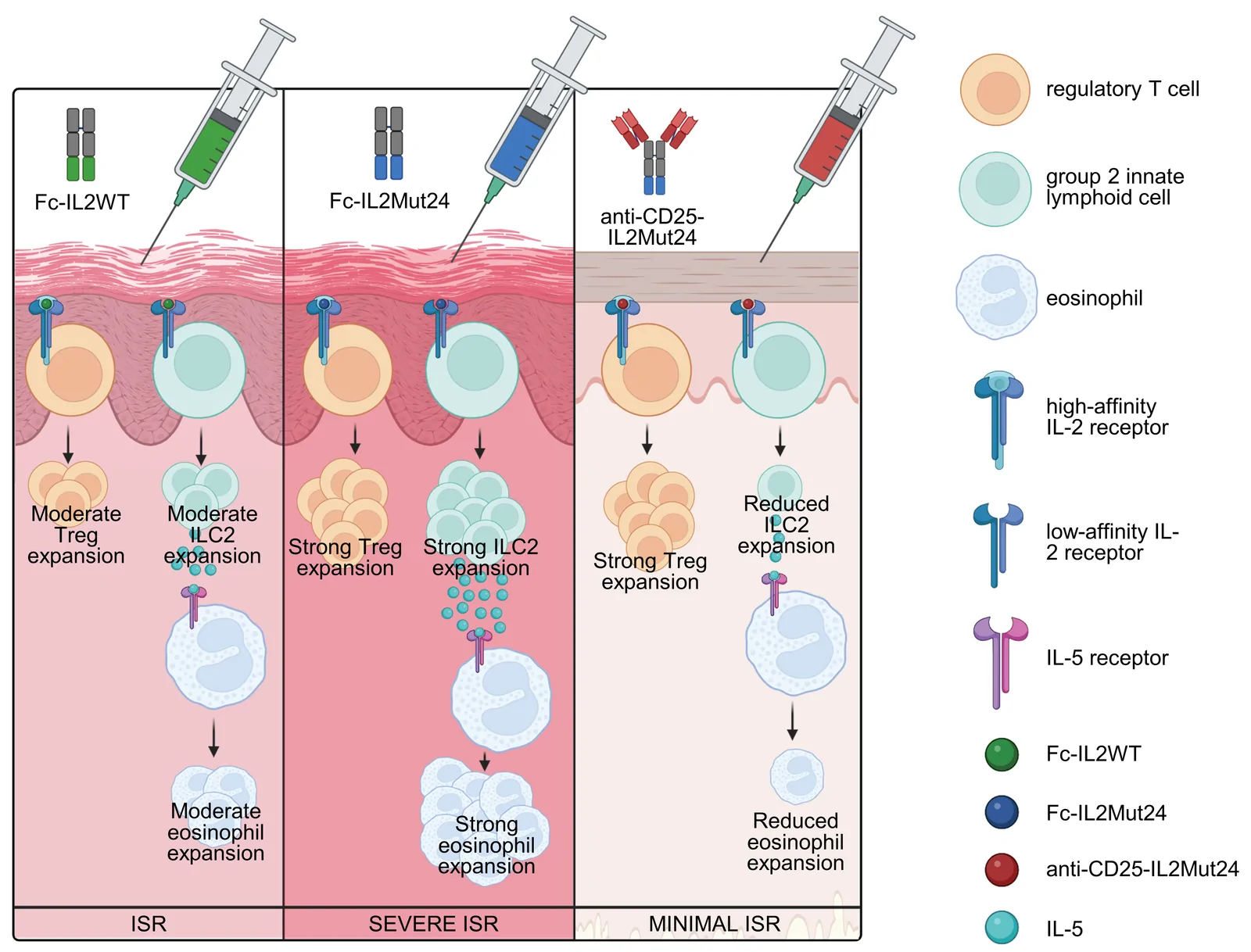

## Introduction

IL-2 is a pleiotropic cytokine that orchestrates immune activation and immune tolerance (1). Its biological outcome is dictated by differential expression of the IL-2 receptor (IL-2R) across immune subsets. IL-2 signals through either an intermediate-affinity receptor composed of IL-2Rβ (CD122) and the common γ chain (γc) or a high-affinity receptor that additionally includes IL-2Rα (CD25) (2). Tregs constitutively express CD25, allowing them to efficiently scavenge limited IL-2 through the high-affinity IL-2R and maintain immune tolerance (3). This receptor hierarchy underlies low-dose IL-2 strategies designed to preferentially expand Tregs in autoimmune and inflammatory diseases (4, 5).

Despite consistent Treg expansion in clinical studies, injection site reactions (ISRs) remain among the most common adverse events reported across IL-2–based therapy programs (6–11). These reactions present as erythema, induration, or painful nodules at the site of administration and occasionally necessitate dose reductions or treatment discontinuation (12, 13). However, the cellular mechanisms driving IL-2–associated cutaneous inflammation remain poorly defined despite their prevalence.

To enhance Treg selectivity and improve pharmacokinetics, engineered IL-2 molecules incorporate attenuating mutations and long-acting formats such as PEGylation (14) or fragment crystallizable–fusion (Fc-fusion) constructs, including efavaleukin alfa (AMG592) (15). The V91K substitution in AMG592 reduces signaling potency and increases dependence on CD25 engagement, thereby favoring Treg expansion over activation of effector T cells and NK cells (15, 16). IL2Mut24, a murine IL-2 mutein bearing mutations at the IL-2Rβ interface, similarly confers CD25 bias and enhances Treg selectivity in vivo (17). Whether such CD25-biased designs modify inflammatory signaling within tissues and thereby influence ISR liability remains unresolved.

Although IL-2 was initially believed to primarily act on T cells and NK cells, recent studies have identified group 2 innate lymphoid cells (ILC2s) as an additional IL-2–responsive population (6, 18). Upon activation, ILC2s can express high levels of CD25 and produce type 2 cytokines, such as IL-4, IL-5, and IL-13, contributing to allergic inflammation and immune homeostasis (19). Low-dose IL-2–anti–IL-2 antibody complexes activate ILC2s and IL-5–dependent eosinophilia (6, 7), and WT IL-2 can induce CD25-dependent activation of innate immune cells, including ILC2s, promoting cutaneous inflammation (7). These observations implicate CD25-dependent innate pathways in IL-2–driven inflammatory responses.

In this study, by directly comparing WT and CD25-biased IL-2 variants in mice and cynomolgus monkeys, we uncovered a CD25-dependent innate inflammatory axis driven by ILC2 activation that constrained IL-2 tolerability. We further showed that receptor-guided engineering could uncouple immune regulation from inflammatory toxicity, establishing a design principle for next-generation cytokine therapies.

## Results

### CD25-biased IL2Mut24 amplifies cutaneous inflammation.

To model and investigate IL-2–associated ISRs observed clinically, C57BL/6 mice were injected s.c. twice weekly for 4 weeks with PBS or mouse surrogate Fc-fused IL-2 proteins (Fc-IL2Mut24 or Fc-IL2WT) (Figure 1A). Repeated dosing with Fc-IL2Mut24 induced severe cutaneous inflammation characterized by erythema, swelling, hair loss, and occasional ulceration, resulting in significantly elevated ISR scores (Figure 1, B and C). In contrast, Fc-IL2WT induced only mild-to-moderate reactions, and PBS-treated controls displayed no visible inflammation (Figure 1, B and C). A similar differential response to Fc-IL2Mut24 versus Fc-IL2WT was also observed in BALB/c mice (Supplemental Figure 1, A–C; supplemental material available online with this article; https://doi.org/10.1172/JCI207126DS1).

Pharmacokinetics analysis revealed prolonged plasma persistence of Fc-IL2Mut24 relative to Fc-IL2WT (Figure 1D). Tissue biodistribution analysis over 72 hours after a single injection further demonstrated sustained accumulation of Fc-IL2Mut24 in both injection site and noninjection site skin (Figure 1, E and F). These findings suggest that enhanced and prolonged local exposure contributes to the severity and persistence of Fc-IL2Mut24–induced cutaneous inflammation.

To assess the impact of host immune context in the induction of ISRs, we compared responses across Th1-biased C57BL/6 (20), Th2-biased BALB/c (20), and autoimmune-prone C3H/HeJ strains (21). Fc-IL2Mut24 induced ISRs in BALB/c mice at a low dose (28.9 pmol/dose), while C57BL/6 mice were largely resistant (Supplemental Figure 2, A–C). At a higher dose (289 pmol/dose), both strains developed substantial inflammation without significant changes in body weight, with C57BL/6 mice exhibiting greater ISR severity (Supplemental Figure 2, C and D). C3H/HeJ mice developed particularly severe ISRs following Fc-IL2Mut24 treatment, including progressive hair loss and ulceration, accompanied by >10% body weight loss (Supplemental Figure 2, E–G).

Collectively, these results demonstrate that CD25-biased IL-2 mutein amplifies cutaneous inflammatory responses across genetically distinct backgrounds, with severity modulated by dose and host immune context. Therefore, Fc-IL2Mut24–induced ISR provides a reproducible model of IL-2–associated skin toxicity.

### CD25-biased IL2Mut24 drives parallel expansion of Tregs and type 2 innate immune responses.

To define the immune dynamics underlying the distinct ISR profiles of Fc-IL2Mut24 and Fc-IL2WT, we performed a time-course analysis following a single s.c. administration of 116 pmol selected from a previously established Treg-expanding range in C57BL/6J mice (Figure 2A) (17). At day 2 after injection, Fc-IL2WT increased serum IL-6, TNF, and IL-4 relative to PBS and Fc-IL2Mut24, consistent with its broader proinflammatory activity (Figure 2, B–D). In contrast, Fc-IL2Mut24 treatment induced minimal IL-6 or TNF but selectively elevated serum IL-5, suggesting a distinct cytokine bias toward type 2 immunity (Figure 2E).

Analysis of splenic compartments revealed parallel Treg and ILC2 kinetics. Both populations expanded following Fc-IL2Mut24 treatment, peaked at day 4, and contracted toward baseline by day 7, with greater day 4 responses than those induced by Fc-IL2WT (Figure 2, F and G). Although less abundant overall, ILC2s showed a particularly pronounced Fc-IL2Mut24 response, whereas Fc-IL2WT induced a smaller response that persisted through day 7. Eosinophils followed similar kinetics, peaking at day 4 and declining by day 7 (Figure 2H). The earlier rise in serum IL-5 is consistent with activation of the ILC2-eosinophil axis preceding maximal cellular expansion.

Collectively, these data indicate that sustained CD25-biased IL-2 signaling drives a temporally distinct immunological trajectory, characterized by transient Treg expansion followed by preferential amplification of ILC2 and eosinophilic responses. This temporal shift toward type 2–skewed innate activation may underlie the development of ISRs during sustained IL-2 exposure.

### Fc-IL2Mut24 induces a CD25-dependent type 2 inflammatory program in skin.

To define the local immune response associated with IL-2–induced ISR, we characterized innate lymphoid cells (ILC) subsets and cytokines in injection site skin from C57BL/6 mice treated with PBS, Fc-IL2WT, or Fc-IL2Mut24. At 69 pmol, a pharmacodynamically active Treg-expanding dose, Fc-IL2Mut24 also induced more severe ISRs than Fc-IL2WT (Figure 3A) (22). Total ILC numbers increased numerically following IL-2 treatment, most prominently with Fc-IL2Mut24 (Figure 3B). CD25^+^ frequencies increased selectively among ILC2s, with the strongest response to Fc-IL2Mut24, but were unchanged among ILC1s and ILC3s (Supplemental Figure 3, A–C). Both IL-2 proteins increased cutaneous CD25^+^ Tregs and ILC2s: Treg accumulation was comparable while Fc-IL2Mut24 elicited greater ILC2 accumulation, leading to a lower Treg/ILC2 ratio (Figure 3, C–E). A similar ILC2-biased response was observed in the lung, where ILC2s were the predominant expanding ILC population and showed the most pronounced increase in CD25 expression following IL-2 treatment (Supplemental Figure 3, D–G).

Skin cytokine profiling showed treatment-associated elevation in TNF, IFN-γ, IL-6, and IL-5 (Supplemental Figure 3, H–K). Only Fc-IL2WT significantly increased IL-4 relative to PBS, whereas IL-13 induction did not differ between IL-2 molecules (Supplemental Figure 3, L–M). Among type 17–associated cytokines, IL-17A and IL-23 showed upward trends, whereas IL-17A/F and IL-22 were unchanged (Supplemental Figure 3, N–Q). Together, these findings identify ILC2s as the most CD25-responsive ILC subset in injection site skin and support a CD25-dependent type 2 inflammatory program.

To distinguish the requirement for CD25-dependent signaling from that of the IL-5–eosinophil effector axis, C57BL/6 mice were treated with Fc-IL2Mut24 in combination with anti–IL-5, anti-CD25, or isotype control antibodies (Figure 3F). IL-5 neutralization modestly reduced ISRs, whereas anti-CD25 completely prevented ISRs (Figure 3G and Supplemental Figure 4A). Histopathology confirmed that only anti-CD25 significantly reduced cutaneous inflammation (Figure 3H and Supplemental Figure 4, B and C). Consistently, cytokine profiling of skin tissue showed minimal reduction in IL-1β, TNF, and IL-5 following anti–IL-5 treatment, but near-complete suppression with anti-CD25 (Supplemental Figure 4, D–F). Splenic immune profiling further revealed that anti–IL-5 treatment selectively eliminated eosinophils, whereas anti-CD25 reduced both ILC2s and eosinophils (Figure 3, I and J). Thus, CD25-dependent signaling is a central requirement for Fc-IL2Mut24–induced ISR, whereas the IL-5–eosinophil axis contributes to, but does not fully account for, the inflammatory response, consistent with previous reports (7).

### ILC2s drive IL-2–induced cutaneous inflammation independent of adaptive immunity.

To determine whether adaptive immunity was required for ISR development, *Rag2^–/–^* mice, which lack T and B cells, were treated with IgG-IL2Mut24, generated by linking IL2Mut24 to a nontargeting mouse IgG (Supplemental Figure 5A). IgG-IL2Mut24 induced ISRs in C57BL/6 mice similarly to Fc-IL2Mut24 (Supplemental Figure 5, B and C). Notably, *Rag2^–/–^* mice developed more severe ISRs than immunocompetent C57BL/6, indicating that innate immune cells are sufficient to mediate ISR and that Tregs may partially restrain pathology in C57BL/6 mice (Supplemental Figure 5, B and C). In contrast, *Rag2^–/–^γc^–/–^* mice, which lack all γc-dependent populations lineages, including ILC2s, failed to develop ISR upon IgG-IL2Mut24 treatment, indicating that γc-dependent innate lymphocytes are required for ISR development (Supplemental Figure 5, B and C) (23).

To directly assess the contribution of Tregs in restraining ISR severity, T cells from Foxp3-DTR donor mice were transferred into *Rag2^–/–^* recipients before repeated IgG-IL2Mut24 administration (Supplemental Figure 6A), allowing selective depletion of Foxp3^+^ Tregs within a reconstituted T cell compartment after ISR onset. Diphtheria toxin–mediated (DT-mediated) Treg depletion resulted in progressive worsening of ISR and substantial body weight loss compared with that in nondepleted recipients (Supplemental Figure 6, B–D). These findings indicate that Tregs within the transferred T cell population limit ISR progression and systemic morbidity, although their presence was insufficient to prevent ISR development.

To define the cytokine pathways contributing to Fc-IL2Mut24–induced ISR independently of adaptive immunity, we next performed antibody-blockade studies in *Rag2^–/–^* mice, with anti-CD25 included as a control (Figure 4A and Supplemental Figure 7A). Neutralization of IFN-γ, IL-17A, or IL-4 did not attenuate ISR, whereas IL-5 blockade provided partial protection and IL-13 blockade produced the greatest attenuation among the cytokines tested (Figure 4B and Supplemental Figure 7, B–D). Consistent with its effect on ISR severity, IL-13 blockade reduced IFN-γ, TNF, IL-6, and IL-1β in injection site skin and decreased the frequency of CD25^+^ ILC2s without significantly altering total ILC2 numbers (Figure 4, C and D, and Supplemental Figure 7, E–H). Anti-CD25 also completely prevented ISR development in *Rag2^–/–^* mice (Supplemental Figure 7, B and C). Together, these findings identify CD25-dependent innate activation as a key driver of ISR, with IL-13 acting as a prominent effector and the IL-5–eosinophil axis serving as a contributory amplifier.

To determine which γc-dependent innate lymphocyte population could restore susceptibility to ISRs, IL-33–expanded ILC2s harvested from the lungs and IL-15 plus IL-12–expanded ILC1s and NK cells isolated from the livers of *Rag2^–/–^* mice were adoptively transferred into *Rag2^–/–^γc^–/–^* mice, followed by Fc-IL2Mut24 administration (Figure 4E, Supplemental Figure 8, A and B, and Supplemental Figure 9, A–C) (24, 25). In addition, *Rag2^–/–^* mice receiving Fc-IL2Mut24 served as controls (Figure 4E). Among the populations we tested, only engrafted ILC2s restored ISR development in *Rag2^–/–^γc^–/–^* recipient mice, whereas transfer of ILC1s or NK cells was not able to restore ISR development (Figure 4F, Supplemental Figure 8C, and Supplemental Figure 9, D and E). Inflammation in ILC2-reconstituted recipients localized to injection sites, unlike the more widespread reactions observed in *Rag2^–/–^* mice receiving Fc-IL2Mut24 (Supplemental Figure 8C). The transferred ILC2s were functionally active, as evidenced by downstream eosinophil expansion (Figure 4, G–I). Notably, Fc-IL2Mut24 not only expanded eosinophils but also recruited neutrophils in ILC2-transferred *Rag2^–/–^γc^–/–^* mice (Figure 4J). Consistent with these cellular changes, elevated levels of IL-5, IL-6, TNF, and keratinocyte chemoattractant/growth-regulated oncogene (KC/GRO) were detected in the skin lysates of *Rag2^–/–^* mice as well as ILC2-recipient *Rag2^–/–^γc^–/–^* mice upon Fc-IL2Mut24 injection (Supplemental Figure 8, D–G).

Collectively, these findings establish CD25-dependent innate signaling as a critical driver of IL-2–induced cutaneous inflammation. CD25^+^ ILC2s function as upstream initiators of a cytokine network that recruits eosinophils and neutrophils, defining an innate inflammatory circuit that constrains IL-2 tolerability.

### CD25-biased IL-2 mediates innate activation and cutaneous inflammation in cynomolgus monkeys.

To assess whether the CD25-dependent innate activation axis identified in mice is conserved in a translational setting, we evaluated AMG592, a human IL-2 mutein engineered to preferentially engage CD25, in cynomolgus monkeys (16). Aldesleukin (recombinant human IL-2) was included as a clinical comparator to distinguish the pharmacodynamic (PD) consequences of CD25-biased versus conventional IL-2R engagement (Figure 5A).

In study 118075, male cynomolgus monkeys received two treatment cycles of AMG592 or aldesleukin. Expansion of circulating Tregs was observed following the second dosing cycle, which used higher doses, reaching maximal levels 5–8 days later (days 18, 19, and 21) (Figure 5B). AMG592 also induced a marked increase in serum IL-5 levels following second dosing, peaking around days 17–18 during the second cycle and resolving thereafter (Figure 5C). Across the tested dose range, AMG592 produced a higher Treg response and IL-5 peak than aldesleukin (Figure 5, B and C). However, aldesleukin induced broader cytokine responses, including IFN-γ, GM-CSF, IL-10, and IL-6, whereas AMG592 triggered minimal increases in these cytokines (Figure 5, D–G). Together, these data indicate that CD25-biased IL-2 signaling is associated with selective IL-5 induction followed by robust Treg expansion, consistent with observations in the murine model (Figure 2).

In a separate repeated-dose toxicology study (study 118065), histopathologic evaluation of s.c. injection sites after 3 months of weekly AMG592 dosing in cynomolgus monkeys demonstrated dose-dependent inflammatory changes localized to the administration site (Figure 5, H and I). Epidermal remodeling, characterized by minimal-to-mild hyperkeratosis and acanthosis, increased in both incidence and severity with dose and mirrored findings observed in murine models. In the dermis, mid- and high-dose animals exhibited increased mononuclear cell infiltrates, with occasional mixed-cell infiltrates at higher exposures. These findings were generally minimal to mild in severity and are consistent with localized immune activation associated with pharmacologic activity.

Together, these data indicate that CD25-biased IL-2 engagement in nonhuman primates recapitulates the transient type II innate activation and localized inflammatory responses observed in murine models, supporting cross-species conservation of this CD25-dependent inflammatory axis.

### Epitope-specific CD25 engagement tunes IL-2 signaling strength and tissue immune responses.

To modulate CD25-dependent IL-2 signaling, we generated anti-CD25-IL2Mut24 fusion proteins by fusing IL2Mut24 to 3 monoclonal antibodies recognizing distinct CD25 epitopes with different antagonistic properties and affinities: 7D4 (nonantagonist, *K_D_* = 0.3 nM), PC61 (partial antagonist, *K_D_* = 0.3 nM), and 3C7 (full antagonist, *K_D_* = 7.6 nM) (Figure 6A) (26). IgG-IL2Mut24 served as a control. Structural modeling revealed that the PC61 epitope partially overlaps with the IL-2–CD25 interface, consistent with partial antagonism, while the 7D4 epitope does not (Supplemental Figure 10, A–C). We hypothesized that intramolecular fusion of IL2Mut24 to anti-CD25 antibodies would enforce local coengagement of CD25 while competitively limiting high-affinity receptor assembly, thereby quantitatively modulating downstream signaling in a clone-dependent manner. This “receptor clamp” strategy was designed to favor the engagement of CD25^+^ Tregs while limiting maximal IL-2 signaling capacity.

IL-2 bioactivity was first assessed in proliferation assays using CD25^+^ CTLL-2 cells, an IL-2–dependent mouse T cell line (Figure 6B). All IL-2 constructs induced dose-dependent proliferation, with magnitude following the rank: Fc-IL2WT > Fc-IL2Mut24 = IgG-IL2Mut24 > 7D4-IL2Mut24 = 3C7-IL2Mut24 > PC61-IL2Mut24. To determine whether this effect required intramolecular colocalization, anti-CD25 antibodies were codelivered with IgG-IL2Mut24 (Figure 6C). For 7D4 and 3C7, free antibody codelivery recapitulated their respective fusion activities. In contrast, free PC61 completely blocked IL-2 signaling likely due to its high affinity and antagonism. Thus, IL-2 signaling amplitude is tunable according to antibody epitope and affinity.

Because CD25 expression on ILC2s is dynamically regulated and can be rapidly upregulated following activation (27), we first assessed its kinetics in primary mouse ILC2s. Fc-IL2WT, Fc-IL2Mut24, and IgG-IL2Mut24 progressively increased CD25 surface intensity over 8 hours, whereas PC61-IL2Mut24 and 3C7-IL2Mut24 resulted in more limited induction (Supplemental Figure 11A), consistent with an IL-2–driven amplification of ILC2 responsiveness. In proliferation assays, IgG-IL2Mut24 induced robust, dose-dependent ILC2 proliferation, whereas 7D4-, PC61-, and 3C7-IL2Mut24 elicited minimal responses (Figure 6D). Free PC61 or 7D4 codelivered with IgG-IL2Mut24 similarly suppressed proliferation, whereas 3C7 retained partial activity (Figure 6E). Together, these findings indicate that receptor tuning constrains activation-associated CD25 induction and ILC2 proliferation.

The IL2Mut24 fusions displayed broadly similar serum profiles, with peak concentrations at 24 hours followed by a decline through 72 hours, although Fc-IL2WT showed substantially lower concentrations (Supplemental Figure 11B). IL-2 fusion proteins were detectable in both injection site and noninjection site skin at 72 hours but varied among constructs: 7D4- and 3C7-IL2Mut24 showed relatively high levels, while PC61-IL2Mut24 was comparable to Fc-IL2Mut24 and IgG-IL2Mut24 at both injection and noninjection sites (Supplemental Figure 11C). In contrast, Fc-IL2WT remained low in both compartments. Together, these findings indicate broadly comparable systemic exposure and varying tissue distribution among the IL2Mut24 fusion proteins.

To evaluate in vivo PD activity, C57BL/6J mice received 69 pmol of each IL-2 fusion or codelivered anti-CD25 antibody with IgG-IL2Mut24 (Figure 6F). IgG-IL2Mut24, PC61-IL2Mut24, and 3C7-IL2Mut24 increased Treg frequencies and absolute numbers across spleen, mesenteric lymph nodes, lung, and visceral adipose tissue on day 4 (Figure 6, G and H, and Supplemental Figure 11, D–I). In contrast, 7D4-IL2Mut24 produced weaker Treg expansion. Codelivered PC61 and IgG-IL2Mut24 failed to expand Tregs, whereas 3C7 codelivery retained activity in terms of Treg frequency in secondary lymphoid tissues but was less effective in peripheral compartments than the corresponding fusion (Supplemental Figure 11, D–I). This suggests the importance of molecular fusion for coordinated delivery and CD25 engagement.

In the repeated-dose ISR model, both 7D4-IL2Mut24 and PC61-IL2Mut24 significantly reduced ISR severity, whereas 3C7-IL2Mut24 induced inflammation comparable to that of IgG-IL2Mut24 (Figure 6, I and J, and Supplemental Figure 12A). IgG-IL2Mut24 and PC61-IL2Mut24 were further selected for detailed cutaneous profiling because they represent benchmark versus attenuated IL-2 signaling. A murine IgG-LALAPG control did not induce ISR (Supplemental Figure 12B), arguing against a nonspecific inflammatory effect of the Fc-containing protein scaffold. Consistent with markedly reducing ISR severity, PC61-IL2Mut24 maintained the increase in cutaneous CD25^+^FoxP3^+^ Tregs comparable to IgG-IL2Mut24, while markedly reducing the frequency of CD25^+^ ILC2s and lowering TNF and IFN-γ in injection site skin (Supplemental Figure 12, C–H). PC61-IL2Mut24 reduced CD25 surface intensity on both Tregs and ILC2s compared with IgG-IL2Mut24, although CD25 induction remained more pronounced in Tregs (Supplemental Figure 12, E and F). Together, these findings indicate that PC61-IL2Mut24 preferentially constrains the cutaneous ILC2 response while maintaining Treg expansion.

Collectively, these results validate the receptor clamp concept and show that epitope-specific fusion of IL2Mut24 to anti-CD25 enables quantitative and tissue-relevant modulation of IL-2 signaling, demonstrating that tuning CD25 engagement can preserve Treg expansion while limiting inflammatory toxicity.

### Receptor-tuned IL-2 reshapes the cutaneous immune transcriptional landscape.

To define how receptor tuning alters local immune responses, we performed single-cell RNA sequencing (scRNA-seq) of injection site tissue. BALB/c-Foxp3-GFP mice received a single s.c. dose of Fc-IL2Mut24 or PC61-IL2Mut24, and injection site skin was harvested on day 4. Cell suspensions were sorted into 3 populations for scRNA-seq: CD45^+^TCRβ^+^GFP^+^(Treg), CD45^+^TCRβ^+^GFP^–^, and CD45^+^ TCRβ^–^ (Supplemental Figure 13A).

scRNA-seq of these compartments revealed treatment-specific remodeling across cell populations. Within the CD45^+^TCRβ^+^GFP^–^ cell population, Fc-IL2Mut24 increased the Th2 fraction with a modest rise in type 1 cytotoxic T cells (Tc1) versus isotype control (Supplemental Figure 13, B and C). In contrast, PC61-IL2Mut24 decreased Th2/Tc1 and preferentially enriched CCR7^+^CD4^+^ and CCR7^+^CD8^+^ populations. Within the Treg compartment (CD45^+^TCRβ^+^GFP^+^), Fc-IL2Mut24 increased the relative representation of activated and cycling states, whereas PC61-IL2Mut24 retained a substantial activated Treg population but shifted the composition toward a more resting and less proliferative phenotype (Figure 7, A and B, and Supplemental Figure 13D). In the CD45^+^TCRβ^–^ fraction, Fc-IL2Mut24 shifted composition away from B cell lineages toward ILC2 with a concomitant rise in ILC1s, whereas PC61-IL2Mut24 restrained ILC2 enrichment and preserved higher B cell representation (Figure 7, C and D, and Supplemental Figure 13E).

Integrated principal component analysis and heatmaps of selected differentially expressed genes revealed treatment-specific transcriptional remodeling in skin Tregs and ILC2s. In Tregs, both Fc-IL2Mut24 and PC61-IL2Mut24 separated from isotype, with PC61-IL2Mut24 exhibiting a tempered profile relative to Fc-IL2Mut24 (Figure 7E). Fc-IL2Mut24 strongly induced interferon-responsive and cell-cycle programs, including *Ifitm1/2/3*, *Aspm*, *Kif15*, and *Clspn*, consistent with enrichment of activated and cycling Treg states (Figure 7F) (22, 28–31). PC61-IL2Mut24 retained a substantial activated Treg population but showed reduced expression of these interferon-responsive and proliferative genes, together with a distinct module marked by *Epas1* and *Penk*, suggesting a less proliferative, tissue-adapted state (Figure 7F) (32–34). In ILC2s, all groups diverged primarily along PC1 (22% variance), and PC61-IL2Mut24 occupied a position between isotype and Fc-IL2Mut24, indicating distinct treatment-associated transcriptional states (Figure 7G). Consistently, Fc-IL2Mut24 upregulated inflammatory, stress-response, and chemotaxis-associated signatures in ILC2s (e.g., *Hspa1a*, *Ifitm1/2/3*, *Nlrp3*, *Ccl5*) (31, 35–38). PC61-IL2Mut24 attenuated many of these changes while inducing a distinct module marked by *Smpdl3a*, *Fchsd2*, *Rab18*, *Vamp3*, together with cytoskeletal genes such as *Tuba1c*, *Tubb4b*, and *Palld*, indicating altered immunometabolic, membrane-trafficking and cytoskeletal programs (Figure 7H) (39–42). Thus, PC61-IL2Mut24 qualitatively reprogrammed, rather than simply normalized, the ILC2 response. In contrast, CD4^+^ and CD8^+^ conventional T cells showed no clear separation among conditions (Supplemental Figure 13, F and G).

Collectively, these data demonstrate that Fc-IL2Mut24 markedly remodeled the skin immune landscape, expanding activated/proliferative Tregs, Th2/Tc1 subsets, and inflammatory ILC2 programs. In contrast, PC61-IL2Mut24 preserved activated Treg enrichment while limiting Treg cycling and constraining ILC2 enrichment and inflammatory programming. These findings indicate that quantitative modulation of CD25 engagement differentially reshapes adaptive and innate immune compartments in vivo.

### Receptor-tuned IL-2 enhances suppression of experimental autoimmune encephalomyelitis onset.

To evaluate whether receptor tuning improves IL-2–mediated immune tolerance in vivo, we evaluated PC61-IL2Mut24 in experimental autoimmune encephalomyelitis (EAE), a Treg-sensitive, CD4^+^ T cell–mediated model of CNS autoimmunity (43). Mice received i.p. injections of 69 pmol of isotype control, IgG-IL2Mut24, or PC61-IL2Mut24 on an “all” regimen (days –2, 0, 2, 6, 9, 13, 16), PC61-IL2Mut24 on an “early” regimen (days 2 and 6), or PC61-IL2Mut24 on a “late” regimen (days 16 and 19) relative to EAE induction, and anti-CD4 served as a control (Figure 8A). Both IgG-IL2Mut24 and PC61-IL2Mut24 administered on the “all” regimen significantly reduced clinical EAE scores and weight loss compared with isotype control, with PC61-IL2Mut24 demonstrating superior efficacy (Figure 8, B and C). In addition, a clear regimen response was observed: PC61-IL2Mut24 “all” treatment significantly reduced cumulative EAE scores compared with “early” treatment, which, in turn, outperformed “late” treatment, the latter showing no therapeutic benefit (Figure 8D). PC61-IL2Mut24 also reduced maximal clinical scores more effectively than IgG-IL2Mut24, indicating enhanced therapeutic efficacy (Supplemental Figure 14A). Analysis of CNS-infiltrating T cells at study termination revealed that both IgG-IL2Mut24 and PC61-IL2Mut24 markedly reduced T cell infiltration (Figure 8E and Supplemental Figure 14B) and decreased the frequency of IFN-γ^+^ CD4^+^ T cells, with PC61-IL2Mut24 producing the more pronounced effect (Figure 8F). Only PC61-IL2Mut24 significantly reduced IL-17A^+^ CD4^+^ T cells (Figure 8G), whereas IgG-IL2Mut24 preferentially increased CD25 and 4-1BB expression on conventional CD4^+^ T cells (Figure 8H and Supplemental Figure 14C). In addition, PC61-IL2Mut24 significantly reduced the frequency of MOG-tetramer^+^ CD4^+^ T cells (Figure 8I) while increasing the proportion of Tregs within the MOG-tetramer^+^ CD4 T cells compared with isotype-treated mice and increasing PD-1 expression on CD4^+^ Tregs (Figure 8J and Supplemental Figure 14D).

Together, these results show that receptor-tuned PC61-IL2Mut24 confers superior disease control compared with IgG-IL2Mut24 and that early initiation with sustained dosing is required for durable immunomodulation in EAE. This enhanced efficacy was associated with limited activation of pathogenic conventional CD4^+^ T cells while preserving Treg responses. These findings support quantitative tuning of CD25-dependent IL-2 signaling as a strategy to shift antigen-specific immunity toward tolerance.

## Discussion

Our study reveals an unanticipated divergence between Treg-selective IL-2 engineering and local inflammatory outcomes, highlighting a key challenge in developing next-generation IL-2 therapies. IL2Mut24 is a CD25-biased IL-2 mutein designed to preferentially expand Tregs and Fc-IL2Mut24 confers prolonged systemic and tissue exposure. Despite this regulatory design, Fc-IL2Mut24 paradoxically exacerbates cutaneous inflammation compared with Fc-IL2WT. Because these molecules were compared at the same molar dose but were not matched for systemic or cutaneous exposure, the difference in ISR severity cannot be attributed solely to CD25 bias; altered receptor pharmacology and prolonged exposure likely both contribute. These findings highlight a key therapeutic dilemma: although Treg expansion promotes immune tolerance, concurrent activation of CD25-expressing ILC2s can drive type 2 inflammation, particularly in barrier tissues such as the skin (6, 15, 17).

Notably, cynomolgus monkey studies indicate that this safety-efficacy trade-off may extend across species. As the toxicology study of AMG592 included vehicle but not a matched protein control, nonspecific injection-related effects cannot be fully excluded; however, these studies were not designed to resolve tissue ILC2 activation directly. Thus, the concordance among Treg expansion, IL-5 induction, and localized pathology supports but does not definitively establish a pharmacology-driven, ILC2-dependent mechanism in nonhuman primates. These observations remain relevant to the clinical development of CD25-biased IL-2 therapies such as AMG592 and rezpegaldesleukin (14, 15). Our data raise the possibility that sustained CD25 engagement may couple regulatory expansion with local innate immune activation, potentially limiting the therapeutic window of CD25-biased IL-2 therapies in humans.

Tregs are reported to suppress inflammation partially by acting as an IL-2 “sink”: constitutive CD25 expression enables them to outcompete effector T cells and ILCs for limited IL-2 (44). However, sustained ligand availability may erode this advantage as effector T cells and ILC2s upregulate CD25 and become increasingly responsive to IL-2 (27, 35, 36, 45). Consistent with this model, Fc-IL2Mut24 engaged both cutaneous Tregs and ILC2s, with CD25 expression increasing on ILC2s following activation. The greater ISR severity in *Rag2^–/–^* mice and the subsequent selective Treg-depletion study indicated that Tregs restrain but do not fully prevent inflammation when IL-2 remains available to activated innate populations. PC61-IL2Mut24 instead preserved cutaneous Treg enrichment while reducing ILC2 responses and ISR. The EAE findings extend this principle to a CD4^+^ T cell–driven autoimmune setting, in which early and sustained PC61-IL2Mut24 treatment limited inflammatory CD4^+^ conventional T cell, especially Th17, responses while maintaining Treg activation and increasing Treg representation within the MOG-specific cells. Together, these observations suggest that effective tolerance requires both preservation of regulatory responses and quantitative restriction of IL-2 signaling in the dominant pathogenic CD25^+^ population. The precise basis of this differential control remains to be established, as we did not directly assess the susceptibility of activated ILC2s and conventional T cells to Treg-mediated suppression.

Mechanistically, our data identify an ILC2-centered, but not ILC2-exclusive, model of IL-2–induced cutaneous inflammation. Sustained CD25-dependent signaling appears to preferentially amplify ILC2 responses and engage a broader inflammatory network, whereas adaptive immunity is dispensable for ISR development. The limited effects of IFN-γ or IL-17A blockade, together with the failure of ILC1 or NK cell reconstitution to restore ISR, argue against dominant roles for these pathways under the conditions tested. Nevertheless, because ILC2-specific depletion was not performed and other cell-specific functions were not comprehensively examined, contributions from additional immune populations cannot be excluded.

The divergent blockade phenotypes are consistent with the distinct although overlapping biology of ILC2 cytokines: IL-5 primarily supports eosinophil development and survival; IL-13 preferentially engages structural cell programs associated with inflammatory tissue remodeling; and IL-4 has broader activity on hematopoietic cells and can support macrophage-mediated repair, although IL-4 and IL-13 share signaling through the type II IL-4 receptor (46). The incomplete protection afforded by IL-5 blockade indicates that ISR extends beyond the IL-5–eosinophil axis (47, 48). IL-13 emerged as a prominent mediator in this model and may participate in a tissue feedback circuit that sustains the CD25^+^ ILC2 state. The accompanying neutrophil recruitment and increases in KC/GRO, IL-6, and TNF suggest secondary engagement of myeloid and tissue-resident compartments. Because our single-cell analysis was restricted to CD45^+^ cells and whole-skin lysates do not resolve mediator sources, the contributions of keratinocytes, fibroblasts, and other stromal populations remain undefined. Thus, ILC2s are best viewed as central mediators within a multicellular inflammatory circuit linking sustained IL-2 signaling to barrier-tissue pathology.

To overcome the safety-efficacy trade-off, we developed anti-CD25-IL2Mut24 “receptor-clamp” fusions that modulate IL-2R engagement at the cellular interface. Our data support a model in which epitope-dependent CD25 engagement acts as a signal modulator controlling IL-2 signaling strength. Structural modeling showed that the PC61 epitope partially overlaps the IL-2–CD25 interface. Consistent with a configuration-dependent mechanism, unconjugated PC61 completely blocked IgG-IL2Mut24 activity, whereas covalently linked PC61-IL2Mut24 retained attenuated agonist activity. The distinct behaviors of the 7D4, PC61, and 3C7 constructs indicate that epitope, affinity, molecular configuration, and exposure collectively determine signaling output, rather than antagonistic classification alone. PC61-IL2Mut24 markedly reduced ISR despite broadly comparable measured exposure to IgG-IL2Mut24 fusions, suggesting that exposure alone does not fully account for its improved tolerability. Its preservation of Treg responses with minimal primary ILC2 proliferation supports a signaling-threshold model in which constitutive CD25 expression enables Tregs to respond to attenuated signaling, whereas ILC2s may require stronger or more sustained signals for activation. Single-cell analysis further showed preservation of activated Tregs alongside a constrained and qualitatively remodeled ILC2 response.

Several limitations and translational questions warrant consideration. The murine ISR model intentionally amplifies local inflammation through repeated dosing at a single site and should therefore be viewed as a mechanistic stress-test model rather than a direct reproduction of the typically moderate and transient reactions observed clinically. Neither the Fc-IL2WT versus Fc-IL2Mut24 comparison nor the receptor-clamp studies were formally exposure matched; therefore, equivalence in area under the curve, peak tissue concentration, receptor occupancy, and signaling duration was not established. We also did not perform ILC-specific depletion, and lineage-reconstitution experiments used cytokine-expanded, preconditioned cells rather than naive populations. The scRNA-seq studies were performed in BALB/c-Foxp3-GFP mice, whereas most mechanistic ISR experiments used C57BL/6 background mice; therefore, strain-specific transcriptional responses and differences in cellular composition cannot be excluded. In addition, the cynomolgus studies had small group sizes, lacked a matched protein control, and did not directly assess tissue ILC2s. Although PC61-IL2Mut24 attenuated exaggerated IL-2–driven ILC2 responses without reducing ILC2s below baseline, dedicated challenge studies will be needed to determine whether receptor tuning affects host defense. Validation in primary human Tregs, ILC2s, and skin systems using human receptor-clamp molecules will also be required to establish the translational relevance of the proposed signaling-threshold mechanism.

Collectively, our findings identify a CD25-dependent, ILC2-centered inflammatory circuit in mice and suggest that related pharmacology may contribute to IL-2–associated local inflammation in nonhuman primates. By quantitatively tuning cytokine-receptor engagement, receptor-clamp IL-2 fusions provide a framework for preserving regulatory benefit while limiting activation of inflammatory CD25^+^ populations. More broadly, these results illustrate how control of signaling amplitude, receptor geometry, and cellular context can reshape immune outcomes and inform the design of next-generation cytokine therapies.

## Methods

### Sex as a biological variable.

Cynomolgus monkey studies included both male and female samples. All mouse experiments were performed with female mice at an age of 6–21 weeks.

### Mice.

C57BL/6, BALB/c, *Rag2^–/–^*, *Rag2^–/–^γc^–/–^*, Foxp3-reporter/DTR, and other indicated mouse strains were obtained from commercial vendors and maintained under specific pathogen–free conditions. Detailed strain information, sources, and husbandry conditions are provided in Supplemental Methods.

### IL-2 treatment and ISR studies.

Mice received the indicated doses of Fc-IL2WT or IL2Mut24 fusion proteins by s.c. injection, with dose and schedule specified in the figure legends. For PD studies, mice received a single dose. For ISR studies, mice were dosed twice weekly for up to 4 weeks and monitored longitudinally for body weight and local reactions. ISR severity was assessed using a composite scoring system incorporating lesion size, bleeding, skin thickening, and inflammation. Detailed dosing and scoring criteria are provided in Supplemental Methods.

### Pharmacokinetics and tissue biodistribution.

C57BL/6J mice received a single s.c. dose of Fc-IL2WT or IL2Mut24 fusion proteins (289 pmol/dose). Plasma and/or skin from injection and noninjection sites were collected at the indicated time points. IL-2 fusion protein concentrations were quantified using a customized electrochemiluminescence immunoassay. Additional sample processing and assay details are provided in Supplemental Methods.

### Cytokine blockade.

To evaluate pathways contributing to Fc-IL2Mut24–induced ISR, C57BL/6J or *Rag2^–/–^* mice were treated with Fc-IL2Mut24 together with blocking antibodies against IL-4, IL-5, IL-13, IFN-γ, IL-17A, or CD25, or with an isotype control. Antibodies were administered weekly beginning with the first Fc-IL2Mut24 dose. ISR development was monitored longitudinally.

### ILC expansion and adoptive transfer.

ILC2s were expanded from IL-33–treated *Rag2^–/–^* donor mice, whereas ILC1s and NK cells were isolated from *Rag2^–/–^* mice treated with IL-12 plus IL-15. Purified cells were transferred s.c. and/or intradermally into *Rag2^–/–^γc^–/–^* recipients, which were subsequently treated with Fc-IL2Mut24. ISR incidence and severity were assessed longitudinally. Detailed expansion, purification, and transfer procedures are provided in Supplemental Methods.

### Treg depletion.

*Rag2^–/–^* mice received T cells isolated from Foxp3-DTR-mCherry mice and were subsequently treated with IgG-IL2Mut24. Following ISR development, Tregs were depleted by administration of DT, and ISR severity and body weight were monitored longitudinally. Generation and validation of the Foxp3-DTR-mCherry mouse strain are described in Supplemental Methods.

### Tissue processing and flow cytometry.

Single-cell suspensions were prepared from spleen, lymph nodes, lung, visceral adipose tissue, CNS, and skin using tissue-specific mechanical and/or enzymatic dissociation. Cells were stained with viability, surface, and intracellular markers and analyzed by flow cytometry. Absolute cell numbers were determined using counting beads. Data were acquired on FACSymphony A3 Cell Analyzer (BD) and analyzed using FlowJo (Water Biosciences). Representative gating strategies are shown in Supplemental Figure 15, and antibodies are listed in Supplemental Table 1. Detailed tissue processing and staining procedures are provided in Supplemental Methods.

### scRNA-seq.

Skin leukocytes were enriched and multiplexed using oligonucleotide-conjugated antibodies before flow-cytometric sorting. Sorted populations were processed using the 10x Genomics Chromium Single Cell 5′ platform and sequenced on an Illumina NovaSeq X. Reads were processed using Cell Ranger and analyzed in R using Seurat. Low-quality cells were removed before normalization, dimensionality reduction, clustering, and cell-type annotation based on canonical markers.

Pseudo-bulk analyses were performed using DESeq2, and differential-state analyses were performed using muscat. Differentially regulated genes were identified using the criteria described in Supplemental Methods. Cell-type signatures and differential expression results are provided in Supplemental Tables 2 and 3.

### Cytokine measurements.

Serum and skin lysates were collected from treated mice, and cytokine concentrations were measured using V-PLEX Proinflammatory Panel 1 Mouse Kit (Meso Scale Discovery [MSD], Cat#K15048D). IL-13 was additionally quantified by ELISA. Detailed sample preparation and assay conditions are provided in Supplemental Methods.

### Histopathology.

Full-thickness injection site skin was formalin-fixed, paraffin-embedded, sectioned, and stained with H&E. Sections were evaluated by a board-certified veterinary pathologist using predefined criteria for epidermal, dermal, pannicular, and s.c. changes. Composite pathology scores were generated from individual tissue-layer severity scores. Detailed grading criteria are provided in Supplemental Methods.

### Generation and characterization of IL-2 molecules.

Mouse IL2WT and IL2Mut24 fusion proteins were generated as previously described (17). IL2Mut24 contained N103R and V106D substitutions in addition to manufacturability substitutions shared with IL2WT. Recombinant proteins were expressed in CHO cells, purified by protein A chromatography followed by size-exclusion chromatography, and characterized for purity, molecular mass, and endotoxin content. Additional construct and analytical details are provided in Supplemental Methods.

### IL-2 bioactivity assays.

IL-2 bioactivity was assessed using IL-2–dependent CTLL-2 cells and purified mouse ILC2s. Cells were stimulated with serial dilutions of IL-2 constructs, and proliferation was quantified by [^3^H]-thymidine incorporation. IL-2–induced CD25 upregulation on ILC2s was measured by flow cytometry. Additional purification and assay details are provided in Supplemental Methods.

### EAE.

EAE was induced in female C57BL/6 mice by immunization with MOG_35–55_ in complete Freund’s adjuvant followed by pertussis toxin. Mice were monitored daily for body weight and neurological disease using a 0–5 clinical scoring system. Detailed immunization and scoring procedures are provided in Supplemental Methods.

### Cynomolgus monkey studies.

In study 118075, cynomolgus monkeys received s.c. aldesleukin or AMG592 according to the schedules indicated in Figure 5A. Peripheral blood was collected longitudinally for immunophenotyping and serum cytokine analysis.

In study 118065, male and female cynomolgus monkeys received AMG592 at 10, 30, or 100 μg/kg s.c. once weekly for 3 months. Injection sites were collected at scheduled necropsy for histopathologic evaluation. Additional study design, sampling, and assay details are provided in Supplemental Methods.

### Statistics.

All data were analyzed using GraphPad Prism. For multiple group comparisons (e.g., flow cytometry data), 1-way ANOVA followed by Šídák’s multiple comparisons post hoc test was applied. For multiple-group comparisons over time (e.g., weight over time, ISR score over time), 2-way ANOVA (or mixed-effects analysis in case of missing values) followed by Šídák’s multiple comparisons post hoc test was applied. For nonparametric longitudinal EAE clinical scores, a mixed-effects model with Tukey’s multiple comparisons post hoc test was applied. *P* values of less than 0.05 were considered significant. All error bars represent the SEM unless otherwise noted.

### Study approval.

All mouse studies were approved by the IACUC of Amgen Inc. or the Charles River Accelerator and Development Lab (Charles River Laboratories, South San Francisco, California, USA). All studies involving cynomolgus monkeys were approved by the IACUC of Charles River Laboratories (Reno, Nevada, USA) (study 118065) or MPI Research (Mattawan, Michigan, USA) (study 118075). All studies were conducted in accordance with the US Animal Welfare Act and the *Guide for the Care and Use of Laboratory Animals* (National Academies Press, 2011).

### Data availability.

Values for all data points in graphs are reported in the Supporting Data Values file. All data and code needed to evaluate and reproduce the results in the paper are present in the paper and/or the Supplemental Materials. Materials will be made available to the scientific community by completion of a material transfer agreement. Requests can be made at www.amgen.com/partners/academic-collaborations/new-requests The scRNA-seq data generated in this study are available through GEO under accession GSE316048.

## Manuscript information

### Author contributions.

WD, AS, CM, and MT conceptualized the study and designed the experiments. AS, CM, MT, AV, SL, RP, and SM performed the experiments. YJ and XL performed RNA-seq analysis. SW and HST designed the tool molecules and provided structural modeling. RRH provided pathology reviews. MF was the study director of nonhuman primate studies. WD, AS, CM, and MT wrote the manuscript with input from all authors.

### AI disclosure.

ChatGPT (OpenAI, GPT5.6) was used for language editing and organization during manuscript preparation (from March to September 2026). All AI-assisted content was reviewed and verified by the authors, who take responsibility for the final manuscript.

### Conflict of interest.

All authors are current or former employees of Amgen Inc. and own stock in Amgen Inc.

### Funding.

This study was supported by the following:

- Amgen Inc.

## Supplementary Material

Supplemental data

Supporting data values

## Figures and Tables

**Figure 1:**
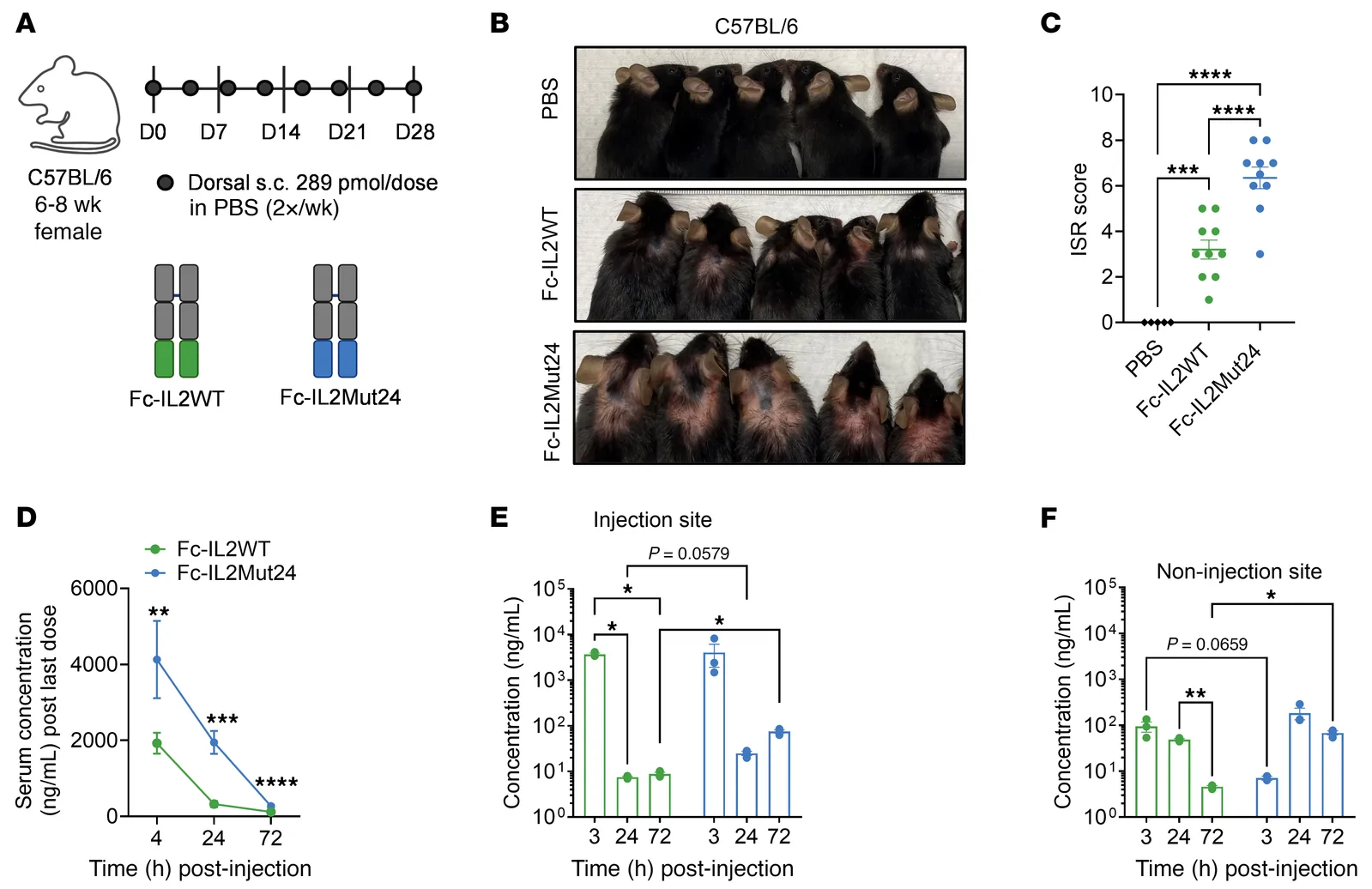
Fc-IL2Mut24 induces severe ISRs associated with prolonged systemic and cutaneous exposure. Six- to 8-week-old female C57BL/6 mice received dorsal s.c. dosing of Fc-IL2WT or Fc-IL2Mut24 289 pmol (~25 μg/dose/mouse in PBS) or PBS alone twice per week for 4 weeks. (**A**) Schematic representation of experimental design. (**B**) Representative photograph of dorsal ISR (*n* = 5 mice per group). (**C**) ISR score (*n* =5 mice in PBS, *n* =10 mice each in Fc-IL2WT and Fc-IL2Mut24). (**D**) Fc-IL2WT and Fc-IL2Mut24 plasma concentrations (ng/mL) at 3, 24, and 72 hours after a single dose, as detected by MSD (25 μg/dose, *n* =5 mice per condition at 3 and 24 hours, and *n* =10 mice per condition at 72 hours). (**E** and **F**) Concentrations (ng/mL) of Fc-IL2WT and Fc-IL2Mut24 detected from injection site (**E**) and noninjection site (**F**) skin samples at 3, 24, and 72 hours after a single dose, as detected by MSD (*n* =3 mice per time point and per condition). (**C**–**F**) Data shown as mean ± SEM. Two-tailed ordinary 1-way ANOVA followed by Šídák’s post hoc test. *P < 0.05, **P < 0.01, ***P < 0.001, ****P < 0.0001.

**Figure 2:**
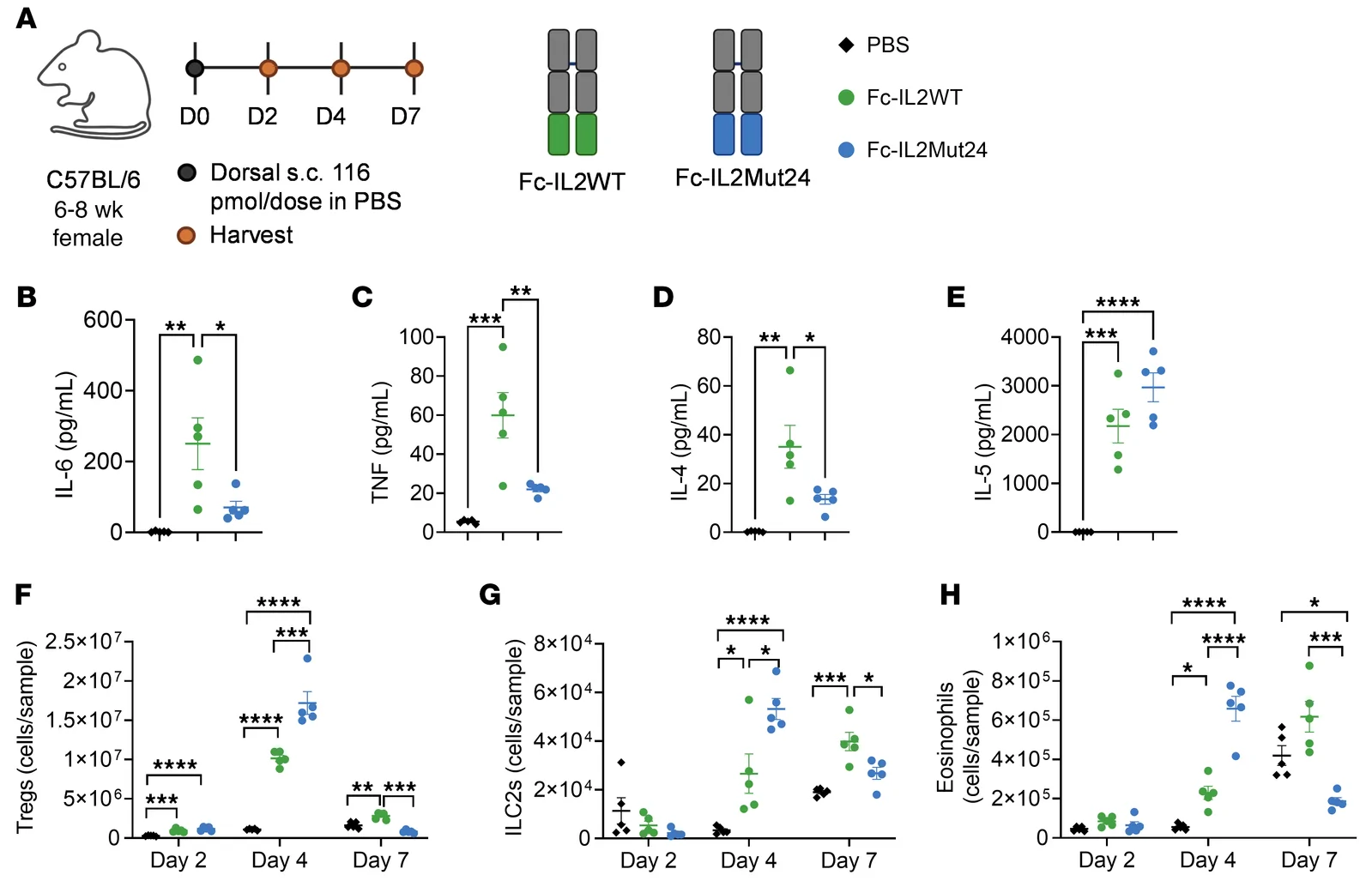
Fc-IL2Mut24 induces Treg expansion but concomitantly amplifies ILC2 and eosinophil responses. Six- to 8-week-old female C57BL/6 mice received dorsal s.c. dosing of Fc-IL2WT, Fc-IL2Mut24 at 116 pmol (~10 μg/dose/ mouse), or PBS alone on day 0. Mice were euthanized on days 2, 4, and 7 for serum cytokine analysis by MSD and splenic cell analysis by flow cytometry. (**A**) Schematic representation of experimental design. (**B**–**E**) Concentrations (pg/mL) of IL-6 (**B**), TNF (**C**), IL-4 (**D**), and IL-5 (**E**) in serum on day 2. (**F**–**H**) Treg (**F**), ILC2 (**G**), and eosinophil (**H**) counts (cells per sample) in spleen on days 2, 4, and 7. Data are representative of 3 independent experiments. Data shown as mean ± SEM from *n* = 5 mice per condition and per time point. Two-tailed ordinary 1-way ANOVA followed by Šídák’s post hoc test. *P < 0.05, **P < 0.01, ***P < 0.001, ****P < 0.0001.

**Figure 3:**
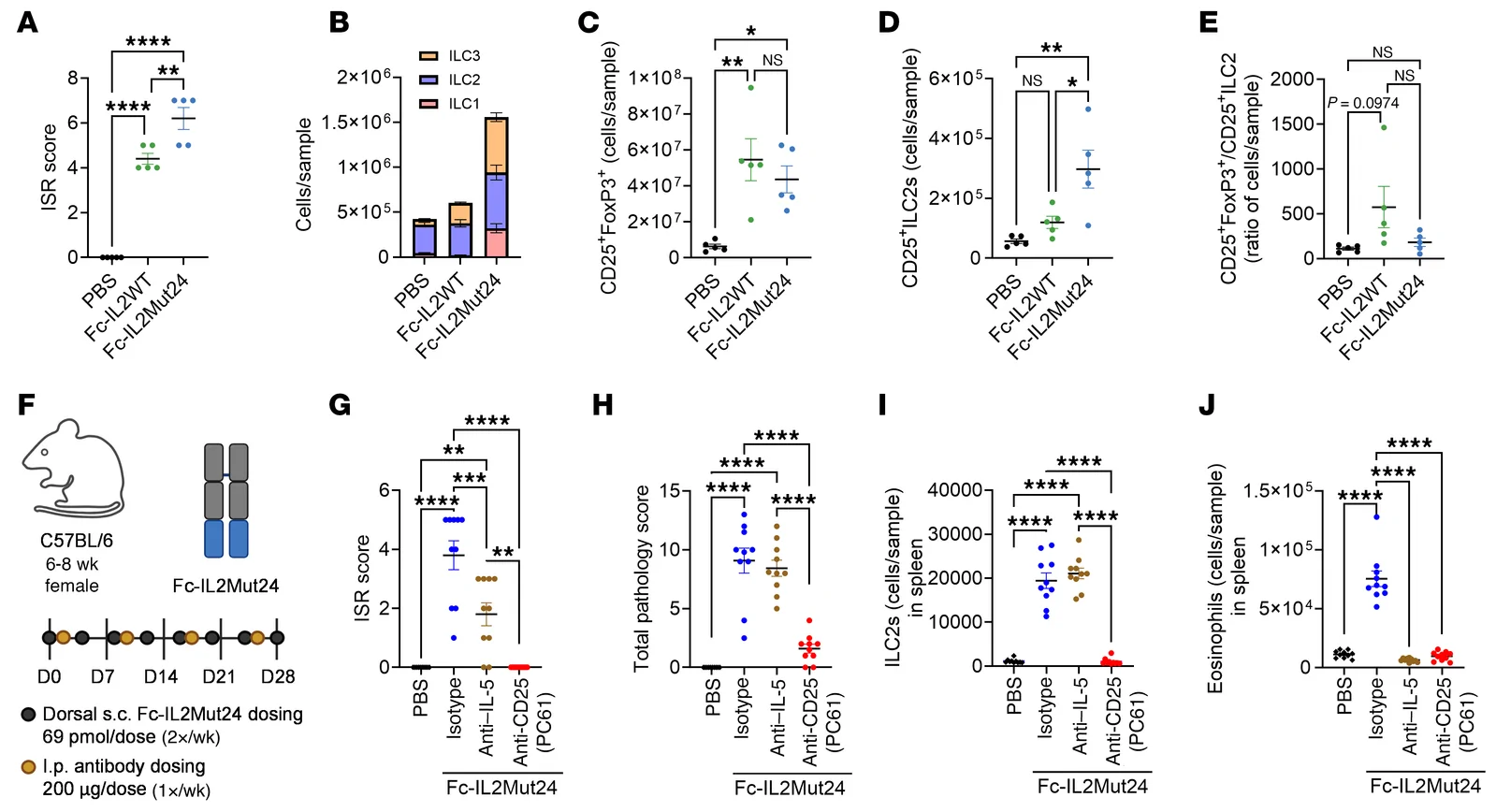
Fc-IL2Mut24 induces a CD25-dependent type 2 inflammatory program in skin. (**A**–**E**) C57BL/6 mice (*n* = 5 mice per group) received dorsal s.c. dosing twice weekly for 4 weeks with PBS, 69 pmol (~6.5 μg/dose/mouse) Fc-IL2WT, or Fc-IL2Mut24. (**A**) ISR scores at endpoint. (**B**) ILC1, ILC2, and ILC3 cell counts per sample in skin at endpoint represented as stacked bar graph, as analyzed by flow cytometry. (**C** and **D**) CD25^+^FoxP3^+^ cells (**C**) and CD25^+^ILC2s (**D**) in skin at endpoint, shown as cell counts per sample, as analyzed by flow cytometry, and ratio thereof (**E**). (**F**–**J**) C57BL/6 mice (*n* = 10 mice per group) received dorsal s.c. dosing twice weekly for 4 weeks with 69 pmol (~6.5 μg/dose/mouse) Fc-IL2Mut24 and were cotreated once weekly with 200 μg anti–IL-5, anti-CD25 or isotype control. (**F**) Schematic representation of experimental design. (**G**) ISR scores at endpoint. (**H**) Skin pathology scores at endpoint. (**I** and **J**) ILC2 counts (cells/sample, **I**) and eosinophil counts (cells/sample, **J**) in spleen as analyzed by flow cytometry. Data shown as mean ± SEM. Two-tailed ordinary 1-way ANOVA followed by Šídák’s multiple comparisons post hoc; only selected comparisons are shown. *P < 0.05, **P < 0.01, ***P < 0.001, ****P < 0.0001.

**Figure 4:**
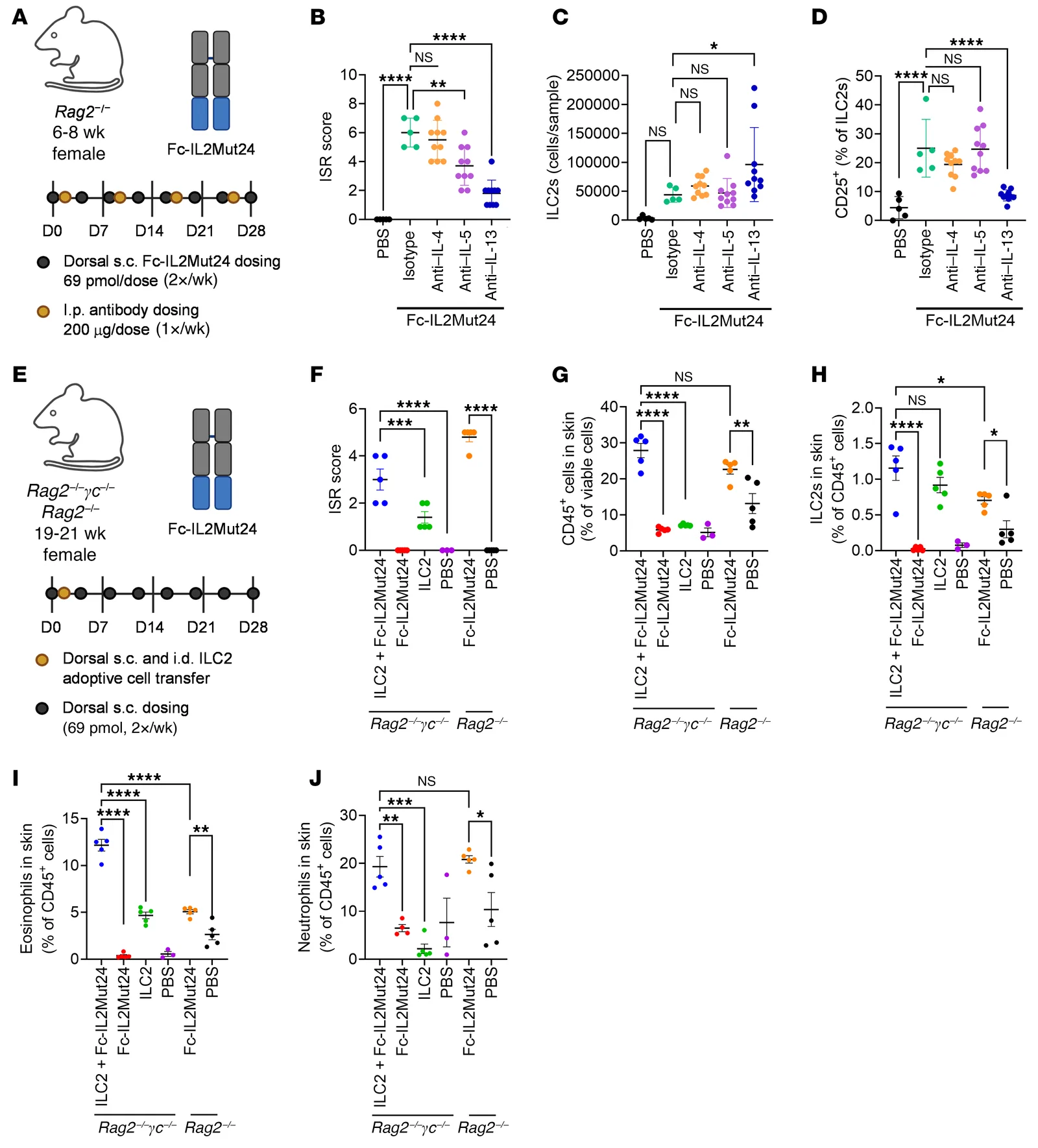
ILC2s are sufficient for IL-2–induced cutaneous inflammation. (**A**–**D**) *Rag2^–/–^* mice (*n* = 5 mice in PBS and isotype and *n* = 10 mice in all other groups) received dorsal s.c. dosing twice weekly for 4 weeks with 69 pmol (~6.5 μg/dose/mouse) Fc-IL2Mut24 and were cotreated once weekly with 200 μg anti–IL-4, anti–IL-5, anti–IL-13 or isotype control. (**A**) Schematic representation of experimental design. (**B**) ISR scores at endpoint. (**C**) ILC2 counts (absolute number of cells per sample) and (**D**) frequency of CD25^+^ cells among ILC2s in skin, as analyzed by flow cytometry. (**E**–**J**) *RAG2^–/–^γc^–/–^* mice (*n* = 3 mice in PBS groups, *n* = 5 mice per group in all other groups) received both s.c. and i.d. adoptive transfer of 2 × 10^6^ ILC2 cells and were then treated s.c. twice weekly for 4 weeks with 69 pmol Fc-IL2Mut24. *RAG2^–/–^* mice (*n* = 5) were treated in parallel as a control. (**E**) Schematic representation of experimental design. (**F**) ISR scores at endpoint. (**G**) Frequency of CD45^+^ among live cells, (**H**–**J**) Frequency of ILC2s (**H**), eosinophils (**I**), and neutrophils (**J**) in CD45^+^ cells in skin at endpoint analyzed by flow cytometry. Data are representative of 2 independent experiments. Data shown as mean ± SEM. Two-tailed ordinary 1-way ANOVA followed by Šídák’s multiple comparisons post hoc; only selected comparisons are shown. *P < 0.05, **P < 0.01, ***P < 0.001, ****P < 0.0001.

**Figure 5:**
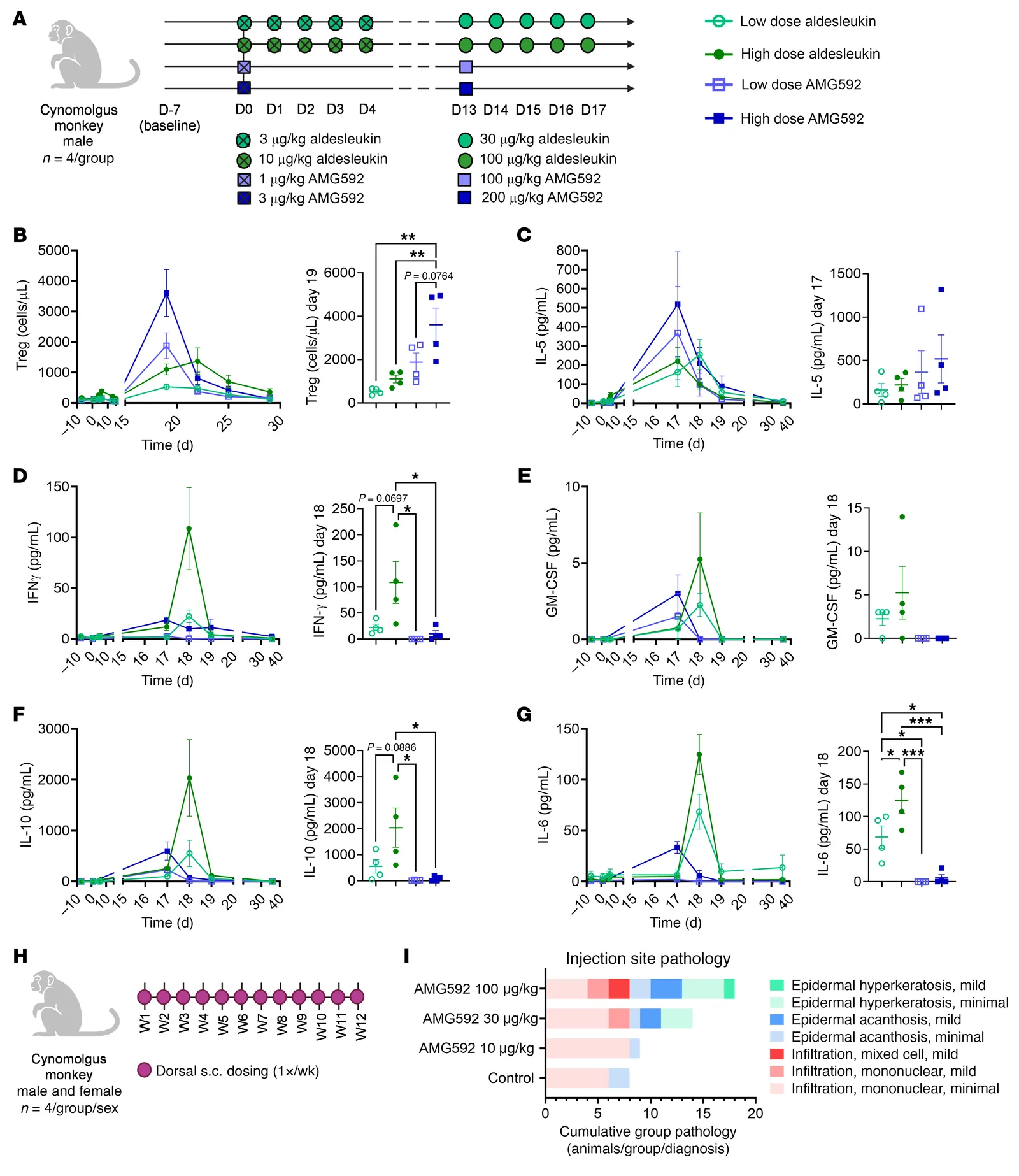
AMG592 expands Tregs but induces IL-5 production and injection site pathology in cynomolgus monkeys. (**A**–**G**) Male cynomolgus monkeys (*n* = 4 per group) received s.c. dosing of low or high doses of aldesleukin (3 or 10 μg/kg on days 0–4 followed by 30 or 100 μg/kg on days 13 –17) or AMG592 (1 or 3 μg/kg on day 0 followed by 100 or 200 μg/kg on day 13). Blood samples were collected at the indicated time points for PD analysis. (**A**) Schematic representation of study 118075. (**B**) Circulating Tregs (gated as CD3^+^CD4^+^FoxP3^+^CD25^+^ cells prior to dosing and CD3^+^CD4^+^FoxP3^+^ cells after dosing), as assessed by flow cytometry. (**C**–**G**) Serum cytokine concentrations following treatment: IL-5 (**C**), IFN-γ (**D**), GM-CSF (**E**), IL-10 (**F**), and IL-6 (**G**). Peak values (day 19 in **B**, day 17 in **C**, day 18 in **D**–**G**) are shown in right panels. Data shown as mean ± SEM. Two-tailed ordinary 1-way ANOVA followed by Šídák’s multiple comparisons post hoc; only selected comparisons are shown. *P < 0.05, **P < 0.01, ***P < 0.001. (**H** and **I**) Cynomolgus monkeys (*n* = 4 male and 4 female per group) received s.c. AMG592 (100 μg/kg, 30 μg/kg or 10 μg/kg) or vehicle control once per week for 12 weeks. Injection site skin was collected on day 92 after the last dose. (**H**) Schematic representation of study 118065. (**I**) Cumulative histopathology at the dorsal s.c. injection site. Bars represent the total number of animals per group exhibiting each lesion category and severity. Observed changes included epidermal hyperkeratosis and acanthosis (minimal or mild) as well as dermal inflammatory infiltrates (mononuclear or mixed-cell, minimal or mild).

**Figure 6:**
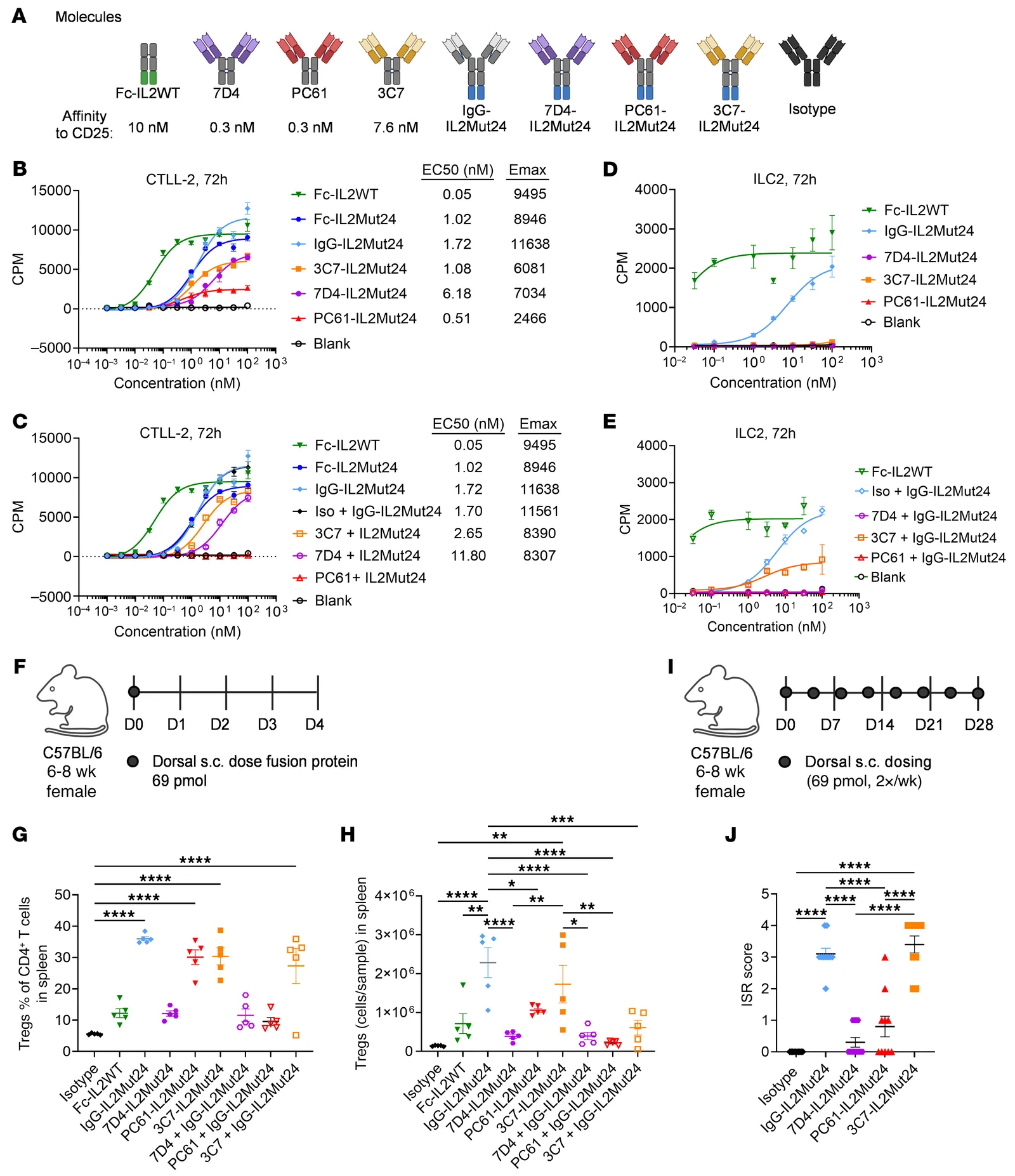
Epitope-specific CD25 engagement tunes IL-2 signaling strength and tissue immune responses. (**A**–**E**) Proliferation assays using CTLL-2 cells (**B** and **C**) or purified ILC2s (**D** and **E**) treated with serial dilutions of IL-2 fusion proteins or controls. (**A**) Schematic of Fc-IL2WT and anti-CD25–IL2Mut24 fusion proteins; indicated values denote binding affinities for mouse CD25. (**B** and **D**) Proliferative responses to 7D4-IL2Mut24, PC61-IL2Mut24, 3C7-IL2Mut24, Fc-IL2WT, Fc-IL2Mut24, and IgG-IL2Mut24. (**C** and **E**) Responses to equimolar mixtures of unconjugated anti-CD25 antibodies with IgG-IL2Mut24. (**B** and **C**) Data are shown as mean ± SEM of 3 technical replicates from 1 representative experiment of 3. (**D** and **E**) Data are from 1 experiment. (**F**–**H**) C57BL/6J mice (*n* = 5/group) received 69 pmol of the indicated fusion proteins or matched combinations of unconjugated anti-CD25 antibodies with IgG-IL2Mut24 and were analyzed on day 4. (**F**) Schematic representation of experimental design. (**G** and **H**) Frequency (**G**) and cell counts per sample (**H**) of CD25^+^FOXP3^+^ Tregs within the CD4^+^ T cell compartment in spleen, as analyzed by flow cytometry. (**I** and **J**) C57BL/6J mice were treated s.c. twice per week for 4 weeks with 69 pmol of indicated protein (*n* = 10 mice per group). (**I**) Schematic representation of experimental design. (**J**) ISR scores at endpoint. Data shown as mean ± SEM. Two-tailed ordinary 1-way ANOVA followed by Šídák’s multiple comparisons post hoc test, only selected comparisons are shown. *P < 0.05, **P < 0.01, ***P < 0.001, ****P < 0.0001.

**Figure 7:**
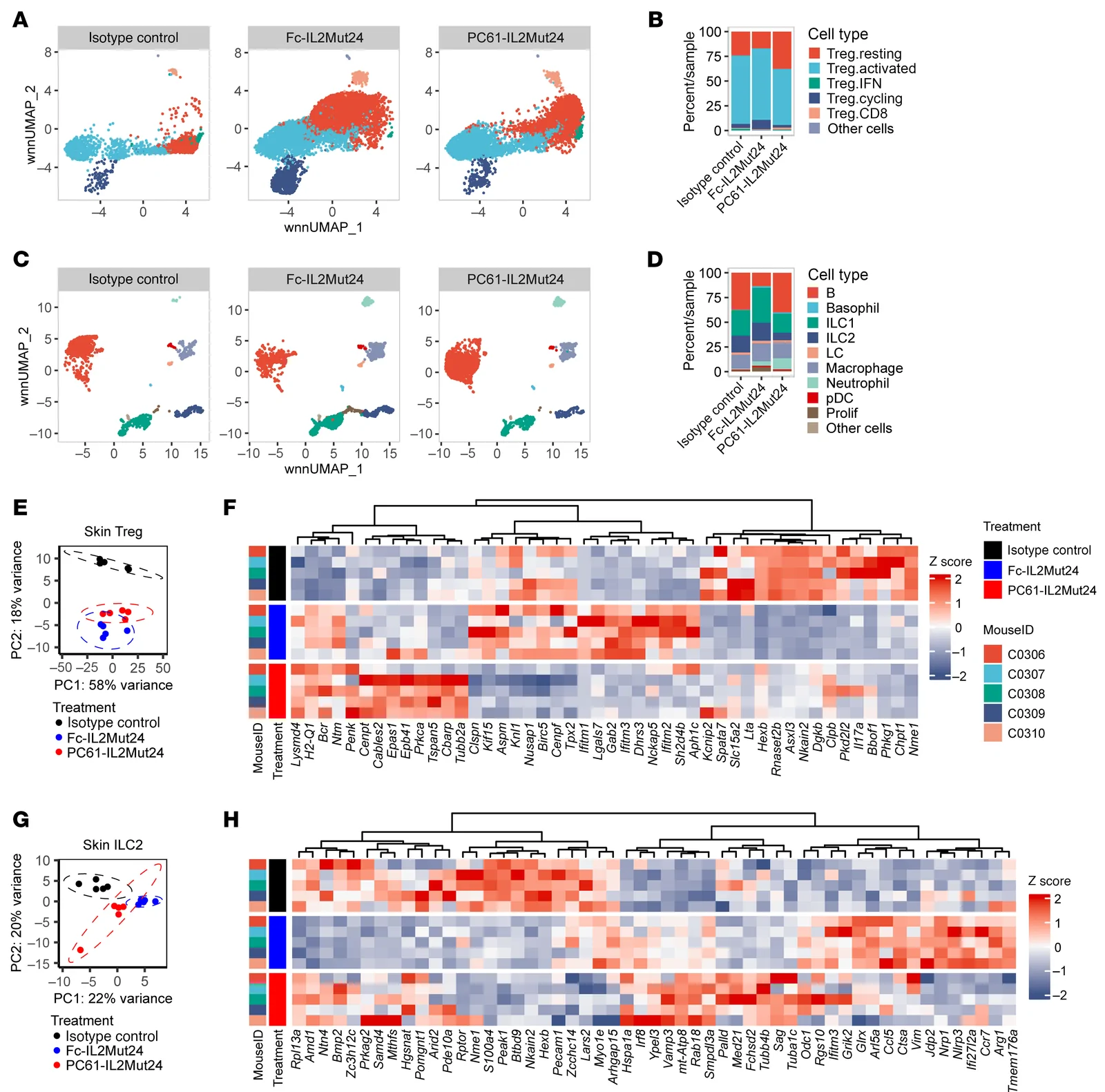
Fc-IL2Mut24 and PC61-IL2Mut24 differentially remodel the cutaneous immune transcriptional landscape. BALB/c-FoxP3-GFP reporter mice received a single dorsal s.c. dose of isotype control, Fc-IL2Mut24, or PC61-IL2Mut24 (69 pmol). Skin samples were collected and processed on day 4 after dosing. Uniform manifold approximation and projection (UMAP) of Treg subsets from the CD45^+^TCRβ^+^GFP^+^ sorted samples (**A**) and CD45^+^TCRβ^–^ GFP^–^ immune cell populations (**C**) in skin. Average proportion of different Treg subsets from the CD45^+^TCRβ^+^GFP^+^ sorted samples (**B**) and different immune cell subsets from the CD45^+^TCRβ^–^ GFP^–^ sorted samples in each condition (**D**) in skin. Principal component analysis (PCA) of genes expressed by Tregs (**E**) and ILC2s (**G**) with 95% confidence intervals marked by dashed circles. Heatmap of top 50 genes differentially expressed by Tregs (**F**) and ILC2s (**H**) across isotype, Fc-IL2Mut24, and PC61-IL2Mut24 treatment groups in skin.

**Figure 8:**
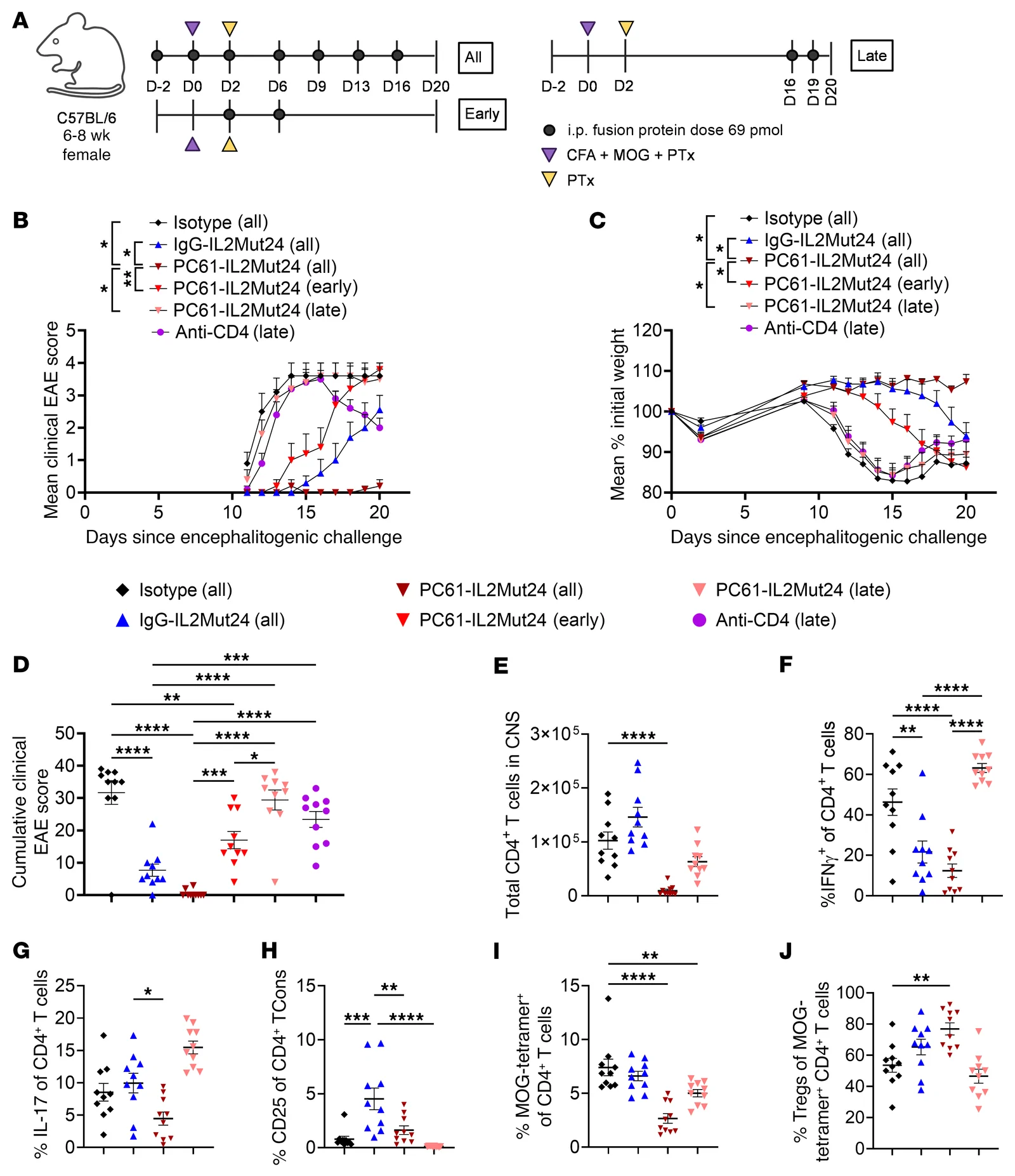
PC61-IL2Mut24 outperforms IgG-IL2Mut24 in suppression of EAE onset. C57BL/6J mice (*n* = 10) were treated i.p. with 100 μL PBS containing isotype, PC61-IL2Mut24, IgG-IL2Mut24, or anti-CD4 on schedules: “All” (days –2, 0, 2, 6, 9, 13, and 16), “Early” (days 2 and 6), or “Late” (days 16 and 19). EAE was induced on day 0 using 200 μg MOG_35–55_ emulsified in CFA, and 120 ng pertussis toxin was administered i.p. on days 0 and 2. (**A**) Schematic representation of experimental design. (**B**) Clinical EAE scores over time (days). (**C**) Body weight over time (days) as percentage change from initial weight. (**D**) Cumulative EAE scores over time (days). (**E**–**J**) At study endpoint, CNS-infiltrating cells were isolated and stained with MOG_35–55_-IA^b^ tetramer. Total CD4^+^ T cell counts (**E**), frequency of IFN-γ^+^ (**F**), IL-17A^+^ (**G**), CD25^+^ (**H**), and MOG-tetramer^+^ (**I**) cells among CD4^+^ cells, frequency of FoxP3^+^ cells among MOG-tetramer^+^ CD4^+^ T cells (**J**) in the CNS, as analyzed by flow cytometry. Data are shown as mean ± SEM from *n* = 10 mice per group. Data are representative of 2 independent experiments. (**B** and **C**) EAE scores and body weights were analyzed using mixed-effects models with Tukey’s multiple-comparisons test. PC61-IL2Mut24 (All) reduced EAE scores versus isotype on days 5–13 (*), IgG-IL2Mut24 on days 11–13 (*), PC61-IL2Mut24 (Early) on days 10–13 (**), and PC61-IL2Mut24 (Late) on days 5–13 (*). Body weight was greater versus isotype on days 3–13 (*), IgG-IL2Mut24 on day 13 (*), PC61-IL2Mut24 (Early) on days 10–13 (*), and PC61-IL2Mut24 (Late) on day 3 and days 5–13 (*). *P < 0.05; **P < 0.01. (**D**–**J**) Two-tailed ordinary 1-way ANOVA followed by Šídák’s post hoc test. *P < 0.05, **P < 0.01, ***P < 0.001, ****P < 0.0001.

## Version 1 09/29/2026

In-Press Preview

## Version 2 11/16/2026

Electronic publication
