# Supplementary material for: Receptor-tuned IL-2 mutein decouples Treg expansion from ILC2-driven cutaneous inflammation
Source: J Clin Invest. 2026 Sep 29;136(22):e207126. doi: 10.1172/JCI207126 (PMC13767618; doi:10.1172/JCI207126)
Supplement: Supplementary file 1 [file jci-136-207126-s210.pdf]

SUPPLEMENTAL METHODS

Mice

Female (6-12 weeks old) C57BL/6NCrl and BALB/cAnNCrl mice were purchased from Charles River Laboratories; B6.129S6-Rag2^{tm1Fwa} N12， C57BL/6NTac.Cg-*Rag2*^{tm1Fwa} *Il2rg*^{tm1Wjl} mice and C57BL/6NTac were obtained from Taconic Biosciences; and B6.Cg-Foxp3tm2Tch/J, C.Cg-Foxp3tm2Tch/J, C3H/HeJ, and C57BL/6J mice were purchased from The Jackson Laboratory. All mice were housed under specific pathogen-free (SPF) conditions at CRADL. Animals were maintained in individually ventilated cages under controlled environmental conditions (temperature: 22 ± 2°C, relative humidity: 50-60%) on a 12-hour light/dark cycle. Mice had ad libitum access to autoclaved food and water and were acclimated for at least five days prior to experimentation.

In Vivo Treatments and Interventions

Mice received either high (25 μg/289 pmol), optimal (10 μg/116 pmol), low (6.5 μg/ 69 pmol) or ultra-low dose (2.5 μg/28.9 pmol) of IL-2 molecules as specified in figure legends. For single dose pharmacodynamic (PD) studies, animals received (10 μg/116 pmol) per mouse of either Fc-IL2WT or Fc-IL2Mut24 via s.c. injection. Control mice were given an equal volume of vehicle (PBS). For the preclinical model of injection-site reaction (ISR), mice were dosed subcutaneously twice weekly for a total of eight doses with either high (25 μg/289 pmol) or low (6.5 μg/ 69 pmol) dose of either Fc-IL2WT or Fc-IL2Mut24， unless specified otherwise in figure legends. Development of lesions at the injection site was observed around the 4th-5th dose. Mice were monitored twice per week for body weight changes and ISRs symptoms.

Pharmacokinetics (PK) and Tissue Biodistribution

C57BL/6J mice were administered a single high dose (289 pmol) of Fc-IL2WT or IL2Mut24 fusion proteins by subcutaneous injection over the shoulder region. Blood samples were collected at 4, 24, and 72 hours post-injection into SARSTEDT Microvette® K3 EDTA plasma separation tubes. Plasma was isolated by centrifugation and stored at −70 ± 10 °C until analysis.

Skin samples from injection site and non-injection site from lower back were excised, weighed and transferred into bead-containing Precellys® soft tissue homogenizing CK14 tubes (Bertin Instruments). Lysis buffer (50 mM Tris-HCl, 100 mM NaCl, 0.1% Triton X-100, pH 7.4) supplemented with cOmplete™ Mini EDTA-free protease inhibitor tablets (Roche; cat# 11836170001) was added at a ratio of 200 mg tissue per mL buffer. Tissues were homogenized using a Precellys® homogenizer with one cycle consisting of three 30-second homogenization steps separated by 15-second rest intervals. Samples were maintained on ice immediately before and after homogenization to preserve protein integrity.

IL-2 fusion protein concentrations in plasma and skin homogenates were quantified using a customized electroluminescence immunoassay on a MESO SECTOR S600 instrument (Meso Scale Discovery). In short, biotinylated anti-mouse IL-2 antibody (R&D Systems; BAF402) was used as the capture reagent, and ruthenylated goat anti-mouse IgG antibodies (Jackson ImmunoResearch; Cat#115-005-205 and 115-005-206) were used for detection. Analyte concentrations were interpolated from standard curves using Watson LIMS™ Non-GLP Prod. Data acquisition and analysis were performed using Discovery Workbench 4.0 software (MSD).

Injection Site Reaction Scoring

All mice were given a physical examination of the injection site once per week. The scoring system was modified from ulcerative dermatitis (49), atopic dermatitis (50) and psoriasis (51) scoring. Briefly, the size of the affected area at the injection site, including visible hair-coat loss, was evaluated by measuring the maximal continuous length of the largest lesion in one dimension only. The lesion size was scored as follows: 1, <1 cm; 2, 1-2 cm; 3, 2-3 cm; and 4, greater than 3 cm. The bleeding characteristics were identified as 1, hemorrhage only; 2, slight active bleeding; 3, hemorrhage and moderate to severe active bleeding (>2 mm). Skin thickness was evaluated by skin desquamation and induration: 1 mild, minor dry spot with minimal skin change; 2, moderate, flaking dry spots with visible skin thickness; 3 severe, flaking across a large surface area with notable thickness and tight ridges. Inflammation was assessed based on tissue edema and erythema and scored as follows: 1, inflamed eyelid or skin; 2, inflamed eyelid and skin; and 3, involvement of additional tissues, such as the head region. When inflammation involved the head region, swollen eyelids, excessive ocular discharge, and possible crusting were also recorded. The overall injection-site severity index was calculated as the sum of the individual component scores.

In vivo antibody blockade during IL2Mut24-induced injection site reactions

For cytokine-blockade studies, *Rag2*$^{-/-}$ mice received 69 pmol Fc-IL2Mut24 by dorsal subcutaneous injection twice weekly for four weeks. Mice were co-treated by intraperitoneal injection with 200 μg/mouse of anti-IL-4 (clone 11B11; BioXCell, BE0045), anti-IL-5 (clone TRFK5; BioXCell, BE0198), anti-IL-13 (clone 8H8; InvivoGen, mil13-mab9-1), anti-IFNγ (clone XMG1.2; BioXCell, BE0055), anti-IL-17A (clone 17F3;

BioXCell, BE0173), anti-CD25 (clone PC-61.5.3; InvivoGen, mcd25c1-mab10-1), or rat IgG1κ anti-horseradish peroxidase isotype control (clone HRPN; BioXCell, BE0088). Antibodies were administered once weekly beginning on day 0, concurrently with the first Fc-IL2Mut24 dose. Injection site reactions were monitored throughout the study.

ILC2, ILC1, and NK cell expansion and adoptive transfer to *Rag2*$^{-/-}$*γc*$^{-/-}$ Mice

For ILC2 expansion, female *Rag2*$^{-/-}$ donor mice were injected intraperitoneally with 4 μg recombinant human IL-33 (BioLegend, Cat. 581808) in 100 μL PBS on days 0, 2, 4, and 6. On day 8, lungs were harvested and digested for 1 hour at 37°C with Liberase TL (Roche, Cat. 05401020001; 250 μg/mL) and DNase I (Sigma-Aldrich, Cat. D4263-1VL; 1 μg/mL). Digests were filtered through 70-μm strainers, treated with ACK lysis buffer (Gibco, Cat. A10492-01) and enriched using the Mouse ILC2 Isolation Kit (STEMCELL Technologies, Cat. 19842). Purified ILC2s were expanded for 3 days at 0.5 × 10^6 cells/mL in complete RPMI containing recombinant human IL-2 (20 ng/mL; PeproTech, Cat. 200-02).

For ILC1 and NK-cell expansion, female *Rag2*$^{-/-}$ donor mice received 6 μg recombinant mouse IL-15 (PeproTech, Cat. 210-15) and 1 μg recombinant mouse IL-12 (PeproTech, Cat. 210-12) intraperitoneally every other day for five doses. One day after the final dose, livers were harvested and digested for 30 minutes at 37°C with Liberase TL (250 μg/mL) and DNase I (1 μg/mL). Leukocytes were enriched using a 30%/70% Percoll gradient, and ILC1s and NK cells were purified by flow-cytometric sorting.

Rag2$^{-/-}$*γc*$^{-/-}$ recipients received either 2 × 10^6 ILC2s (Figure 4) in the initial ILC2 reconstitution experiment or 3 × 10^5 ILC2s, ILC1s, or NK cells (Supplemental Figure 9) in

the matched lineage-comparison experiment. The lower cell number was used in the latter experiment because sufficient numbers of ILC1s and NK cells could not be obtained with the isolation and expansion protocol to support transfer of 2 × 10^6 cells per recipient.

Treg depletion and assessment of IL2Mut24-induced toxicity

Female *Rag2*$^{-/-}$ mice (6-8 weeks old) received 1 × 10^7 T cells isolated from the spleens of Foxp3-DTR-mCherry knock-in mice (Mouse T Cell Isolation Kit, STEMCELL, cat#19851) by retro-orbital injection in 50 μL PBS. Mice were subsequently administered 69 pmol IgG-IL2Mut24 subcutaneously in 100 μL PBS twice weekly for three weeks. Following the final IgG-IL2Mut24 dose and after ISR onset, Tregs were depleted by intraperitoneal administration of unnicked diphtheria toxin from *Corynebacterium diphtheriae* (lyophilized powder; Sigma-Aldrich/SAFC) at 500 ng/mouse in 100 μL PBS on days 21, 24, 28, 36, and 51. ISR severity and body weight were monitored longitudinally.

Foxp3-DTR-mCherry mice

Foxp3-DTR-mCherry knock-in mice were generated on a C57BL/6J background by Biocytogen using CRISPR/Cas9-mediated insertion of an IRES-DTR-1×G4S-mCherry cassette between Foxp3 exon 14 and the 3′ untranslated region. The targeting design was based on Foxp3 transcript 204 (NM_001199347.1). Founder mice were crossed with C57BL/6J mice, and germline transmission was confirmed by PCR, sequencing, and Southern blotting. Functional validation confirmed specific co-expression of FOXP3 and mCherry. Intraperitoneal administration of diphtheria toxin at 100, 200, or 500 ng per mouse resulted in complete Treg depletion in the spleen and inguinal lymph nodes within

12 hours. Depletion was maintained for up to 72 hours, after which the Treg population began to reconstitute.

Tissue Processing and Flow Cytometry

The spleen, lung, visceral adipose tissue (VAT), lymph nodes (LN), central nervous system (CNS), and skin tissues were dissected from mice and placed into PBS (Gibco) containing 2% FBS. The lung, VAT and CNS tissues were cut into small pieces and incubated in media containing 2 mg/mL collagenase (Millipore Sigma, Cat#11088858001) and RNase-free DNase (Sigma-Aldrich, Cat# D4263-1VL) and incubated at 37°C for 30-60 min under agitation, filtered over a 70 μm cell strainer (Corning), and washed with wash buffer (PBS+ 1 mM EDTA + 2% FBS) to obtain a single-cell suspension. CNS was further enriched using Percoll gradient. CNS suspension was resuspended in 6 mL of 37% Percoll, overlaid on top of 6 mL of 70% Percoll, and centrifuged at 2,000 RPM for 20 min with no brake at 4°C. The interphase containing the enriched leukocytes was collected and used. For spleen and LN processing, the tissues were placed on 70 μm filter and gently smashed using the back of a syringe plunger. The splenocytes were then lysed with 2 mL of ACK lysis buffer (Gibco, Cat#A10492-01) for 5 min and neutralized with wash buffer, followed by another round of washing. For skin tissue processing, subcutaneous fat was scraped off using a scalpel, minced to a paste and digested in a buffer containing RPMI, 250 μg/ml Liberase TL Research Grade (Roche, Cat# 5401020001) and 1 μg/ml Deoxyribonuclease I from bovine pancreas (Sigma-Aldrich, Cat# D4263-1VL) per 2x2 cm of skin for 90 mins-2 hours at 37°C. The tissues were then strained on a 70 μm filter, pelleted at 4°C and washed once. The obtained single cell suspensions of tissues were then used for further downstream analysis. Cell Stimulation

Cocktail plus Protein Transport Inhibitors (eBioscience™, cat#00-4975-93) reagent was used for cell stimulation (for 4 hours, at 37°C). Cells were washed prior to staining. For flow cytometry staining, cells were preincubated for 10 min with anti-mouse CD16/32 (Biolegend, Cat# 101301) to block mouse Fc receptor and then surface stained with cocktails (50 μl) of fluorochrome-conjugated antibodies in Brilliant Stain Buffer (BD Biosciences, Cat# 563794) with 1:1,000 LIVE/DEAD™ Fixable Aqua Dead Cell Stain Kit (Invitrogen, Cat# L34957) for 20 min. For intracellular staining, samples were fixed and permeabilized and stained with Foxp3/Transcription Factor Staining Buffer Set (eBioscience, Cat#00-5523-00) per manufacturer's directions. To obtain cell counts, CountBright™ Plus Absolute Counting Beads (Invitrogen, Cat# C36950) were added to samples immediately before flow cytometric analysis. Samples were acquired on Symphony Cytometer (BD) using BD FACSDiva software and then analyzed using the FlowJo software. Representative gating strategies for flow cytometric analysis are summarized in Supplemental Figure 15. The list of flow cytometry antibodies used in this study can be found in Supplementary Table 1.

scRNAseq Analysis

For scRNA seq, skin cell suspensions were enriched with Percoll density gradient centrifugation (Cytiva, Cat# 17144003) gradient. Briefly, cells were resuspended in 6 mL of 37% Percoll, overlaid on top of 6 mL of 70% Percoll, and centrifuged at 800 x g for 20 min with no break at 4°C. The interphase containing the enriched leukocytes was collected for downstream processing.

Before staining, cells were preincubated for 20 min with anti-mouse CD16/32 (Biolegend, Cat# 101301). For sample multiplexing, each individual sample from mice

within the same treatment group was incubated with a cocktail of TotalSeq™-C Mouse Universal Cocktail, V1.0 (Biolegend, Cat#199903) plus one TotalSeq™-C Hashtag antibody (Biolegend, 2 μL/sample, Hashtags C0306 (Cat#155871), C0307 (Cat#155873), C0308 (Cat#155875), C0309 (Cat#155877), and C0310 (Cat#155879)) at 4°C for 15 min. Samples were washed with BD Pharmingen™ Stain Buffer (BD Biosciences, Cat#554657) and were stained for CD45-BV786 (Biolegend, Cat#103149), TCRβ-PE (BD Biosciences, Cat#561081) and LIVE/DEAD™ Fixable Near-IR Dead Cell Stain Kit (Invitrogen, Cat#L34976) at 4°C for 15 min before sorting. Within each treatment group, samples were pooled and sorted on a FACS Aria (BD Biosciences) into three fractions: $CD45^{+}TCR\beta^{+}$ GFP^{+}, $CD45^{+}TCR\beta^{+}GFP^{-}$, $CD45^{+}TCR\beta^{-}GFP^{-}$. Sorted cells were captured in droplet emulsions using Chromium X (10x Genomics) and libraries were constructed using the 10x Genomics Chromium Next GEM Single Cell 5' Kit v2 (10x Genomics, Cat#1000263) and Chromium Next GEM Single Cell 5' Gel Bead Kit v2 (10x Genomics, Cat# 1000264) according to manufacturer's instructions. Amplified 5' Gene Expression, V(D)J and Cell Surface Protein libraries were profiled using the 4200 TapeStation System (Agilent Technologies) and pooled at 10:1:1 ratio. Pooled samples were sequenced on Illumina NovaSeq X (UCSF, Center for Advanced Technology (CAT)) using a 28-10-10-90 asymmetric read configuration. A minimal sequencing depth of 50,000 reads/cell was targeted for each Gene Expression Library.

Raw data were aligned to the mouse reference genome mm10 and GRCm38 V(D)J using a custom workflow of Cell Ranger (v7.1.0) that supports sample demultiplexing and 5' immune profiling. The filtered output matrices were imported into R (4.1.0) for downstream analysis.

The Seurat R package (version 4.3.0) was used for further analysis of the single-cell data. Low-quality cells were removed by excluding those with ≥10% mitochondrial UMIs, ≤200 detected genes, or UMI counts outside the 2.5-97.5 percentile of the library-size distribution. RNA and ADT count matrices were normalized using the “LogNormalize” and “CLR” methods, respectively. The top 2000 variable genes were identified with “FindVariableFeatures.” Data was scaled (“ScaleData”) before PCA reduction (“RunPCA”), and graph-based clustering was performed via “FindMultiModalNeighbors” and ‘‘FindClusters’’ function at a resolution of 0.5, 0.8, 1.0 and 2.0. Dimensionality reduction was performed with ‘‘RunUMAP’’ function and visualized by Uniform Manifold Approximation and Projection (UMAP). Cell subclusters were annotated to known biological cell types using canonical marker genes (Supplemental Figure 13C-E). Gene signatures used to define cell populations are provided in Supplementary Table 2 (22).

For principal component analysis of ILC2, Tregs, $CD4^+$ and $CD8^+$ conventional T cells, the “AggregateExpression” function was used to sum together gene counts of all the cells from each treatment group for each cell type. Summed gene counts were extracted from the pseudo-bulk single-cell samples to perform “DESeq” analysis using the DESeq2 package (v1.34.0). PCA matrix was obtained by “plotPCA” after VST transformation, and the results were visualized by ggplot2 (v3.5.2).

Differential state analysis was performed using the muscat package (v1.0.1) to identify differentially regulated genes in each cell type between (1) Fc-IL2Mut24 and isotype control, (2) PC61-IL2Mut24 and isotype control and (3) PC61-IL2Mut24 and Fc-IL2Mut24 groups. Mitochondrial, ribosomal, histone, TCR and predicted genes were excluded from the analysis. The 120 most significantly up- or down-regulated genes

(adjusted local p-value < 0.05) were selected, duplicate genes were removed, and heatmaps were generated using the ComplexHeatmap (v2.6.2). A complete list of the up to top 120 significantly differentially regulated genes can be found in Supplementary Table 3.

Cytokine Measurements

For serum collection, whole blood was collected via cardiac puncture using a sterile 1 mL syringe and 25G needle. Blood was transferred into serum separator tubes (SST) without anticoagulants (BD Microtainer, Cat#365967). Samples were allowed to clot at room temperature for 30 minutes, followed by centrifugation at 2,000 g for 10 minutes at 4°C. The serum supernatant was carefully collected without disturbing the clot. Sera were aliquoted into sterile microcentrifuge tubes and stored at -80°C until cytokine analysis.

For skin lysate preparation, skin samples were harvested from euthanized mice, rinsed briefly in ice-cold PBS to remove blood and debris, and snap-frozen in liquid nitrogen or stored at -80°C until processing. Frozen skin tissues were weighed and placed in pre-chilled lysing matrix tubes (tubes containing ceramic beads (Precellys, Cat#10011151). Lysis buffer (Gibco, Cat#A10492-01) supplemented with protease and phosphatase inhibitor (Thermo Scientific, Cat# 78842) was added at 10 μL per mg of tissue. Samples were homogenized using a bead homogenizer (Precellys Evolution Homogenizer, P000062-PEVO0-A) for 2 × 30 seconds at 6.5 m/s with a 5-minute interval on ice between cycles to prevent overheating. Homogenates were centrifuged at 10,000 g for 10 minutes at 4°C. The supernatant was collected, and total protein concentration

was determined using a Pierce BCA protein assay (Thermo Fisher Scientific, Cat#23227). Aliquots of clarified lysate were stored at -80°C until analysis.

The concentration of cytokines in mouse serum samples and mouse skin lysate samples was analyzed with V-PLEX Proinflammatory Panel 1 Mouse Kit (MSD, Cat#K15048D) per manufacturer's instructions. Electroluminescence was measured with a MESO SECTOR S600 instrument and analyzed using Discovery Workbench 4.0 (MSD). IL-13 was measured by ELISA using the Mouse IL-13 ELISA Kit (Invitrogen, cat#BMS6015) according to the manufacturer's instructions.

Histopathological Analysis

Freshly collected full-thickness intrascapular skin samples were fixed in 10% neutral buffered formalin for a minimum of 24 hours and post-fixed in ethanol. Samples were paraffin-embedded, sectioned at a standard thickness of 4 μm, and stained with hematoxylin and eosin (H&E) at Cureline, Inc. (South San Francisco, CA). Digital scanning of stained slides was performed to facilitate virtual assessment by a board-certified veterinary pathologist. Two non-serial skin sections per animal were reviewed for each animal and scored using a 5-point scale for grading disease severity according to predefined criteria for inflammation, cellular infiltrate, hyperplasia, fibroplasia, and structural integrity. Each anatomical skin layer-epidermis, dermis, panniculus, and subcutis-was assessed and assigned a severity grade. The sum of individual layer scores (potential maximum score of 20) constituted the Total (composite) Pathology Score, representing the overall grade of histopathological changes for each animal. Scores were defined as follows: Normal intrascapular skin; Grade 1 pathology (minimal epidermal

hyperplasia (acanthosis of <100 μm) and hyperkeratosis, minimal mixed dermal inflammation, minimal mixed inflammatory cell infiltrates within the panniculus, and a normal subcutis); Grade 2 pathology (mild epidermal hyperplasia (acanthosis of <150 μm) and hyperkeratosis (with crusts, intraepithelial pustules and spongiosis), mild diffuse dermal inflammation with foci of subepidermal fibrosis, mild pannicular mixed inflammatory cell infiltrates with perivascular cuffing, and minimal to mild diffuse mixed inflammatory infiltrates in the subcutis); Grade 3 pathology (similar, but more frequent and severe features (including ulcerations) within the epidermis, moderate mixed dermal inflammation with prominent subepidermal fibrosis, moderate pannicular mixed inflammation and bridging fibroplasia, and mild to moderate diffuse mixed inflammatory infiltrates in the subcutis); Grade 4 pathology (similar, but more severe features of inflammation including regional epithelial separation and full thickness ulceration of the epidermis, marked mixed inflammation with focal to diffuse fibroplasia (loss of adnexa), marked pannicular inflammation and fibroplasia with reduced adipocytes, and moderate diffuse mixed inflammatory infiltrates with foci of neutrophil aggregation in the subcutis); Grade 5 pathology (severe) was not observed in this study.

Design and generation of tool molecules

All recombinant proteins used in this study included monoclonal antibodies (mAbs) and IL-2 fusion proteins. The IL-2 proteins included mouse IL2WT (1-149, T3A, P51T, C140A) and IL2Mut24 (1-149, T3A, P51T, N103R, V106D, C140A), each fused to the C-terminus of mouse Fc domains or mAbs (17). Residue numbering was based on position 1 as the first residue after sequence peptide cleavage. The substitutions T3A, P51T, and

C140A were introduced to improve construct manufacturability and did not alter the biological activity of IL2WT or IL2Mut24 (17).

DNA fragments were ordered from either IDT (Coralville, IA) or Twist Bioscience (South San Francisco, CA). The gene fragments were assembled using Golden-Gate cloning technology into proprietary mammalian expression vectors compatible with the piggyBac (PB) transposase system (52). All constructs were verified by Sanger DNA sequencing.

All recombinant molecules were stably expressed in CHOK1 (Amgen Inc.) suspension cells using transposase-based technology described previously (52). Protein titers of conditioned media (CM) were estimated by Octet quantitation assays (Protein A Biosensors, ForteBio cat#18-0004) or SDS-PAGE analysis (Invitrogen, NuPAGE™ Bis-Tris Midi Protein Gels).

All recombinant proteins were captured via pre-packed protein A columns (Cytiva, HiTrap™ MabSelect™ SuRe) on AKTA FPLC systems. The CMs obtained using the expression procedure described above were loaded and washed on columns equilibrated in TBS (20 mM Tris, 150 mM NaCl, pH 7.5). Captured proteins were eluted with 0.5% acetic acid, 150 mM NaCl, pH 3.5. The eluents were further polished by size exclusion chromatography (SEC) (HiLoad Superdex 200 pg, Cytiva, Cat#28989336) in HBS (HEPES: 30 mM HEPES; 150 mM NaCl; pH 7.6). The final purified proteins were formulated in HBS or A52Su (Acetate: 10 mM NaOAc; 9% Sucrose; pH 5.2) for storage and filtered with 0.2 μm filters with posidyne membrane (Cytiva, Cat#50-206-3123). Protein concentrations were determined by Nanodrop spectrophotometry for

concentration (NanoDrop-8000, Thermo Fisher Scientific), and all samples were stored at -70°C.

Protein purity was assessed by analytical SEC on an Agilent 1200 series HPLC, using either: (1) a Zenix-C SEC-300 column (3 μm, 300 × 4.6 mm; Sepax, Cat#233300-4630), or (2) a TSK Gel 3000SWXL column (7.8 mm I.D. × 30 cm; TOSOH Bioscience, Cat#0008541). The Zenix-C column was operated at 0.45 mL/min and 12-minute runtime, using a mobile phase consisting of 100 mM sodium phosphate and 250 mM NaCl at pH 6.8. The TSK Gel column was run at 1 mL/min, with a mobile phase consisting of 50 mM phosphate buffer and 0.4 M sodium perchlorate at pH 6.8. For comparison, protein purity was also assessed by SDS-PAGE.

Intact mass analysis was performed using an Agilent 1200 HPLC system coupled with Agilent 6230 TOF mass spectrometer. Non-reduced samples were prepared to contain 5 μg of protein in 0.05% formic acid in water. Reduced samples were prepared by heating the 5 μg of sample in 4M guanidine hydrochloride and 16.7 mM TCEP at 60°C for 30 min, then chilling briefly before adding formic acid to a final concentration of 0.05% in water. Glycosylated samples were deglycosylated by reduced or non-reduced Rapid PNGase F as described previously (New England Biolabs, reduced: Cat#P0704S, non-reduced: Cat#P0711S)(52). 2-5 μg of proteins were injected onto a 2.1 mm x 75 mm C4 reverse phase column (Mac-mod) equilibrated and washed by 0.1% formic acid in water. Proteins within each sample were separated using a gradient of 5-95% buffer B (0.1% formic acid in acetonitrile) at a flow rate of 0.75 mL/min for 12 minutes prior to MS analysis. The full MS scan was obtained over the m/z range of 500-3500.

The endotoxin content of purified proteins was measured using Endosafe LAL cartridges and the Nexgen-MCS system (Charles River). All samples used in this study contained endotoxin less than 0.2 EU/mg.

CTLL-2 and ILC2 Proliferation and ILC2 CD25 Upregulation Assays

To assess IL-2 bioactivity, [^{3}H]-thymidine incorporation assays were performed using IL-2-dependent CTLL-2 cells (ATCC, Cat# TIB-214™) or purified mouse ILC2s. CTLL-2 cells were maintained in complete RPMI-1640 supplemented with 10% FBS, 1 mM sodium pyruvate, 1× non-essential amino acids, 10 mM HEPES, 1× penicillin-streptomycin, and recombinant human IL-2 (PeproTech, Cat# 200-02) at a density of 0.2-1 × 10^6 cells/mL. Cells were washed and deprived of IL-2 overnight before use.

For ILC2 isolation, *Rag2*$^{-/-}$ mice were administered 4 μg IL-33 five times over one week. Lungs were harvested, and ILC2s were purified using the Mouse ILC2 Enrichment Kit (STEMCELL Technologies, Cat# 19842). Purified ILC2s were expanded at 0.5 × 10^6 cells/mL in complete RPMI containing 20 ng/mL IL-2 for 4 days, washed twice, and cultured overnight in IL-2-free T-cell medium before use.

CTLL-2 cells (10,000 cells/well) or ILC2s (80,000 cells/well) were plated in flat-bottom 96-well plates and stimulated with half-log serial dilutions of each IL-2 construct from 100 nM to 32 pM. After 48 hours, cells were pulsed with 1 μCi/well [^{3}H]-thymidine for an additional 18 hours. Cells were harvested onto filter membranes, and incorporated radioactivity was quantified using a MicroBeta^2 scintillation counter. Results were reported as counts per minute (CPM).

To assess CD25 upregulation, IL-33-expanded ILC2s were cultured directly with IL-2 constructs at 1 nM. CD25 surface expression was measured by flow cytometry at 0, 2, 4, and 8 hours with anti-CD25 (clone: 7D4).

Active Induction of EAE

Experimental autoimmune encephalomyelitis (EAE) was induced in female C57BL/6 mice (8-14 weeks old) by subcutaneous immunization with MOG_{35-55} peptide emulsified in complete Freund's adjuvant (CFA). Mice received a total of 200 μL of CFA (1 mg/mL MOG_{35-55}; 1 to 5 mg killed mycobacterium tuberculosis H37Ra/mL; Hooke Laboratories, Cat#EK-2110), administered at two sites (100 μL per flank; right and left hind flanks). On the day of immunization (day 0) and again on day 2, mice were intraperitoneally (i.p.) injected with 120 ng of pertussis toxin (PTx, Hooke Laboratories, Cat#BT-0105) diluted in sterile saline. Anti-CD4 (Clone GK1.5, BioXCell, cat#BE0003-1) was used as a control in the study. Following immunization, mice were monitored daily for body weight and clinical signs of EAE using the following scoring system: 0 indicated no clinical signs; 0.5, partial limp tail or body tremors; 1, limp tail with normal gait; 2, limp tail and hind limb weakness (waddling gait); 3, partial hind limb paralysis; 4, complete hind limb paralysis; and 5, severe forelimb paralysis, moribund condition, or humane endpoint. Body weights were normalized to the day 0 value (set as 100%) and tracked daily as a percentage change. The mean clinical EAE score was calculated as the average of daily scores across the disease course for each mouse. Mice that reached a score of 5 were euthanized and excluded from further clinical score and weight analysis from that point forward.

Cynomolgus Monkey Studies

Animals and Ethical Compliance

Naïve cynomolgus monkeys (*Macaca fascicularis*) were housed under controlled environmental conditions with a 12-hour light/dark cycle and provided environmental enrichment according to institutional standard operating procedures. Monkeys were individually housed for intensive observation following dosing and pair- or group-housed thereafter when compatible. Animals were monitored daily for clinical signs and overall health throughout the study.

Study Design and Dosing

In study 118075, male cynomolgus monkeys (n = 4 per group) received either five daily s.c. injections of 3 or 10 μg/kg aldesleukin on days 0 to 4 followed by 30 or 100 μg/kg on days 13-17) or a single s.c. injection of 1 or 3 μg/kg AMG592 on day 0 and 100 or 200 μg/kg on day 13 (Figure 5A).

In study 118065, monkeys were assigned to treatment groups (n = 4 per sex per group) and administered s.c. injections of AMG592 (10, 30 or 100 μg/kg) once weekly for three months (total of 12 doses) (Figure 5H).

Blood collection, immunophenotyping, serum cytokine analysis

In study 118075, for immunophenotyping, peripheral blood was collected via the femoral vein or artery in K_2EDTA tubes pre-dose (days -15, -8, and day 0 prior to 1st dose) and on days 3, 4, 5, 12, 14, 18, 21, 24, and 28. For cytokine analysis, whole blood was collected at the following time points and processed to serum: pre-dose (days -8 and day

0 prior to dosing), days 4, 5, 16, 17, 18, and 35. Serum cytokines were quantified using Milliplex®MAP Non-HumanPrimate Cytokine Magnetic Bead Panel (Millipore, Cat# PRCYTOMAG-40K-10) according to manufacturer instructions. Cytokine concentrations were determined relative to standard curves generated using a 5 parameter logistic non-linear regression fitting curve. The following cytokines were measured: IFNγ, GM-CSF, TNF, IL1-β, IL-2, IL-5, IL-6, IL-10, IL-12p40, IL-13, and IL-17. Cytokines detected above the assay limit were shown in Figure 5C-G.

Clinical Monitoring and Injection-Site Pathology

In study 118065, at scheduled necropsy (day 92), the designated subcutaneous injection site was collected for histopathologic evaluation. Tissues were fixed, processed, and stained with hematoxylin and eosin according to standard toxicologic pathology procedures, and lesions were evaluated by a board-certified veterinary pathologist and graded by severity (e.g., minimal or mild).

References

49. Hampton AL, Hish GA, Aslam MN, Rothman ED, Bergin IL, Patterson KA, et al. Progression of ulcerative dermatitis lesions in C57BL/6Crl mice and the development of a scoring system for dermatitis lesions. *J Am Assoc Lab Anim Sci*. 2012;51(5):586–93.
50. Humbert P, Maibach H, Fanian Md Phd F, and Agache P. *Agache's Measuring the Skin: Non-invasive Investigations, Physiology, Normal Constants: Second Edition*. 2017.
51. Neu SD, Strzepa A, Martin D, Sorci-Thomas MG, Pritchard KA, Jr., and Dittel BN. Myeloperoxidase Inhibition Ameliorates Plaque Psoriasis in Mice. *Antioxidants (Basel)*. 2021;10(9).
52. Sun H, Wang S, Lu M, Tinberg CE, and Alba BM. Protein production from HEK293 cell line-derived stable pools with high protein quality and quantity to support discovery research. *PLoS One*. 2023;18(6):e0285971.
53. Rickert M, Wang X, Boulanger MJ, Goriatcheva N, and Garcia KC. The structure of interleukin-2 complexed with its alpha receptor. *Science*. 2005;308(5727):1477–80.
54. Goubier A, Merchiers P, Salimu J, Goyenechea B, Moulder K, Quezada S, Peggs K, Vargas FA, Solomon I. Fc-optimized anti-CD25 for tumour specific cell depletion. WO patent WO2018167104A1. Published September 20, 2018.

SUPPLEMENTAL FIGURES

Supplemental Figure 1

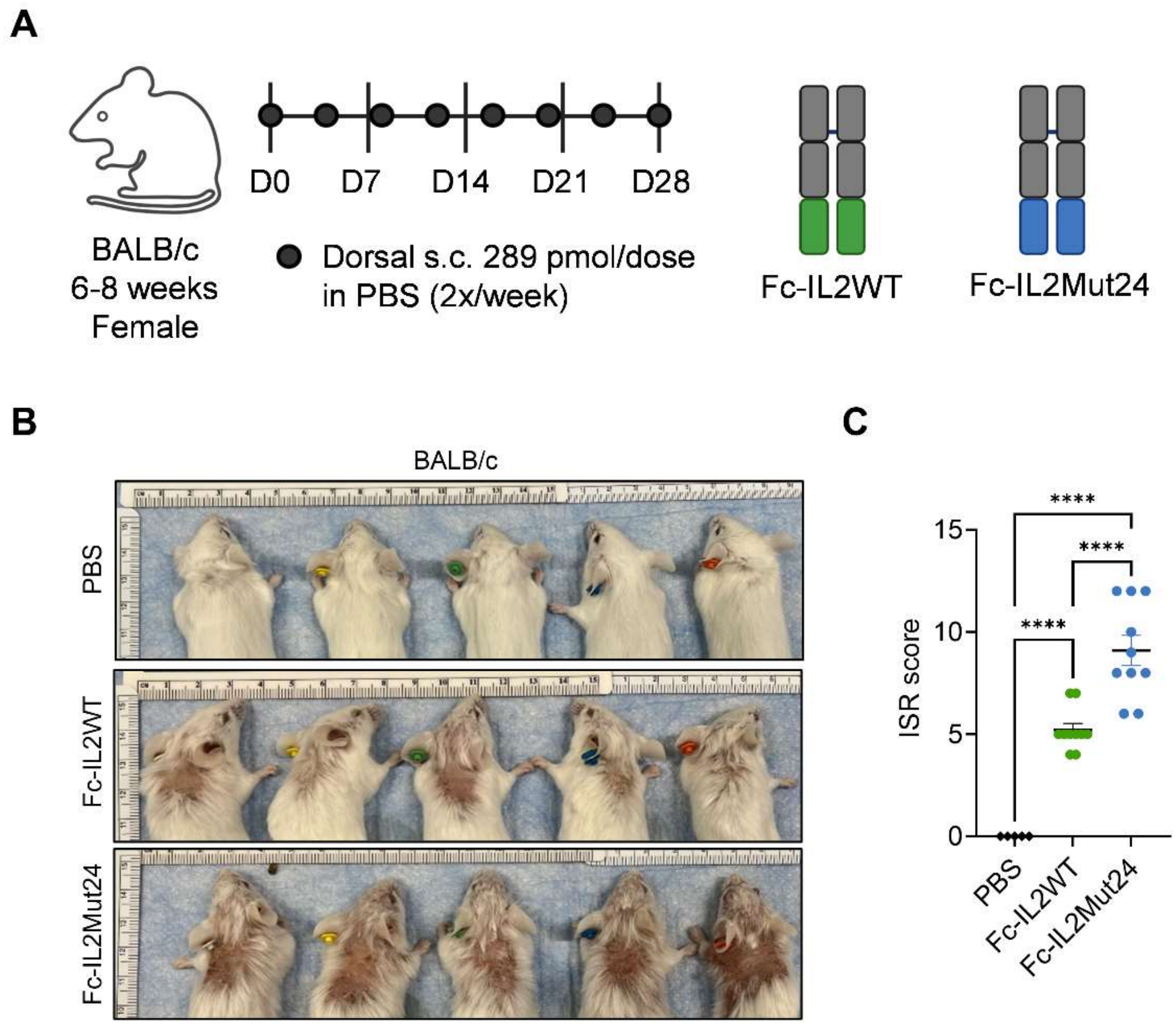

Supplemental Figure 1. Fc-IL2Mut24 induces severe ISRs associated with prolonged systemic and cutaneous exposure. 6-8-week-old female BALB/c mice received dorsal s.c. dosing of either Fc-IL2WT or Fc-IL2Mut24 289 pmol (~25 μg/dose/mouse in PBS) or PBS alone twice per week for four weeks. **(A)** Schematic representation of experimental design. **(B)** Representative photograph of dorsal ISR (n=5 mice per group). **(C)** ISR score (n=5 mice in PBS, n=10 mice each in Fc-IL2WT and Fc-IL2Mut24). Two-tailed ordinary one-way ANOVA followed by Sidak's post hoc test, *p<0.05, **p<0.01, ***p<0.001, ****p<0.0001.

Supplemental Figure 2

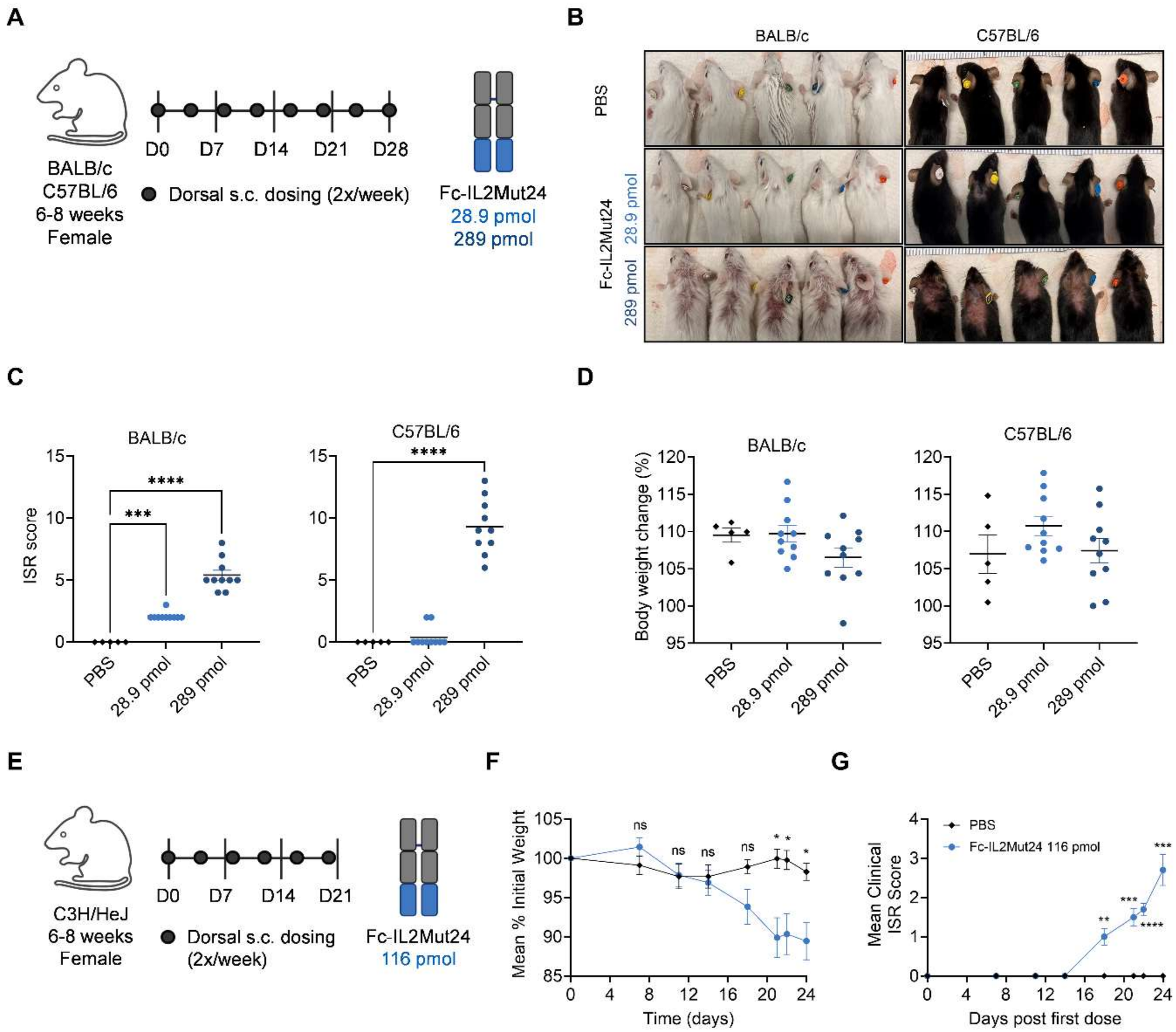

Supplemental Figure 2. Fc-IL2Mut24 induced ISRs across BALB/c, C57BL/6J, and C3H/HeJ. (A-D) BALB/c (left) and C57BL/6J (right) mice were treated s.c. twice weekly for four weeks with PBS, or 28.9 pmol or 289 pmol Fc-IL2Mut24. **(A)** Schematic representation of experimental design. **(B)** Representative images from n=5 mice per strain after eight doses of Fc-IL2Mut24. **(C)** ISR score, **(D)** Body weight change from day 0 (%) at endpoint. **(E-G)** In a separate cohort, C3H/HeJ mice were immunized with 116 pmol Fc-IL2Mut24 twice weekly for three weeks, while a control group remained naïve and untreated. **(E)** Schematic representation of experimental design. **(F)** Body weight over time (days) as % change from initial weight. **(G)** ISR score over time (days). **(C-D)** n=5 mice per strain in the PBS condition, n=10 mice per strain in the 28.9 pmol and 289 pmol Fc-IL2mut24 conditions. **(F-G)** n=10 mice per condition. Data shown as mean±SEM. **(C)** Two-tailed ordinary one-way ANOVA followed by Sidak's multiple comparisons post hoc test; **(F)** Two-tailed mixed effects analysis followed by Sidak's multiple comparisons post hoc test**.** (**G**) Two-way ANOVA followed by Sidak's multiple comparisons post hoc test**.** *$p < 0.05$, **$p < 0.01$, ***$p < 0.001$, ****$p < 0.0001$**.**

Supplemental Figure 3

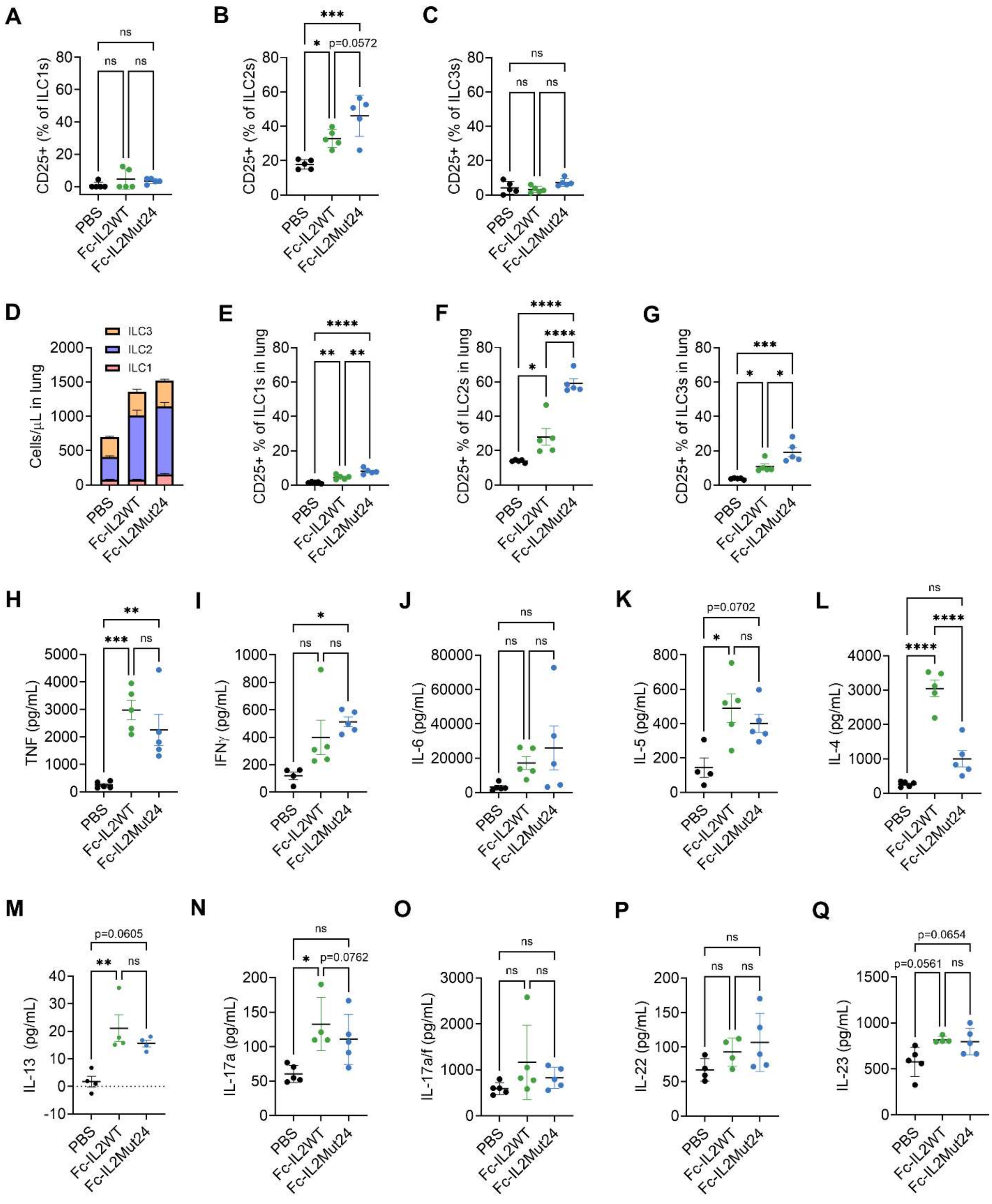

Supplemental Figure 3. Fc-IL2Mut24 induces a CD25-dependent type 2 inflammatory program. (A-G) C57BL/6 mice (n=5 mice per group) received dorsal s.c. dosing twice weekly for four weeks with PBS, 69 pmol (~6.5 μg/dose/mouse) Fc-IL2WT, or Fc-IL2Mut24. **(A-C)** Frequency of $CD25^+$ cells among ILC1s **(A)**, ILC2s **(B)** and ILC3s **(C)** in skin at endpoint, as analyzed by flow cytometry. **(D)** ILC1, ILC2 and ILC3 cell counts per sample in lung at endpoint represented as stacked bar graph, as analyzed by flow cytometry. **(E-G)** Frequency of $CD25^+$ cells among ILC1s **(E)**, ILC2s **(F)** and ILC3s **(G)** in lung at endpoint, as analyzed by flow cytometry. **(H-Q)** Cytokine levels of TNF (**H**), IFNγ (**I**), IL-6 **(J)**, IL-5 **(K)**, IL-4 **(L)**, IL-13 **(M),** IL-17A **(N)**, IL-17A/F**(O)**, IL-22 **(P)**, and IL-23 **(Q)**, shown as concentration (pg/mL) from endpoint skin homogenates as detected by MSD. Two-tailed ordinary one-way ANOVA followed by Sidak's multiple comparisons post hoc test; *$p < 0.05$, **$p < 0.01$, ***$p < 0.001$, ****$p < 0.0001$**.**

Supplemental Figure 4

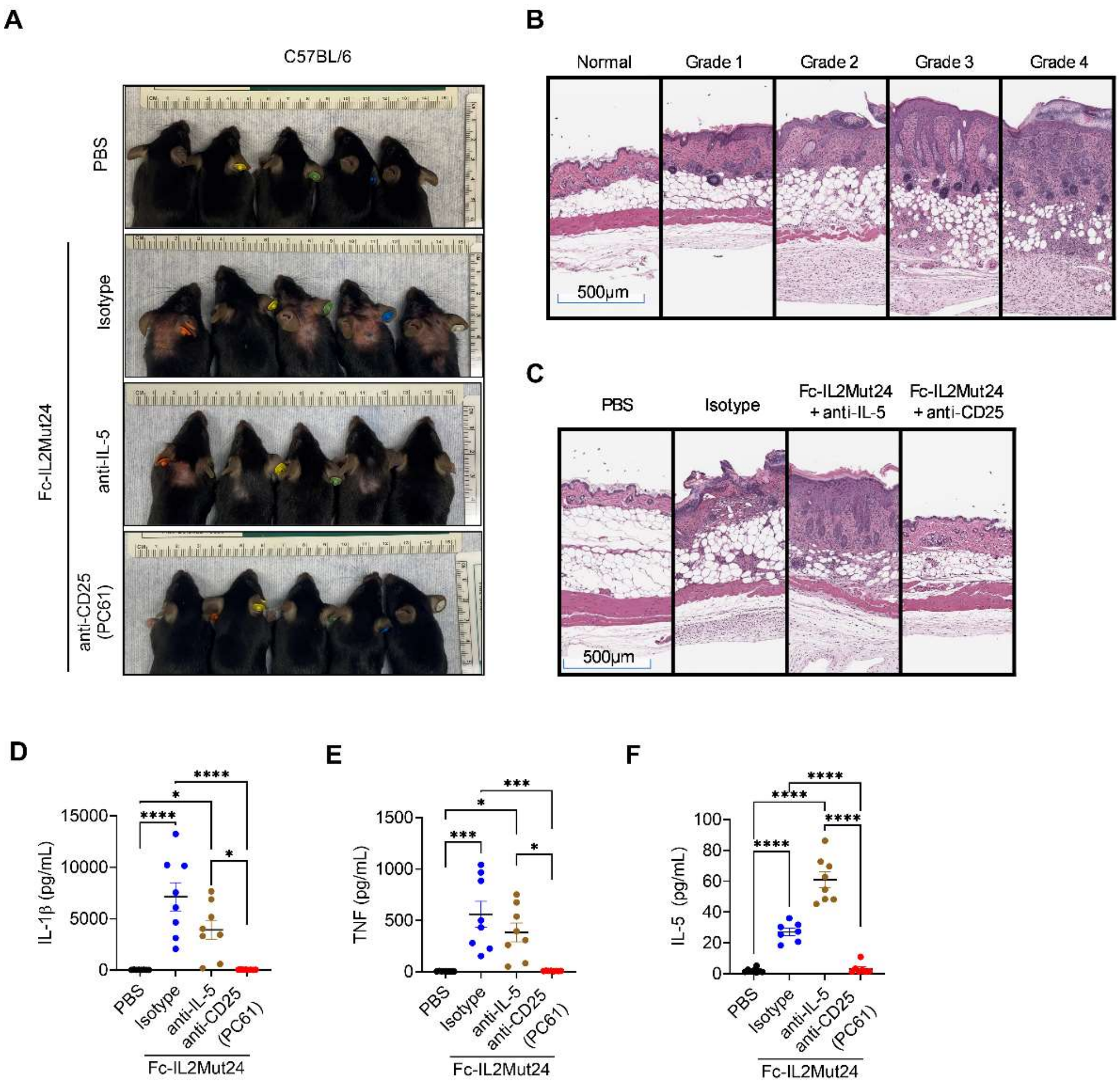

Supplemental Figure 4. Anti-CD25 blockade reduces IL-2–induced skin pathology. (A-G) C57BL/6 mice (n=10 mice per group) received dorsal s.c. dosing twice weekly for four weeks with PBS or 69 pmol (~6.5 μg/dose/mouse) Fc-IL2Mut24 and co-treated once weekly with 200 μg anti-IL-5, anti-CD25 or isotype control. **(A)** Representative dorsal images (n=5 mice per group). **(B)** Representative H&E staining images showing range of ISR severity in C57BL/6J mice following eight doses of Fc-IL2Mut24 (scale bar = 500 μm, 4x magnification). **(C)** Representative H&E staining images from one mouse per treatment condition (scale bar = 500 μm, 4x magnification) from the experiment shown in Figure 3H. Mice were selected to represent the mean pathology score for their respective treatment conditions (PBS = 0, isotype = 9, anti-IL-5 = 8, anti-CD25 = 1.5). **(D-F)** Inflammatory cytokine levels of IL-1β **(D)**, TNF **(E)**, IL-5 **(F)**, shown as concentration (pg/mL) from endpoint skin homogenates as detected by MSD. Data shown as mean ± SEM. Two-tailed ordinary one-way ANOVA followed by Sidak's multiple comparisons post hoc test; only selected comparisons are shown. *$p < 0.05$, **$p < 0.01$, ***$p < 0.001$, ****$p < 0.0001$**.**

Supplemental Figure 5

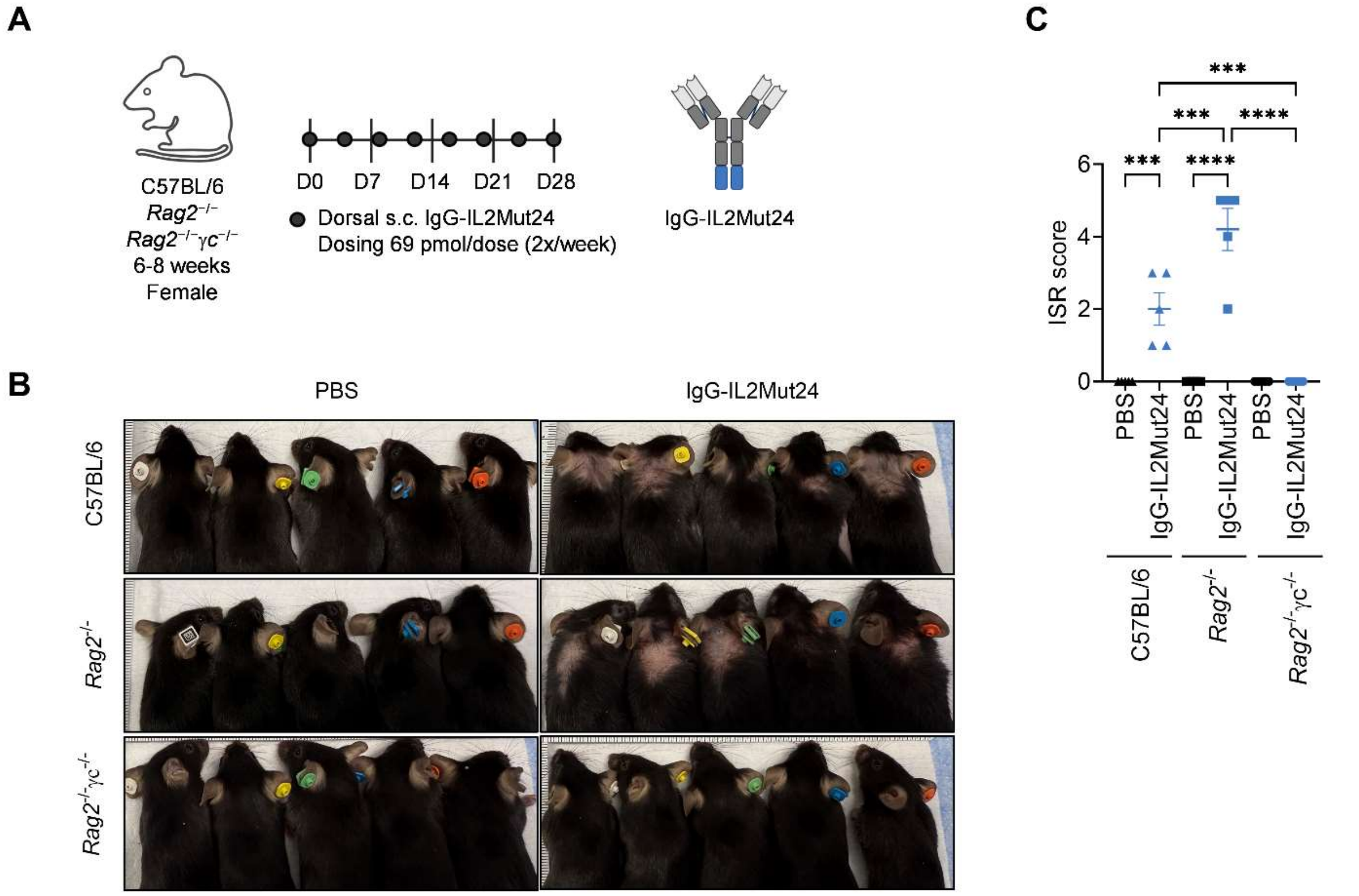

Supplemental Figure 5. γc deficiency protects from IL-2-induced injection site reaction. (A-C) C57BL/6NTac, *RAG2*$^{-/-}$ or *RAG2*$^{-/-}$ $\gamma c^{-/-}$ (n=5 mice per group) were treated subcutaneously twice weekly for four weeks with 69 pmol IgG-IL2Mut24. **(A)** Schematic representation of experimental design. **(B)** Dorsal images. **(C)** ISR scores at endpoint. Data shown as mean±SEM. Two-tailed ordinary one-way ANOVA followed by Sidak's multiple comparisons post hoc; only selected comparisons are shown. *p < 0.05, **p < 0.01, ***p < 0.001, ****p < 0.0001**.**

Supplemental Figure 6

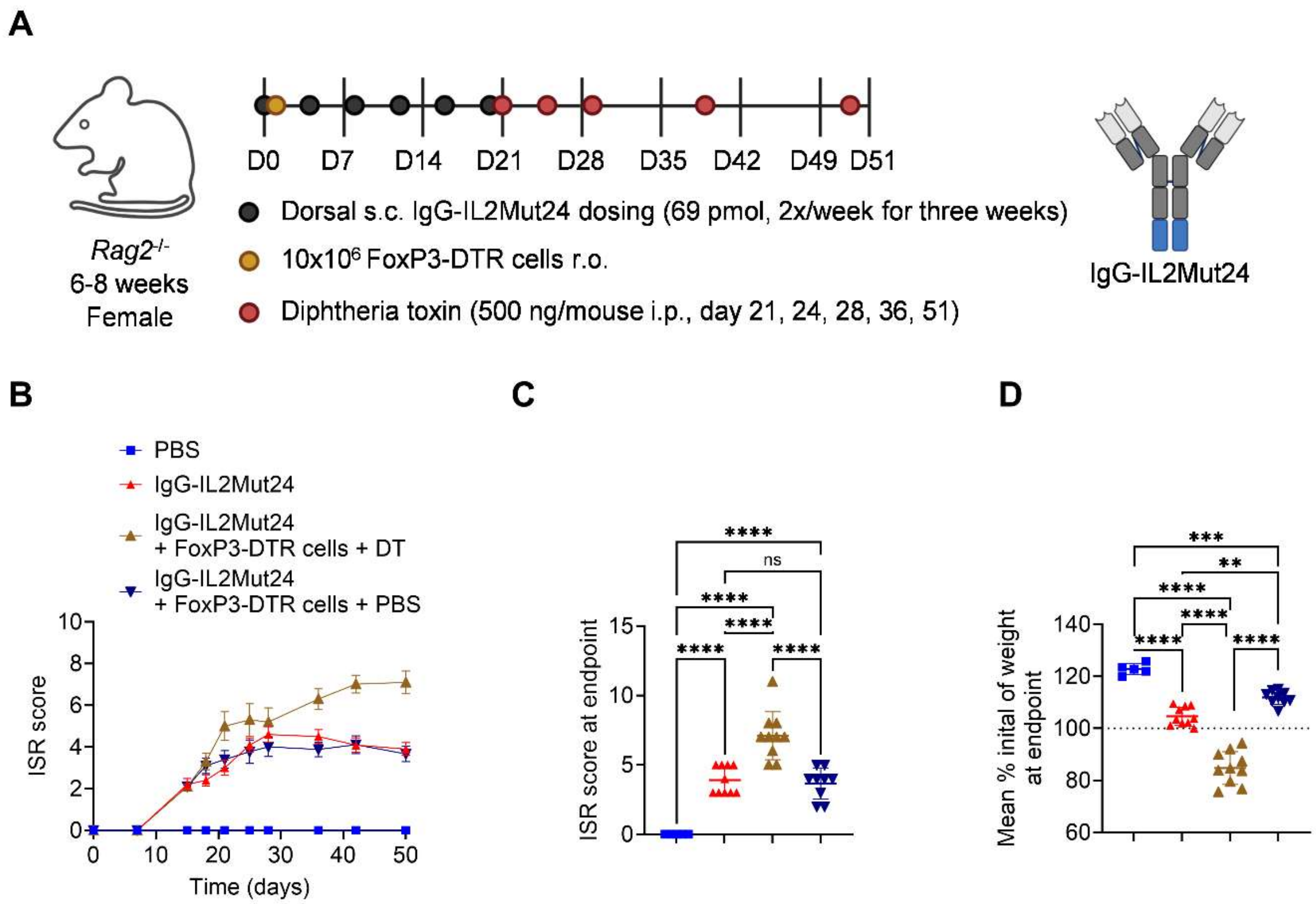

Supplemental Figure 6. Treg depletion exacerbates IL2Mut24-induced ISR and weight loss in immune-reconstituted mice.

(A-D) 6-8-week-old female Rag2$^{-/-}$ mice were transferred retro-orbitally with 10 million FoxP3-DTR cells (enriched T cells) in 50 µL of PBS and received 69 pmol of IgG-IL2Mut24 in 100 µL of PBS s.c. twice per week for three weeks. Diphtheria toxin (500ng/mouse in 100µL PBS, i.p. on days 21, 24, 28, 36, and 51) was administered following the last dose of IgG-IL2Mut24 (after ISR onset). **(A)** Schematic representation of experimental design. **(B)** ISR scores over time (days) at the injection site. **(C)** ISR scores at the injection site at endpoint. **(D)** Body weight at endpoint as % change from initial weight. n=5 mice in the PBS condition and n=10 mice in all other conditions. Data shown as mean±SEM. Two-tailed ordinary one-way ANOVA analysis followed by Sidak's multiple comparisons post hoc test in **C** and **D**; *p < 0.05, **p < 0.01, ***p < 0.001, ****p < 0.0001.

Supplemental Figure 7

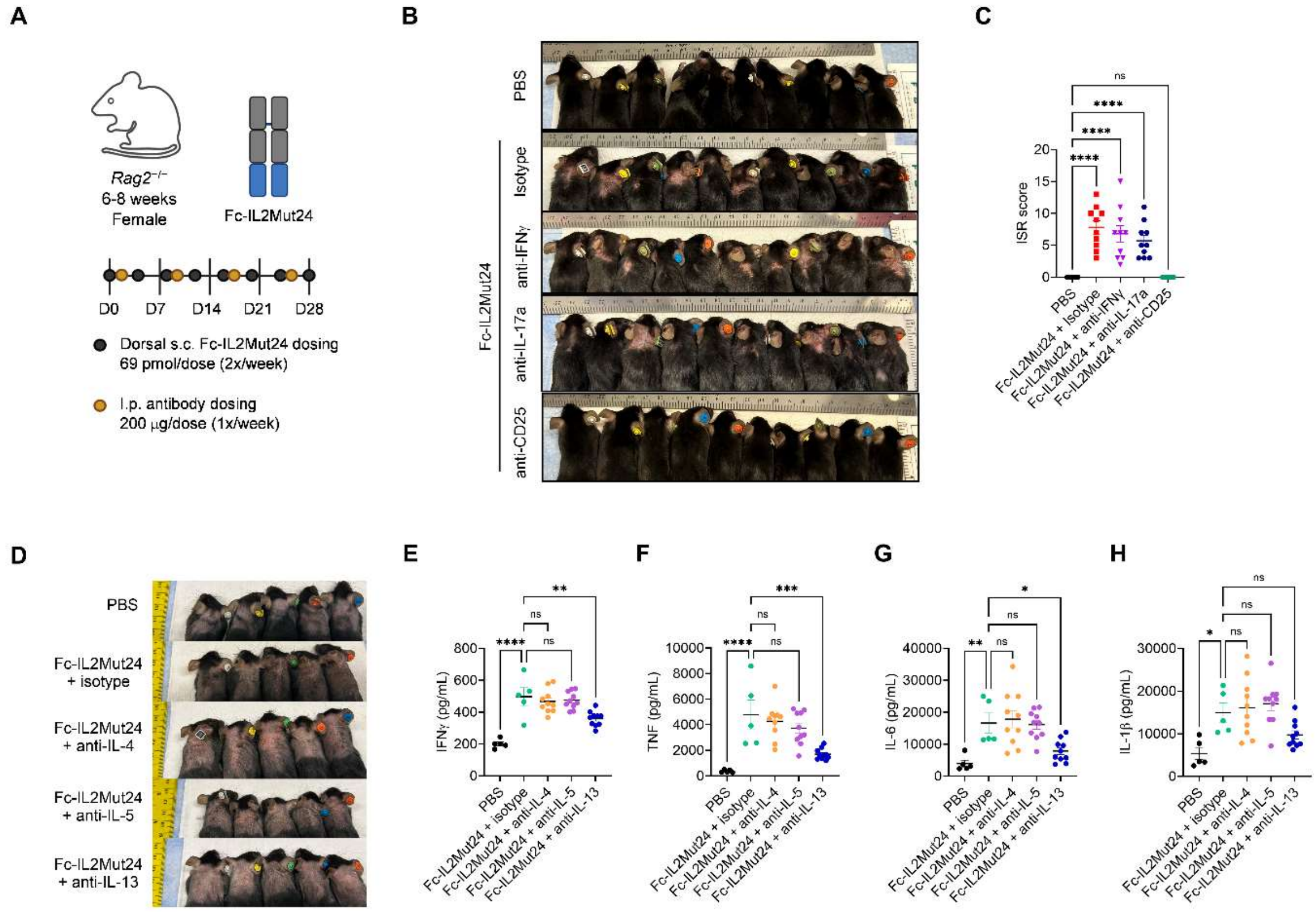

Supplemental Figure 7. Effects of CD25 and cytokine blockade on Fc-IL2Mut24-induced ISR in *Rag2*$^{-/-}$ mice.

(A-C) *Rag2*$^{-/-}$ mice (n=10 mice per group) received dorsal s.c. dosing twice weekly for four weeks with PBS or 69 pmol (~6.5 μg/dose/mouse) Fc-IL2Mut24 and co-treated once weekly with 200 μg anti-IFNγ, anti-IL-17a, anti-CD25 or isotype control. **(A)** Schematic representation of experimental design. **(B)** Representative dorsal images (n=5 mice per group). **(C)** ISR scores at endpoint. **(D-H)** *Rag2*$^{-/-}$ mice (n=5 mice in PBS and isotype groups and n=10 mice in all other groups) received dorsal s.c. dosing twice weekly for four weeks with 69 pmol (~6.5 μg/dose/mouse) Fc-IL2Mut24 and co-treated once weekly with 200 μg anti-IL-4, anti-IL-5, anti-IL-13 or isotype control. **(D)** Representative dorsal images (n=5 mice per group). Mice were shaved for better visualization of the injection site. **(E-H)** Cytokine levels of IFNγ **(E)**, TNF **(F)**, IL-6 **(G)** and IL-1β **(H)**, shown as concentration (pg/mL) from endpoint skin homogenates as detected by MSD. Data shown as mean±SEM. Two-tailed ordinary one-way ANOVA analysis followed by Sidak's multiple comparisons post hoc test; *p < 0.05, **p < 0.01, ***p < 0.001, ****p < 0.0001.

Supplemental Figure 8

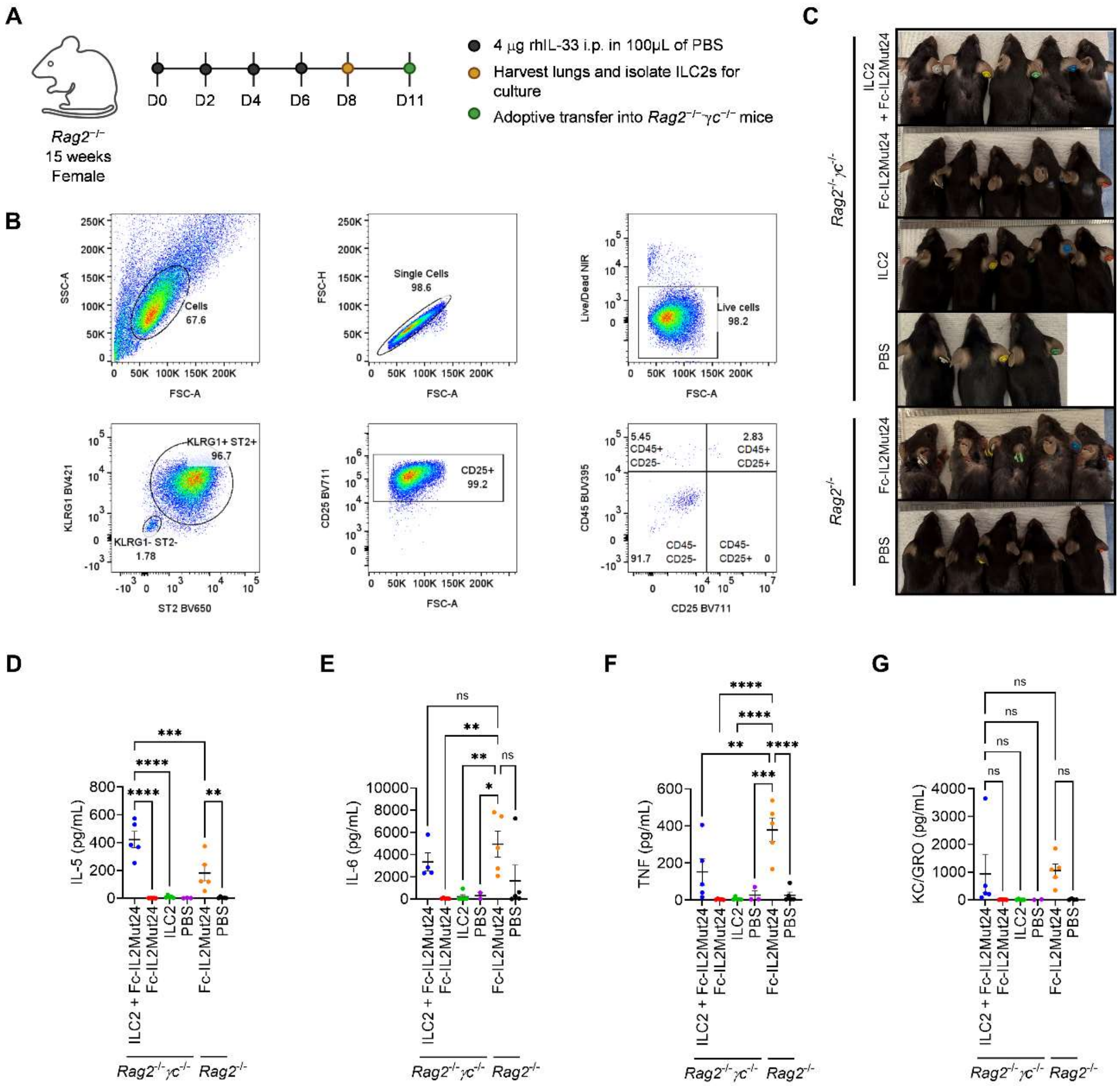

Supplemental Figure 8. Characterization of IL-33-expanded ILC2s and skin cytokine responses after transfer. (A) Schematic representation of experimental design for IL-33-mediated ILC2 expansion in *RAG2*$^{-/-}$ mice. **(B)** Flow cytometry analysis of cultured ILC2 cells prior to adoptive transfer to recipient mice. **(C)** Representative dorsal images (n=5 mice per group) from the experiment shown in Figure 4F. **(D-G)** Concentration (pg per 50 µL of skin lysate) of IL-5 **(D)**, IL-6 **(E)**, TNF **(F)** and KC/GRO **(G)** at endpoint. n=3 mice in PBS group and 5 mice in all other groups. Data shown as mean ± SEM. Two-tailed ordinary one-way ANOVA followed by Sidak's multiple comparisons post hoc test; only selected comparisons are shown. *$p < 0.05$, **$p < 0.01$, ***$p < 0.001$, ****$p < 0.0001$.

Supplemental Figure 9

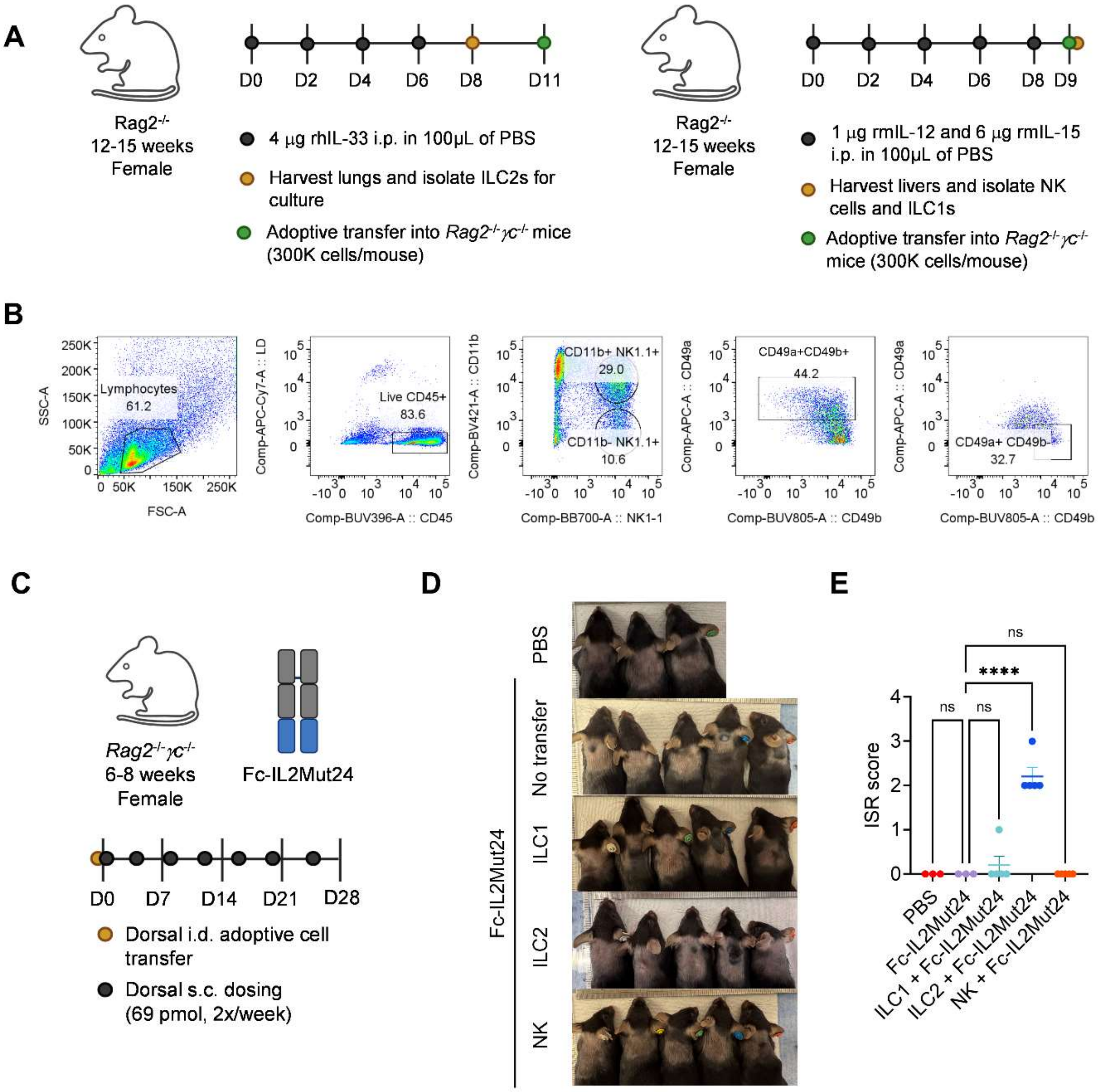

Supplemental Figure 9. Characterization of IL-12+IL-15–expanded ILC1s and NK cells and ISRs after adoptive transfer. (A) Schematic representation of experimental design for IL-33-mediated ILC2 expansion and IL-12 + IL15-mediated ILC1 expansion in *RAG2*$^{-/-}$ mice. **(B)** Gating strategy for cell sorting of ILC1s and NK cells for adoptive transfer to recipient mice. ILC1s were sorted as live CD45^{+}CD11b^{-}NK1.1^{+}CD49a^{+} CD49b^{-}. NK cells were sorted as live CD45^{+}CD11b^{+}NK1.1^{+}CD49a^{+}CD49b^{+}cells. **(C)** Schematic representation of experimental design for adoptive transfer of 3x10^{5} ILC1, ILC2 and NK cell. **(D)** Representative dorsal images (n=5 mice per group). Mice were shaved for better visualization of the injection site. **(E)** ISR scores at endpoint. n=3 mice in PBS group and 5 mice in all other groups. Data shown as mean ± SEM. Two-tailed ordinary one-way ANOVA followed by Sidak's multiple comparisons post hoc test; only selected comparisons are shown. *p < 0.05, **p < 0.01, ***p < 0.001, ****p < 0.0001.

Supplemental Figure 10

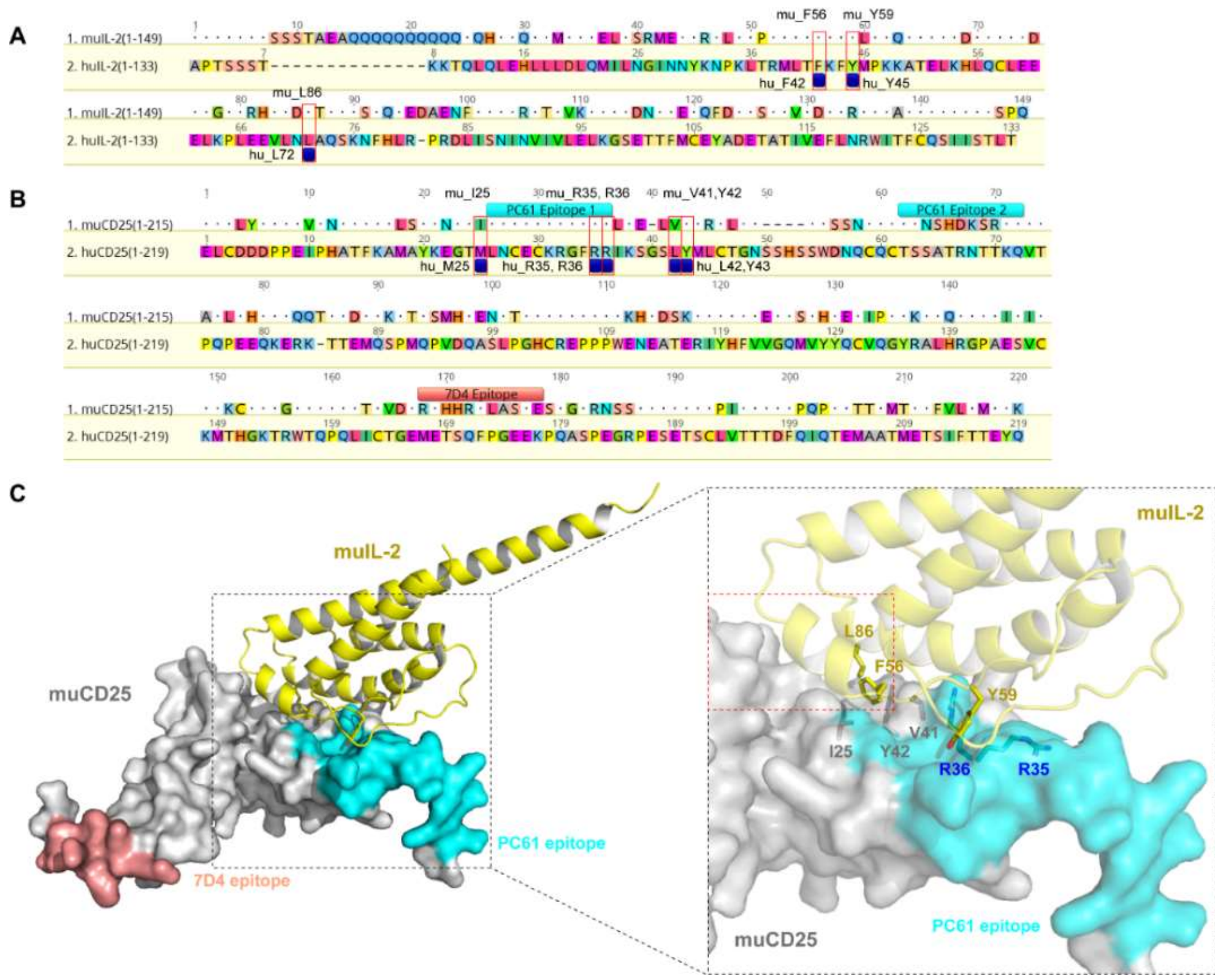

Supplemental Figure 10. The partial blocker PC61 epitope partially overlaps with the IL-2 and CD25 interface. (A-B) Sequence alignment of human (hu) and mouse (mu) IL-2 **(A)** and CD25 **(B)**. (Geneious version 10.2 created by Biomatters). Numbering begins at the first amino acid following signal sequence cleavage. Key interface residues, identified from the crystal structure of huIL-2 and huCD25 (PDB: 1z92), are boxed and labeled (53). Corresponding residues on muIL-2 and muCD25 were inferred using an internally modified version of AlphaFold2 Multimer-modeled structure and sequence alignment. PC61 and 7D4 epitopes, as described in a patent (54), are indicated with cyan and salmon bars, respectively. **(C**, left**)** The complex structure of muIL-2 and muCD25 modeled by an internally modified AlphaFold2 Multimer. MuIL-2 is shown in cartoon representation (yellow), and muCD25 is shown in surface representation (gray). Loop regions of muCD25 (70-95 and 176-215) with low predicted Local Distance Difference Test (pLDDT) scores are omitted for clarity. PC61 and 7D4 epitopes on muCD25 are shown in cyan and salmon, respectively. **(C,** right**)** Close-up view of the boxed region on the left. Key interface residues are shown in stick representation. Residues on muIL2 and muCD25 are colored yellow and cyan, respectively. The PC61 epitope partially overlaps with the muIL-2 and muCD25 interface. One hydrophobic patch-comprising R35 and R36 of muCD25 (corresponding to R35 and R36 in huCD25) interacts with Y59 on muIL2 (Y45 in huIL2) and lies within the PC61 epitope (53). A second hydrophobic patch-forming a recessed pocket of I25, V41, and Y42 on muCD25 (M25, L42, and Y43 in huCD25) interacts with F56 and L86 on muIL2 (F42 and L72 in huIL2) and lies adjacent to the PC61 epitope (53). The N-terminal region of muCD25, which also interacts with muIL2, lies outside the PC61 epitope (red dotted box).

Supplemental Figure 11

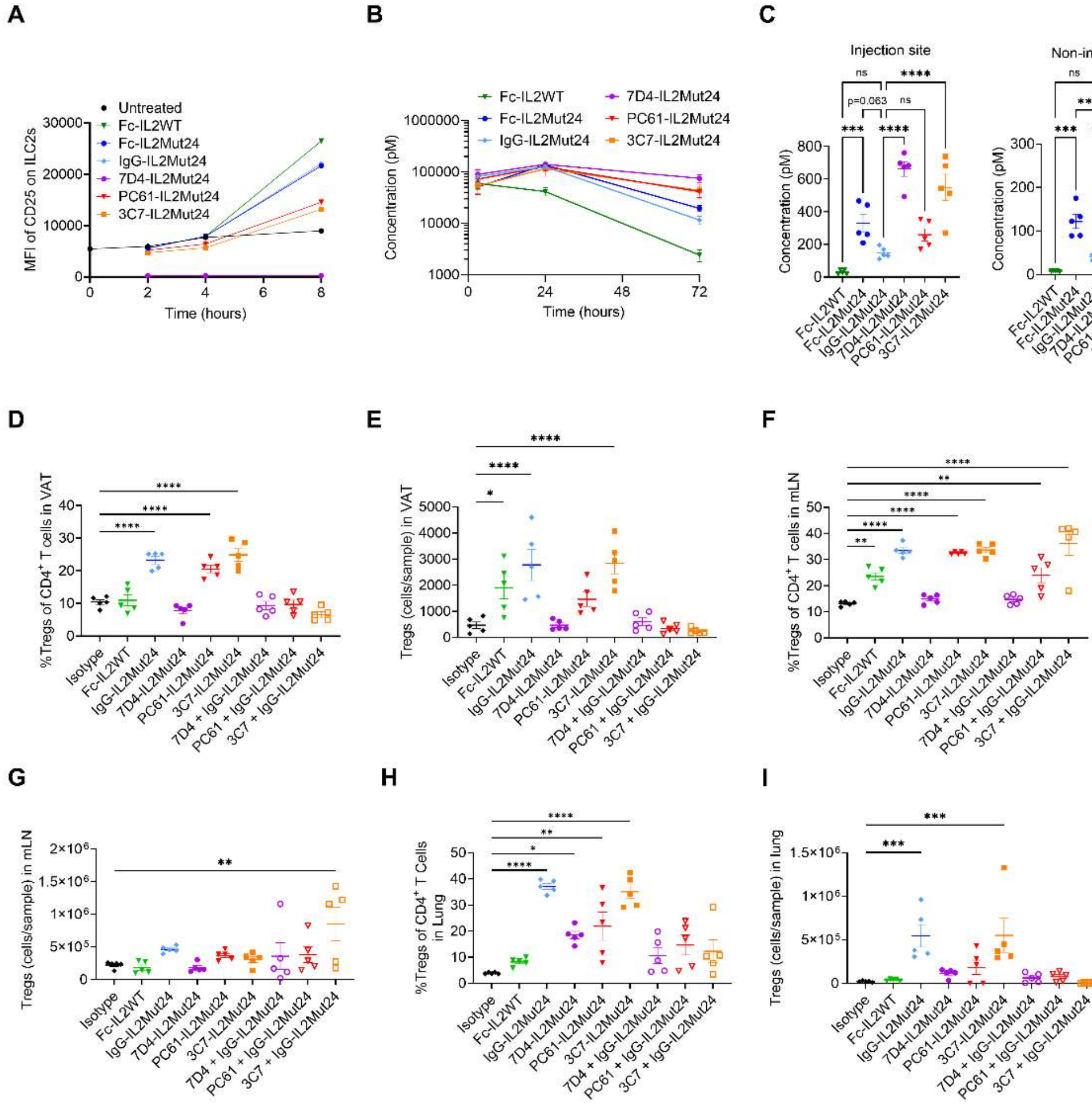

Supplemental Figure 11. Skin biodistribution and tissue response of IL2Mut24 fusion proteins. (A) ILC2s were isolated from the lungs of IL-33-treated *Rag2*$^{-/-}$ mice. Mean fluorescence intensity (MFI) of CD25 on IL-33-expanded ILC2s treated *in vitro* with 1 nM each of Fc-IL2WT and the IL2Mut24 fusion proteins over time (hours) as analyzed by flow cytometry using anti-CD25 (clone:7D4). **(B)** Fc-IL2WT and IL2Mut24 fusion proteins plasma concentrations (pM) at 3, 24 and 72 hours post injection as detected by MSD (n=5 mice/condition). **(C)** Concentrations (pM) of Fc-IL2WT and the IL2Mut24 fusion proteins detected in skin samples from injection-site and non-injection site at 72 hours post injection by MSD (n=5 mice per condition, one representative experiment of two). **(D-I)**. 6-8-week-old female C57BL/6J mice were injected subcutaneously with 69 pmol of either isotype, 7D4-IL2Mut24, PC61-IL2Mut24, 3C7-IL2Mut24, IgG-IL2Mut24, Fc-IL2WT, or matched combinations of unconjugated anti-CD25 clones (7D4, PC61, or 3C7) co-injected with IgG-IL2Mut24. Mice were euthanized on day 4 post-injection. Frequency **(D, F, H)** and absolute counts per sample **(E, G, I)** of CD25$^+$ FOXP3$^+$ cells within the CD4$^+$ T cell compartment (referred to as Tregs) in visceral adipose tissues (VAT) **(D, E),** mesenteric lymph nodes (mLN) **(F, G)**, and lungs **(H, I)** as analyzed by flow cytometry. n=5 mice per group from one representative experiment of two.

Supplemental Figure 12

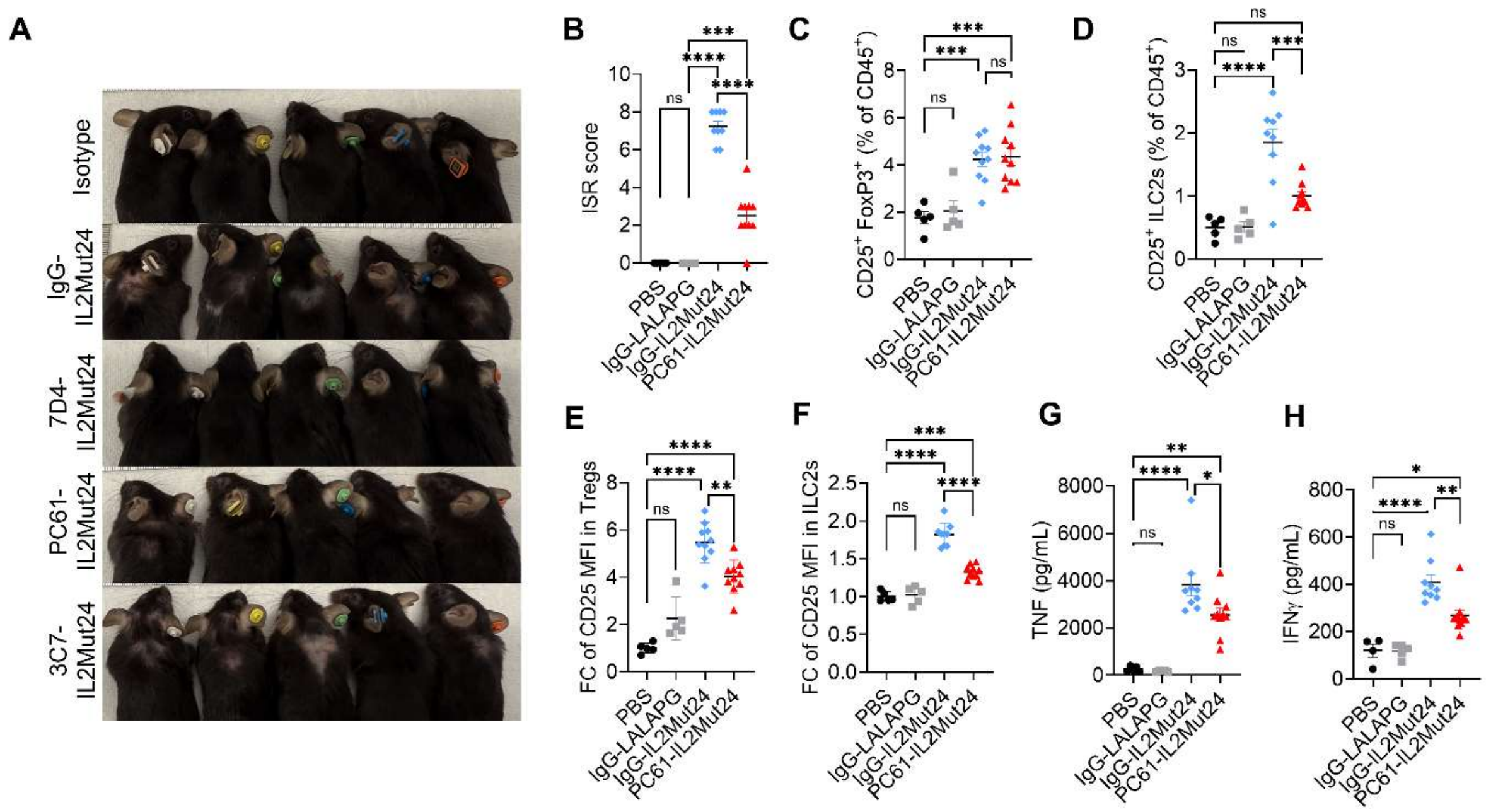

Supplemental Figure 12. PC61-IL2Mut24 reduces ISR by constraining the cutaneous ILC2 response. (A) Representative dorsal images (n=5 mice per group) from experiment shown in Figure 6I-J. **(B-H)** Independent experimental repetition of setup shown in Figure 6I, with n=5 mice in PBS and IgG-LALAPG groups and n=10 mice in IgG-IL2Mut24 and PC61-IL2Mut24 groups. **(B)** ISR scores at endpoint. **(C-D)** Frequency of $CD25^{+}$ $FoxP3^{+}$ cells **(C)** and $CD25^{+}$ ILC2s **(D)** among CD45+ cells in skin, as analyzed by flow cytometry. (**E-F**) CD25 MFI fold changes in Tregs (**E**) and ILC2s (**F**) compared to PBS. **(G-H)** TNF and IFNγ shown as concentration (pg/mL) from endpoint skin homogenates as detected by MSD. Data shown as mean±SEM. Two-tailed ordinary one-way ANOVA followed by Sidak's multiple comparisons post hoc test, only selected comparisons are shown. $^{*}p < 0.05$, $^{**}p < 0.01$, $^{***}p < 0.001$, $^{****} p < 0.0001$**.**

Supplemental Figure 13

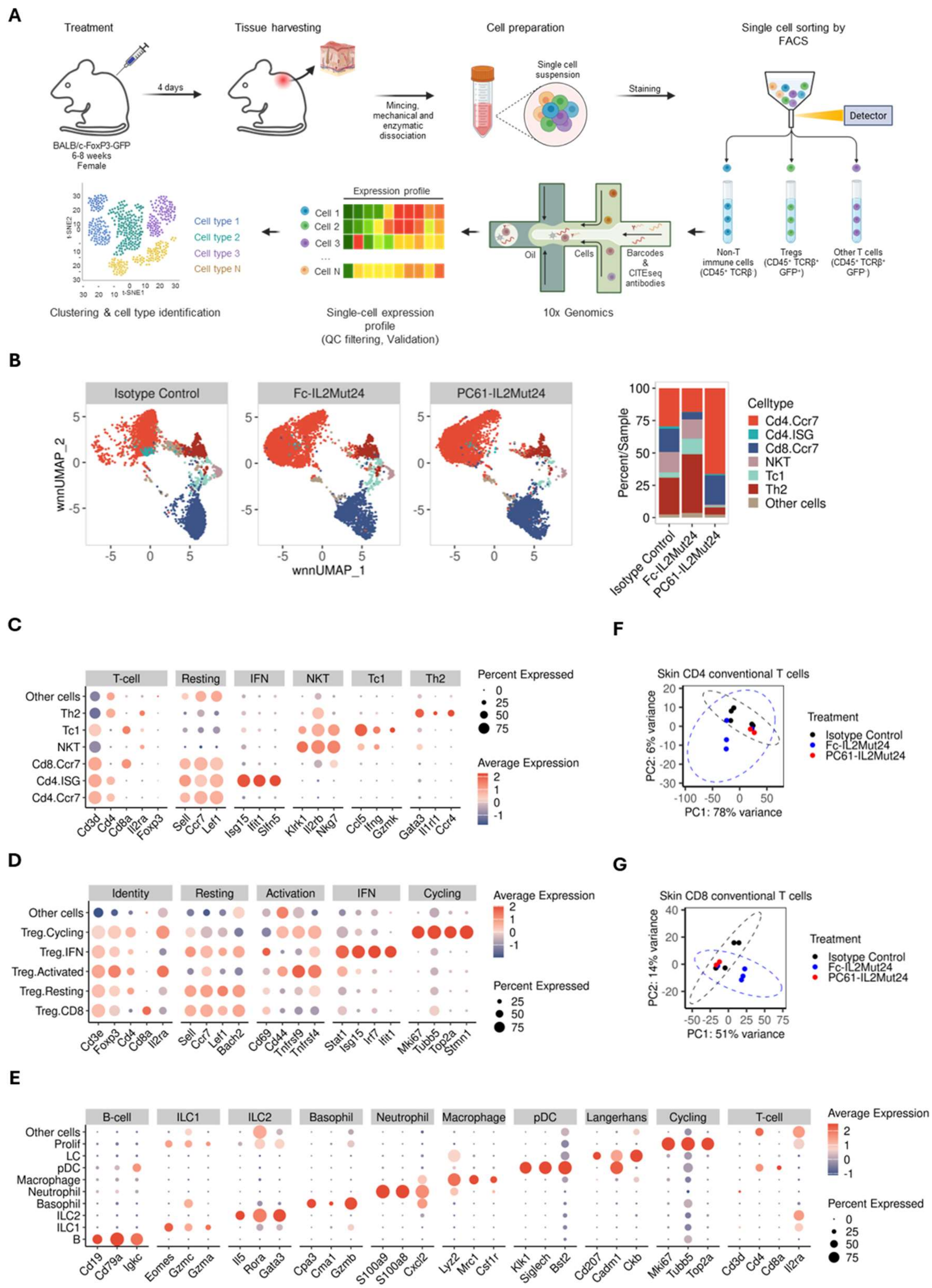

Supplemental Figure 13. Fc-IL2Mut24 and PC61-IL2Mut24 alter the skin immune transcriptional landscape. BALB/c-FoxP3-GFP mice (n=5 mice/group) received a single dorsal subcutaneous dose of isotype control, Fc-IL2Mut24 or PC61-IL2Mut24 (69 pmol). Skin samples were collected and processed on day 4 after dosing. **(A)** Schematic representation of experimental design and downstream skin sample processing methods. **(B)** Uniform Manifold Approximation and Projection (UMAP) of $CD45^+TCR\beta^+GFP^-$ sorted samples (left). Average proportion of different populations from the $CD45^+TCR\beta^+GFP^-$ sorted skin samples in each condition (right). **(C-E)** Cell type markers in the $CD45^+TCR\beta^+GFP^-$ (**C**), $CD45^+TCR\beta^+GFP^+$ **(D)** and $CD45^+TCR\beta^-GFP^-$ **(E)** sorted populations. Bubble size shows the frequency (%) of cells expressing each gene, while color gradient shows gene expression levels. **(F-G)** Principal component analysis (PCA, % confidence interval = 95) of genes expressed by conventional $CD4^+$ T cells **(F)** and $CD8^+$ T cells **(G)** in skin.

Supplemental Figure 14

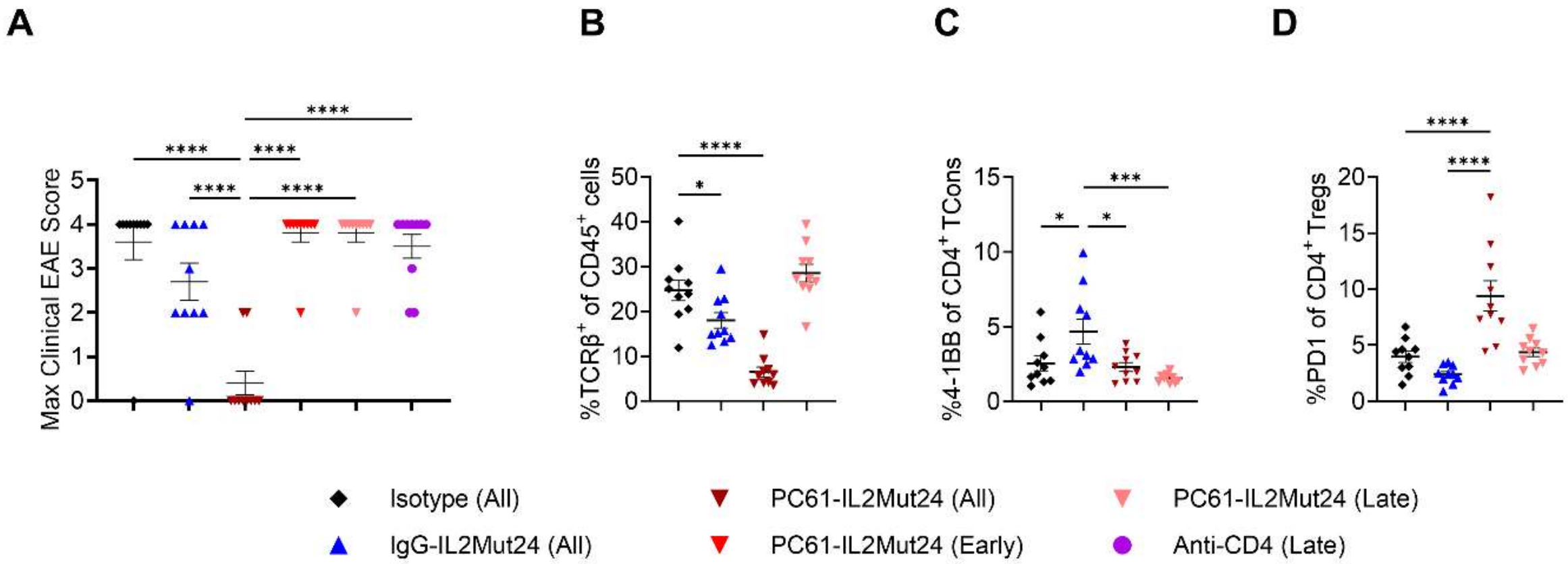

Supplemental Figure 14. PC61-IL2Mut24 outperforms IgG-IL2Mut24 in suppression of EAE onset. C57BL/6J mice (n = 10) were treated i.p. with 100 μL PBS containing isotype, PC61-IL2Mut24, IgG-IL2Mut24, or anti-CD4 on schedules: "All" (Days -2, 2, 6, 9, 13, 16), "Early" (Days 2, 6), or "Late" (Days 15, 19). EAE was induced on Day 0 using 200 μg MOG_{35-55} emulsified in CFA, and 120 ng pertussis toxin was administered i.p. on Days 0 and 2. **(A)** Maximum EAE scores. **(B-D)** At study endpoint, CNS-infiltrating cells were isolated and stained with MOG_{35-55}-IAb Tetramer. Frequency of 4-1BB$^+$ cells among CD4$^+$ conventional T cells **(C)** and PD-1$^+$ cells among CD4$^+$ Tregs **(D)** in the CNS as analyzed by flow cytometry. Data are shown as mean±SEM from n=10 mice per group. Data are representative of two independent experiments. Two -tailed ordinary one-way ANOVA followed by Sidak's post hoc test. *$p < 0.05$, **$p < 0.01$, ***$p < 0.001$, ****$p < 0.0001$.

Supplemental Figure 15:

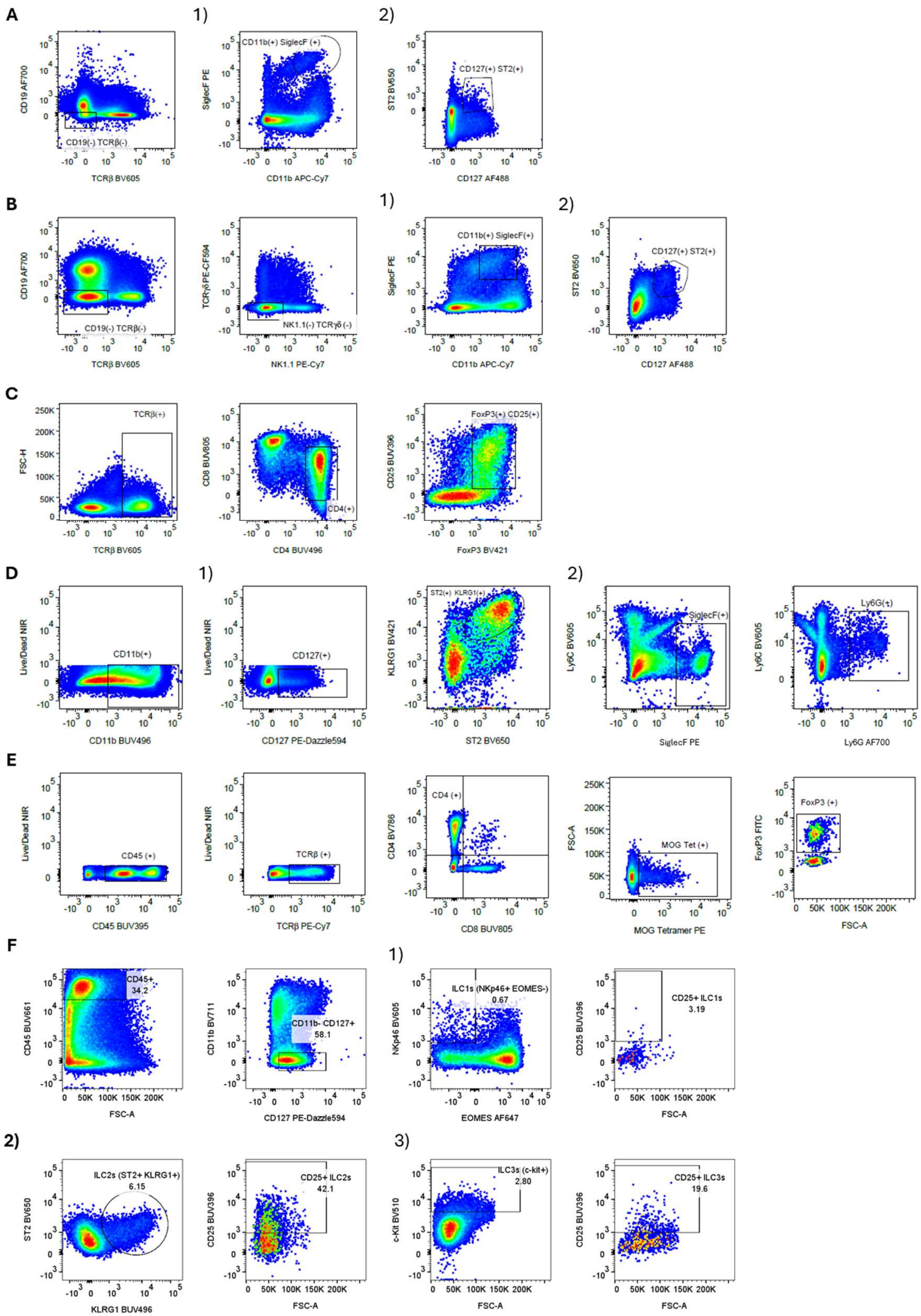

Supplemental Figure 15. Mouse flow cytometry gating strategies. Representative gating strategies used for mouse flow-cytometric analyses in this study. (**A**) For Figure 2H, eosinophils were identified as $CD11b^{+}SiglecF^{+}$ cells among live $CD45^{+}CD19^{-}TCR\beta^{-}$ cells (**A1**). For Figure 2G, ILC2s were identified as $CD127^{+}ST2^{+}$ cells among live $CD45^{+}CD19^{-}TCR\beta^{-}$ cells (**A2**). (**B**) For Figure 3J, eosinophils were identified as $CD11b^{+}SiglecF^{+}$ cells among live $CD45^{+}CD19^{-}TCR\beta^{-}NK1.1^{-}TCR\gamma\delta^{-}$ cells (**B1**); for Figure 3I, ILC2s were identified as $CD127^{+}ST2^{+}$ cells from the same parent population (**B2**). (**C**) Tregs were analyzed within live $CD45^{+}TCR\beta^{+}CD4^{+}$ cells using FoxP3 and CD25. Figure 2F used $FoxP3^{+}$ Tregs; Figure 3C, Figure 6G–H, Supplemental Figure 11D–I, and Supplemental Figure 12C used $CD25^{+}FoxP3^{+}$ Tregs. CD25 MFI in Tregs was quantified in Supplemental Figure 12E. (**D**) Downstream gating used for Figure 4H–J is shown: ILC2s were $ST2^{+}KLRG1^{+}$ among live $CD45^{+}CD11b^{-}CD127^{+}$ cells (**D1**); eosinophils were $SiglecF^{+}$ among live $CD45^{+}CD11b^{+}Ly6C^{-}$ cells and neutrophils were $Ly6G^{+}$ among live $CD45^{+}CD11b^{+}Ly6C^{-}SiglecF^{-}$ cells (**D2**). (**E**) The upstream CNS T-cell gating used for Figure 8E–J and Supplemental Figure 14B–D is shown. $CD4^{+}$ T cells were identified among live $CD45^{+}TCR\beta^{+}$ cells, MOG-$tetramer^{+}$ cells within $CD4^{+}$ T cells, and $FoxP3^{+}$ Tregs within MOG-$tetramer^{+}$ $CD4^{+}$ T cells. (**F**) ILC1s, ILC2s, and ILC3s were identified among live $CD45^{+}CD11b^{-}CD127^{+}$ cells as $NKp46^{+}EOMES^{-}$ (**F1**), $ST2^{+}KLRG1^{+}$ (**F2**), and c-Kit^{+} (**F3**), respectively. These gates were used for Figure 3B, Figure 4C–D, Supplemental Figure 3A–G, and Supplemental Figure 12D; CD25 MFI in ILC2s was quantified in Supplemental Figure 12F.

Supplementary Table 1

Antibody	Isotype	Reactivity	Conjugate	Clone	Supplier	Catalog #
CD45	Rat IgG2b,κ	Mouse	BV786	30-F11	BioLegend	103149
TCRβ	Armenian hamster IgG2,λ1	Mouse	BV605	H57-597	BD Biosciences	562840
CD19	Lewis IgG2a, κ	Mouse	Alexa Fluor 700	1D3	BD Biosciences	557958
CD11b	DA/HA IgG2b, κ	Mouse/human	APC-Cy7	M1/70	BD Biosciences	557657
CD127	Rat IgG2b,κ	Mouse	Alexa Fluor 488	SB/199	BD Biosciences	561533
NK1.1	C3H x BALB/c IgG2a, κ	Mouse	PE-Cy7	PK136	BD Biosciences	552878
CD4	Lewis IgG2b, κ	Mouse	BUV496	GK1.5	BD Biosciences	612952
CD8	LOU/M IgG2a, κ	Mouse	BUV805	53-6.7	BD Biosciences	612898
KLRG1	Syrian Hamster IgG2, κ	Mouse	BV421	2F1	BD Biosciences	562897
ST2	Rat IgG2a, κ	Mouse	BV650	U29-93	BD Biosciences	745403
SiglecF	LOU/M IgG2a, κ	Mouse	PE	E50-2440	BD Biosciences	562068
γδ TCR	Armenian Hamster IgG2, κ	Mouse	PE-CF594	GL3	BD Biosciences	563532
FoxP3	Rat IgG2b, κ	Mouse	FITC	MF-14	BD Biosciences	126406
CD25	Rat IgG1, λ	Mouse	BV711	PC61	BD Biosciences	740714
CD45	LOU/M IgG2b, κ	Mouse	BUV395	30-F11	BD Biosciences	564279
TCRβ	Armenian Hamster IgG2, λ1	Mouse	BV421	H57-597	BD Biosciences	562839
CD4	Rat IgG2a, κ	Mouse	BV786	RM4-5	BioLegend	100551
CD8α	LOU/M IgG2a, κ	Mouse	PE-Cy7	53-6.7	BD Biosciences	561097
FoxP3	Rat IgG2b, κ	Mouse	BV421	MF-14	BD Biosciences	126419
CD25	OFA IgG1, λ	Mouse	BUV395	PC61	BD Biosciences	564022
CD11b	DA/HA IgG2b, κ	Mouse	BUV495	M1/70	BD Biosciences	749864
CD45.2	IgG2a, κ	Mouse	BUV805	104	BD Biosciences	569200
Ly6C	Rat IgM, κ	Mouse	BV605	AL-21	BD Biosciences	563011
TCR	Armenian Hamster IgG	Mouse	AF488	H57-597	BioLegend	109215
Ly6G	Lewis IgG2a, κ	Mouse	AF700	1A8	BD Biosciences	561236
CD127	Rat IgG2a, κ	Mouse	PE-Dazzle594	A7R34	BioLegend	135032
CD45	Rat IgG2b, κ	Mouse	BUV661	30-F11	BD Biosciences	612975
CD4	Rat IgG2a, κ	Mouse	BUV496	RM4-5	BD Biosciences	741050
FoxP3	Rat IgG2a, κ	Mouse/rat	Alexa Fluor 488	FJK-16s	Invitrogen	535773
NKp46	Rat IgG2a, κ	Mouse	BV605	29A1.4	BD Biosciences	564069

EOMES	Rat IgG2b, λ	Mouse	AF647	W17001A	BioLegend	157703
CD11b	Rat IgG2b, κ	Mouse/human	BV711	M1/70	BioLegend	101242
c-Kit	Rat IgG2b, κ	Mouse	BV510	2B8	BioLegend	105839
KLRG1	Syrian hamster IgG	Mouse	BUV496	2F1	Invitrogen	364-5893-82
CD25	Rat IgM, κ	Mouse	FITC	7D4	BD Biosciences	553071
4-1BB	Syrian Hamster IgG	Mouse	BV421	17B5	BD Biosciences	570317
PD-1	Rat IgG2a, κ	Mouse	BV605	29F.1A12	Biolegend	135220
IFNγ	Rat IgG1, κ	Mouse	APC	XMG1.2	Biolegend	505810
CD49a	Armenian Hamster IgG	Mouse	APC	HMα1	BioLegend	142606
CD49b	Armenian Hamster IgG1, κ	Mouse	BV805	HMα2	BD Biosciences	741962
T-cell I-A^b MOG$_{35–55}$ tetramer	NA	Mouse	PE	NA	MBL International	TS-M704-1
CD3	Mouse BALB/c IgG1, λ	NHP	PerCP	SP34-2	BD Biosciences	552851
CD4	Mouse BALB/c IgG1, κ	NHP	BV605	L200	BD Biosciences	562843
FoxP3	Mouse IgG1, κ	NHP	PE	206D	BioLegend	320108
CD25	Mouse IgG2b, κ	NHP	APC	4E3	Miltenyi	130-092-858

Supplementary Table 2

**Cell type**	**Markers**
B cell	*Cd19, Cd79a, Igkc, Mzb1, Ms4a1*
Basophil	*Mcpt8, Fcer1A, Cd200r3, Cpa3, Cma1*
ILC	*Gzmb, Prf1, Klrb1, Fgfbp2, Klrf1, Gzmm, Gzmh, Il2Rb, Klrd1, Il7r, Ifng,Tnf*
ILC1	*Eomes, Tnfrsf9, Klrb1C, Ncr1, Itga1, Il1r1, Gzmc*
ILC2	*Kit, Thy1, St2, Il5, Rora, Icos, Gata3*
ILC3	*Kit, Thy1, Il1r1, Il23r*
LC	*Ckb, Timd4, Cd207*
Macrophage	*Fcgr2a, Csf1r, Sat1, Lyz2, Mrc1*
Neutrophil	*S100a8, S100a9, Fpr2, Cxcr2, Prok2, Ffar2, Fcgr3b, Cxcl2*
pDC	*Clec4c, Jchain, Gzmb, Siglech, Klk1, Cadm1*
Prolif	*Mki67*
CD4 T cell	*Cd4, Cd3d*
Cd4.Ccr7	*Ccr7, Lef1, Sell*
Cd4.ISG	*Isg15, Ifit1*
Th2	*Gata3, Il4, Il13, Il1rl1, Ccr4*
CD8 T cell	*Cd8a, Cd3d*
Cd8.Ccr7	*Ccr7, Lef1, Sell*
Tc1	*Ccl5, Ifng, Gzmk, Gzmb*
NKT	*Cd3d, Nkg7, Il2rb, Klrk1*
Treg	*Cd3d, Foxp3, Il2ra*
Treg.Ctla4	*Ctla4, Tnfrsf9, Stat3, Fth1*
Treg.Il31ra	*Il31ra*
Treg.Itgb7	*Itgb7*
Treg.Lef1	*Lef1, Tcf7*
Treg.Ms4a4	*Ms4a4b, Evl*
Treg.Prolif	*Mki67*
Treg.Tnfrsf4	*Tnfrsf4, Eomes, Egr3*
Treg.Tox	*Tox*
Treg.Cd8	*Cd8a, Cd8b*

Supplementary Table 3

	gene	cluster_id	logFC	p_adj.loc	logCPM	p_val	p_adj.glb	comparison
1	*Amd1*	ILC2	-1.70	3.23E-05	6.71	7.49E-08	1.04E-05	Fc-IL2Mut24 vs Isotype Control
2	*Zc3h12c*	ILC2	-1.54	2.59E-04	7.06	1.32E-06	1.08E-04	Fc-IL2Mut24 vs Isotype Control
3	*Nlrp3*	ILC2	2.64	6.04E-04	5.92	5.49E-06	3.35E-04	Fc-IL2Mut24 vs Isotype Control
4	*Nme1*	ILC2	-1.77	6.78E-04	5.82	6.35E-06	3.71E-04	Fc-IL2Mut24 vs Isotype Control
5	*Nrp1*	ILC2	2.49	1.90E-03	5.71	2.80E-05	1.20E-03	Fc-IL2Mut24 vs Isotype Control
6	*Ctsa*	ILC2	1.65	2.06E-03	5.95	3.31E-05	1.33E-03	Fc-IL2Mut24 vs Isotype Control
7	*Arl5a*	ILC2	1.61	6.16E-03	5.97	1.53E-04	4.32E-03	Fc-IL2Mut24 vs Isotype Control
8	*Samd4*	ILC2	-2.05	8.91E-03	5.06	2.74E-04	6.59E-03	Fc-IL2Mut24 vs Isotype Control
9	*Hspa1a*	ILC2	2.83	1.08E-02	7.53	3.81E-04	8.38E-03	Fc-IL2Mut24 vs Isotype Control
10	*Pde10a*	ILC2	-2.49	1.25E-02	5.89	4.73E-04	9.90E-03	Fc-IL2Mut24 vs Isotype Control
11	*Prkag2*	ILC2	-1.59	1.39E-02	5.65	5.89E-04	1.16E-02	Fc-IL2Mut24 vs Isotype Control
12	*Mthfs*	ILC2	-1.64	1.50E-02	5.16	6.86E-04	1.30E-02	Fc-IL2Mut24 vs Isotype Control
13	*Grik2*	ILC2	2.44	1.77E-02	6.16	8.70E-04	1.52E-02	Fc-IL2Mut24 vs Isotype Control
14	*Ccl5*	ILC2	3.34	2.06E-02	6.16	1.14E-03	1.82E-02	Fc-IL2Mut24 vs Isotype Control
15	*Pomgnt1*	ILC2	-2.08	3.12E-02	6.78	2.08E-03	2.77E-02	Fc-IL2Mut24 vs Isotype Control
16	*Ifitm3*	ILC2	1.73	3.42E-02	6.17	2.38E-03	3.03E-02	Fc-IL2Mut24 vs Isotype Control
17	*Ntn4*	ILC2	-2.38	3.51E-02	4.78	2.47E-03	3.11E-02	Fc-IL2Mut24 vs Isotype Control
18	*Irf8*	ILC2	1.84	3.55E-02	5.74	2.51E-03	3.14E-02	Fc-IL2Mut24 vs Isotype Control
19	*Arid2*	ILC2	-1.72	3.76E-02	5.70	2.71E-03	3.33E-02	Fc-IL2Mut24 vs Isotype Control
20	*Ccr7*	ILC2	2.02	4.53E-02	6.94	3.55E-03	4.00E-02	Fc-IL2Mut24 vs Isotype Control
21	*mt-Atp8*	ILC2	2.60	2.15E-11	9.58	5.74E-15	2.37E-12	PC61-IL2Mut24 vs Isotype Control
22	*Hexb*	ILC2	-1.51	2.37E-06	12.80	1.27E-09	1.11E-07	PC61-IL2Mut24 vs Isotype Control
23	*S100a4*	ILC2	-1.35	3.89E-06	9.79	3.12E-09	2.33E-07	PC61-IL2Mut24 vs Isotype Control
24	*Btbd9*	ILC2	-1.43	9.99E-05	8.73	1.34E-07	5.95E-06	PC61-IL2Mut24 vs Isotype Control

25	*Nkain2*	ILC2	-1.33	2.16E-04	8.40	3.47E-07	1.35E-05	PC61-IL2Mut24 vs Isotype Control
26	*Peak1*	ILC2	-1.43	5.10E-04	7.68	1.36E-06	4.46E-05	PC61-IL2Mut24 vs Isotype Control
27	*Smpdl3a*	ILC2	1.49	1.75E-03	6.99	6.54E-06	1.66E-04	PC61-IL2Mut24 vs Isotype Control
28	*Lars2*	ILC2	-1.30	2.42E-03	12.70	1.36E-05	3.05E-04	PC61-IL2Mut24 vs Isotype Control
29	*Rptor*	ILC2	-1.39	2.83E-03	7.56	1.97E-05	4.16E-04	PC61-IL2Mut24 vs Isotype Control
30	*Hspa1a*	ILC2	3.55	5.12E-03	7.53	4.25E-05	7.93E-04	PC61-IL2Mut24 vs Isotype Control
31	*Vim*	ILC2	1.09	5.42E-03	11.10	4.68E-05	8.58E-04	PC61-IL2Mut24 vs Isotype Control
32	*Nme1*	ILC2	-1.93	1.05E-02	5.82	9.93E-05	1.59E-03	PC61-IL2Mut24 vs Isotype Control
33	*Vamp3*	ILC2	1.72	1.35E-02	5.93	1.52E-04	2.27E-03	PC61-IL2Mut24 vs Isotype Control
34	*Ypel3*	ILC2	1.41	1.35E-02	6.41	1.40E-04	2.12E-03	PC61-IL2Mut24 vs Isotype Control
35	*Zcchc14*	ILC2	-2.65	1.35E-02	5.59	1.58E-04	2.33E-03	PC61-IL2Mut24 vs Isotype Control
36	*Rab18*	ILC2	1.62	1.40E-02	5.96	1.83E-04	2.61E-03	PC61-IL2Mut24 vs Isotype Control
37	*Tuba1c*	ILC2	1.53	1.46E-02	6.63	2.02E-04	2.82E-03	PC61-IL2Mut24 vs Isotype Control
38	*Pecam1*	ILC2	-1.79	1.46E-02	6.80	1.95E-04	2.74E-03	PC61-IL2Mut24 vs Isotype Control
39	*Odc1*	ILC2	1.42	3.54E-02	8.61	7.66E-04	8.46E-03	PC61-IL2Mut24 vs Isotype Control
40	*Rgs10*	ILC2	2.11	3.59E-02	5.75	8.21E-04	8.90E-03	PC61-IL2Mut24 vs Isotype Control
41	*mt-Atp8*	ILC2	1.06	6.79E-05	9.58	1.82E-08	1.32E-05	PC61-IL2Mut24 vs Fc-IL2Mut24
42	*Nlrp3*	ILC2	-3.96	1.58E-03	5.92	8.44E-07	2.80E-04	PC61-IL2Mut24 vs Fc-IL2Mut24
43	*Nrp1*	ILC2	-3.69	3.73E-03	5.71	3.99E-06	9.18E-04	PC61-IL2Mut24 vs Fc-IL2Mut24
44	*Rpl13a*	ILC2	0.95	6.84E-03	8.29	1.19E-05	2.21E-03	PC61-IL2Mut24 vs Fc-IL2Mut24
45	*Arg1*	ILC2	-2.28	3.92E-02	6.82	1.05E-04	1.16E-02	PC61-IL2Mut24 vs Fc-IL2Mut24
46	*Amd1*	ILC2	1.17	4.02E-02	6.71	1.18E-04	1.26E-02	PC61-IL2Mut24 vs Fc-IL2Mut24
47	*Sag*	ILC2	1.60	4.25E-02	6.08	2.83E-04	2.35E-02	PC61-IL2Mut24 vs Fc-IL2Mut24
48	*Palld*	ILC2	1.52	4.25E-02	9.92	1.86E-04	1.74E-02	PC61-IL2Mut24 vs Fc-IL2Mut24
49	*Hgsnat*	ILC2	1.44	4.25E-02	5.92	3.58E-04	2.74E-02	PC61-IL2Mut24 vs Fc-IL2Mut24
50	*Fchsd2*	ILC2	1.36	4.25E-02	7.11	2.06E-04	1.87E-02	PC61-IL2Mut24 vs Fc-IL2Mut24

51	*Bmp2*	ILC2	1.12	4.25E-02	7.63	2.65E-04	2.24E-02	PC61-IL2Mut24 vs Fc-IL2Mut24
52	*Med21*	ILC2	1.03	4.25E-02	7.21	3.77E-04	2.81E-02	PC61-IL2Mut24 vs Fc-IL2Mut24
53	*Tubb4b*	ILC2	0.91	4.25E-02	7.60	2.15E-04	1.93E-02	PC61-IL2Mut24 vs Fc-IL2Mut24
54	*Glrx*	ILC2	-0.91	4.25E-02	8.02	3.42E-04	2.65E-02	PC61-IL2Mut24 vs Fc-IL2Mut24
55	*Arhgap15*	ILC2	-0.92	4.25E-02	8.48	3.69E-04	2.77E-02	PC61-IL2Mut24 vs Fc-IL2Mut24
56	*Tmem176a*	ILC2	-1.02	4.25E-02	7.08	3.37E-04	2.64E-02	PC61-IL2Mut24 vs Fc-IL2Mut24
57	*Myo1e*	ILC2	-1.08	4.25E-02	8.35	2.23E-04	1.99E-02	PC61-IL2Mut24 vs Fc-IL2Mut24
58	*Jdp2*	ILC2	-1.44	4.25E-02	7.37	3.90E-04	2.89E-02	PC61-IL2Mut24 vs Fc-IL2Mut24
59	*Ifi27l2a*	ILC2	-3.09	4.25E-02	7.20	3.62E-04	2.74E-02	PC61-IL2Mut24 vs Fc-IL2Mut24
60	*Zcchc14*	ILC2	-2.28	4.68E-02	5.59	4.50E-04	3.22E-02	PC61-IL2Mut24 vs Fc-IL2Mut24
61	*Nkain2*	Treg	-2.67	1.29E-11	7.07	1.64E-15	1.29E-11	Fc-IL2Mut24 vs Isotype Control
62	*Asxl3*	Treg	-2.48	4.64E-09	5.48	1.18E-12	4.64E-09	Fc-IL2Mut24 vs Isotype Control
63	*Rnaset2b*	Treg	-2.52	9.79E-08	6.69	6.22E-11	9.79E-08	Fc-IL2Mut24 vs Isotype Control
64	*Ifitm3*	Treg	3.22	1.26E-06	6.57	1.93E-09	1.26E-06	Fc-IL2Mut24 vs Isotype Control
65	*Dgkb*	Treg	-2.33	3.09E-06	4.08	5.50E-09	3.09E-06	Fc-IL2Mut24 vs Isotype Control
66	*Bbof1*	Treg	-3.20	1.25E-04	4.02	7.32E-07	1.25E-04	Fc-IL2Mut24 vs Isotype Control
67	*Gab2*	Treg	2.41	3.31E-04	2.70	2.65E-06	3.31E-04	Fc-IL2Mut24 vs Isotype Control
68	*Ifitm1*	Treg	11.10	7.09E-04	3.69	7.02E-06	7.09E-04	Fc-IL2Mut24 vs Isotype Control
69	*Pkd2l2*	Treg	-2.24	7.93E-04	5.59	8.27E-06	7.93E-04	Fc-IL2Mut24 vs Isotype Control
70	*Lgals7*	Treg	1.68	9.92E-04	4.95	1.10E-05	9.92E-04	Fc-IL2Mut24 vs Isotype Control
71	*Ifitm2*	Treg	2.46	9.94E-04	6.94	1.11E-05	9.94E-04	Fc-IL2Mut24 vs Isotype Control
72	*Spata7*	Treg	-2.92	1.60E-03	5.02	2.10E-05	1.60E-03	Fc-IL2Mut24 vs Isotype Control
73	*Slc15a2*	Treg	-2.81	2.54E-03	5.57	4.57E-05	2.54E-03	Fc-IL2Mut24 vs Isotype Control
74	*Ntm*	Treg	2.37	2.88E-03	2.98	5.37E-05	2.88E-03	Fc-IL2Mut24 vs Isotype Control
75	*Dhrs3*	Treg	1.48	9.13E-03	3.67	3.04E-04	9.13E-03	Fc-IL2Mut24 vs Isotype Control
76	*Kcnip2*	Treg	-2.93	1.43E-02	3.47	6.16E-04	1.43E-02	Fc-IL2Mut24 vs Isotype Control

77	*Nckap5*	Treg	2.20	3.26E-02	4.11	2.39E-03	3.26E-02	Fc-IL2Mut24 vs Isotype Control
78	*Aph1c*	Treg	2.03	3.29E-02	3.55	2.45E-03	3.29E-02	Fc-IL2Mut24 vs Isotype Control
79	*Il17a*	Treg	-4.17	3.33E-02	3.28	2.53E-03	3.33E-02	Fc-IL2Mut24 vs Isotype Control
80	*Sh2d4b*	Treg	1.66	4.77E-02	2.95	4.54E-03	4.77E-02	Fc-IL2Mut24 vs Isotype Control
81	*Nme1*	Treg	-2.45	3.30E-06	6.07	8.39E-10	3.30E-06	PC61-IL2Mut24 vs Isotype Control
82	*H2-Q1*	Treg	1.35	1.04E-05	7.85	2.24E-08	1.04E-05	PC61-IL2Mut24 vs Isotype Control
83	*Tpx2*	Treg	-2.45	2.37E-04	3.40	1.14E-06	2.37E-04	PC61-IL2Mut24 vs Isotype Control
84	*Nusap1*	Treg	-2.62	2.57E-04	3.94	1.30E-06	2.57E-04	PC61-IL2Mut24 vs Isotype Control
85	*Birc5*	Treg	-2.62	7.30E-04	4.94	6.67E-06	7.30E-04	PC61-IL2Mut24 vs Isotype Control
86	*Cenpf*	Treg	-2.51	1.15E-03	3.65	1.27E-05	1.15E-03	PC61-IL2Mut24 vs Isotype Control
87	*Bcr*	Treg	1.20	1.79E-03	4.16	2.39E-05	1.79E-03	PC61-IL2Mut24 vs Isotype Control
88	*Clspn*	Treg	-2.33	1.94E-03	3.47	2.78E-05	1.94E-03	PC61-IL2Mut24 vs Isotype Control
89	*Phkg1*	Treg	-2.83	2.31E-03	5.33	3.72E-05	2.31E-03	PC61-IL2Mut24 vs Isotype Control
90	*Chpt1*	Treg	-2.48	2.49E-03	3.58	4.47E-05	2.49E-03	PC61-IL2Mut24 vs Isotype Control
91	*Cables2*	Treg	1.16	2.54E-03	5.14	4.61E-05	2.54E-03	PC61-IL2Mut24 vs Isotype Control
92	*Knl1*	Treg	-2.39	2.79E-03	3.39	5.21E-05	2.79E-03	PC61-IL2Mut24 vs Isotype Control
93	*Lta*	Treg	-2.38	3.28E-03	4.22	6.75E-05	3.28E-03	PC61-IL2Mut24 vs Isotype Control
94	*Prkca*	Treg	1.22	3.88E-03	9.11	9.13E-05	3.88E-03	PC61-IL2Mut24 vs Isotype Control
95	*Lysmd4*	Treg	1.32	4.35E-03	3.15	1.07E-04	4.35E-03	PC61-IL2Mut24 vs Isotype Control
96	*Ntm*	Treg	2.09	6.78E-03	2.98	2.15E-04	6.78E-03	PC61-IL2Mut24 vs Isotype Control
97	*Cenpt*	Treg	1.18	9.15E-03	3.73	3.72E-04	9.15E-03	PC61-IL2Mut24 vs Isotype Control
98	*Epas1*	Treg	1.60	2.84E-02	4.30	2.22E-03	2.84E-02	PC61-IL2Mut24 vs Isotype Control
99	*Tubb2a*	Treg	2.06	2.85E-02	7.65	2.24E-03	2.85E-02	PC61-IL2Mut24 vs Isotype Control
100	*Epb41*	Treg	1.15	3.46E-02	6.95	3.12E-03	3.46E-02	PC61-IL2Mut24 vs Isotype Control
101	*Nkain2*	Treg	1.35	4.33E-07	7.07	5.51E-11	4.33E-07	PC61-IL2Mut24 vs Fc-IL2Mut24
102	*Hexb*	Treg	1.52	1.85E-06	10.80	4.70E-10	1.85E-06	PC61-IL2Mut24 vs Fc-IL2Mut24

103	*Ifitm3*	Treg	-3.04	6.07E-06	6.57	2.31E-09	6.07E-06	PC61-IL2Mut24 vs Fc-IL2Mut24
104	*Asxl3*	Treg	1.25	6.80E-05	5.48	3.46E-08	6.80E-05	PC61-IL2Mut24 vs Fc-IL2Mut24
105	*Kif15*	Treg	-2.02	6.98E-04	3.24	9.76E-07	6.98E-04	PC61-IL2Mut24 vs Fc-IL2Mut24
106	*Gab2*	Treg	-2.04	2.59E-03	2.70	4.56E-06	2.59E-03	PC61-IL2Mut24 vs Fc-IL2Mut24
107	*Dgkb*	Treg	1.38	3.11E-03	4.08	6.21E-06	3.11E-03	PC61-IL2Mut24 vs Fc-IL2Mut24
108	*Ifitm1*	Treg	-5.98	3.11E-03	3.69	6.57E-06	3.11E-03	PC61-IL2Mut24 vs Fc-IL2Mut24
109	*Clspn*	Treg	-2.42	5.22E-03	3.47	1.39E-05	5.22E-03	PC61-IL2Mut24 vs Fc-IL2Mut24
110	*Tpx2*	Treg	-1.93	6.36E-03	3.40	2.45E-05	6.36E-03	PC61-IL2Mut24 vs Fc-IL2Mut24
111	*Nusap1*	Treg	-1.91	1.22E-02	3.94	6.49E-05	1.22E-02	PC61-IL2Mut24 vs Fc-IL2Mut24
112	*Birc5*	Treg	-2.13	1.26E-02	4.94	7.09E-05	1.26E-02	PC61-IL2Mut24 vs Fc-IL2Mut24
113	*Tspan5*	Treg	1.22	1.44E-02	5.62	9.37E-05	1.44E-02	PC61-IL2Mut24 vs Fc-IL2Mut24
114	*Cenpf*	Treg	-1.96	2.07E-02	3.65	1.97E-04	2.07E-02	PC61-IL2Mut24 vs Fc-IL2Mut24
115	*Bbof1*	Treg	1.93	2.46E-02	4.02	2.62E-04	2.46E-02	PC61-IL2Mut24 vs Fc-IL2Mut24
116	*Tubb2a*	Treg	2.58	2.81E-02	7.65	3.42E-04	2.81E-02	PC61-IL2Mut24 vs Fc-IL2Mut24
117	*Clpb*	Treg	1.24	3.33E-02	3.46	4.87E-04	3.33E-02	PC61-IL2Mut24 vs Fc-IL2Mut24
118	*Aspm*	Treg	-2.39	3.45E-02	3.06	5.22E-04	3.45E-02	PC61-IL2Mut24 vs Fc-IL2Mut24
119	*Cbarp*	Treg	1.20	3.86E-02	5.48	6.67E-04	3.86E-02	PC61-IL2Mut24 vs Fc-IL2Mut24
120	*Penk*	Treg	1.92	4.25E-02	7.41	7.94E-04	4.25E-02	PC61-IL2Mut24 vs Fc-IL2Mut24
